# Trajectories of Frailty and Depression and Their Associations with the Risk of Gastrointestinal and Liver Disease: Findings from China Health and Retirement Longitudinal Study and Validation of Survey of Health, Aging and Retirement in Europe

**DOI:** 10.3390/healthcare14131896

**Published:** 2026-06-30

**Authors:** Mingyan Li, Zhenhua Wang

**Affiliations:** Division of Gastroenterology and Hepatology, Shanghai Institute of Digestive Disease, Renji Hospital, School of Medicine, Shanghai Jiao Tong University, 145 Middle Shandong Road, Shanghai 200001, China; limingyan666@sjtu.edu.cn

**Keywords:** gastrointestinal disease, liver disease, depression, frailty

## Abstract

Background: Evidence of the relationship between frailty and depression trajectories and digestive disease in real-world populations remains insufficient. Investigating the long-term dynamic effects of frailty and depression may provide valuable insights for clinical intervention and the precise classification of risk factors for gastrointestinal or liver disease. In the study, we aimed to elucidate the aforementioned association among two representative cohorts. Methods: The CHARLS dataset represents a cohort of 10,303 participants over 40 years of age in China, with a follow-up period from 2011 to 2018. First, a group-based trajectory modeling method was used to identify combined trajectories of frailty and depression over a 7-year follow-up period. Frailty was assessed using the frailty index, while depression was measured using CESD10 scores. Binary logistic models and discrete survival models were applied to explore the associations between combined frailty–depression trajectories and the outcomes of gastrointestinal or liver diseases. Second, after excluding participants with baseline gastrointestinal or liver diseases, a binary logistic regression model was used to analyze the association between baseline frailty and depression and disease outcomes, with the results presented as odds ratios (ORs) and 95% confidence intervals (CIs). Third, Cox proportional hazards models with restricted cubic splines were applied to estimate the association between the baseline frailty index or CESD 10 scores and disease risk, with the results expressed as hazard ratios (HRs) and 95% confidence intervals (CIs). Comprehensive sensitivity analyses and subgroup stratifications supported the findings. The SHARE dataset was used as validation to prove the reliability of the conclusions. The SHARE cohort comprises 5834 participants 40 years of age and older in Europe, with a follow-up period from 2011 to 2017, and uses the frailty index to assess frailty and the EURO-D scale to assess depression. A binary logistic regression model was used to analyze the association between the trajectory groups and disease outcomes after excluding participants with baseline gastrointestinal diseases, with the results presented as odds ratios (ORs) and 95% confidence intervals (CIs). Results: Three distinct combined trajectories were identified in the CHARLS cohort: G1 (59.7%), stable and robust with no depression; G2 (31.5%), moderate persistent frailty and depression; and G3 (8.8%), escalating frailty and high depression. In the fully adjusted binary model, compared with G1, the risk of gastrointestinal disease was elevated in G2 (OR = 1.94, 95% CI: 1.67–2.24) and G3 (OR = 2.73, 95% CI: 2.12–3.53). Similarly, the risk of liver disease was evidently elevated in G2 (OR = 1.72, 95% CI: 1.38–2.13) and G3 (OR = 3.44, 95% CI: 2.50–4.75). The SHARE findings were consistent with those from CHARLS, with three similar trajectory groups identified in the SHARE cohort. Compared with G1, the risk of gastrointestinal disease was evidently elevated in G2 (OR = 2.035, 95% CI: 1.359, 3.048) and G3 (OR = 4.588, 95% CI: 2.561, 8.218) in the fully adjusted model. Conclusions: Trajectories of frailty and depression were significantly correlated with increased occurrence of gastrointestinal and liver disease, with the results remaining robust across various sensitivity analyses and external cohort validations. A limitation of this study is that the outcome measures are based on self-reported data, which may be subject to measurement bias. These findings highlight the importance of sustained integrated physical–mental approaches and precise psychological screening and classification for the prevention and treatment of gastrointestinal and liver disease.

## 1. Introduction

Gastrointestinal and liver diseases represent about one third of the global disease burden, posing a considerable health concern and significantly increasing morbidity and mortality [1]. In China, gastrointestinal and liver diseases affect approximately 120 million and 300 million individuals, respectively [2]. In 2019, the substantial morbidity of gastrointestinal and liver diseases led to 8 million fatalities and a loss of 277 million disability-adjusted life years (DALYs) [3]. Risk factors for gastrointestinal diseases, including stress, eating ultra-processed foods, and Helicobacter pylori infection, can affect patient outcomes [4,5,6], while obesity, stress, and excessive alcohol use are factors that have been reported to influence occurrence in patients with liver diseases [7,8,9]. Identifying and managing relevant risk factors is of great importance for alleviating the burden on patients with gastrointestinal or liver diseases.

Frailty is a geriatric syndrome primarily characterized by diminished physiological reserve [10]. Fundamental research conducted by Hoogendijk et al. (2019) has reported that frailty affects patients with gastrointestinal and liver diseases through immune dysregulation and chronic low-grade mucosal inflammation [11,12]. Epidemiological research conducted by Zhong et al. (2025) using data from the UK Biobank found that frailty is independently relevant to a significantly increased risk of developing chronic liver disease [13], while a prospective cohort study further demonstrated an evident association between frailty and irritable bowel syndrome [14]. Frailty is measured using the frailty index (FI), which measures an individual’s proportion of health impairment items compared to the total number of items examined [15]. The frailty index serves as a useful surrogate marker for accelerated biological aging and is associated with numerous adverse biological activities and outcomes [16].

Depression is a mental disorder characterized by persistent sadness [17]. Research conducted by Ghia et al. (2008) has shown that depression-included impairment of the vagus nerve is a key mechanism for intestinal inflammation in animal models [18], and that the circle of immune-inflammatory response and depression exacerbates liver inflammation [19]. Epidemiological research conducted by Koutny et al. (2026) has additionally demonstrated a significant association between depression and chronic liver disease [20], while a cross-sectional study conducted by Söderquist et al. (2020) showed that depression was an independent predictor of irritable bowel syndrome [21]. The CESD-10 provides a score that is brief and straightforward to comprehend, enabling rapid assessment of the severity of an individual’s depressive symptoms [22].

Previous studies have assessed frailty and depression at a single time point, which may not capture their dynamic changes over time. Moreover, the combined effects of their long-term trajectories on gastrointestinal and liver diseases remain largely unknown. Shao et al. (2024) had previously conducted an analysis of participants from waves 1 to 4 of CHARLS to investigate the association between depression and gastrointestinal disease [23]. An established bidirectional relationship between frailty and depression has also been reported by Qian et al. (2026) [24]. Studies have revealed that frailty in middle-age and older persons frequently coexists with depressive symptoms, with a meta-analysis by Soysal et al. (2017) showing a bidirectional association between frailty and depression among older adults [25]. Alvarez-Bustos et al. (2022) reviewed that frailty and depression are dynamic clinical entities that develop over time [26,27], and the incidence of gastrointestinal and liver diseases markedly rises with age, marking middle-age and older adults as a high-risk demographic [28]. It is plausible that, in the interplay between frailty, depression, and gastrointestinal or liver disease, aging requires further investigation [29]. Zhou et al. (2024) found that frail, elderly patients with depression are prone to a higher risk of gastrointestinal and liver disease compared to frail patients without depression, owing to the additive adverse effects of frailty and depression on these diseases [30].

There is insufficient evidence on the long-term correlation between the combined trajectories of frailty and depression and gastrointestinal or liver diseases, especially evidence drawn from various countries, regions, and ethnic populations [31]. The longitudinal design of the CHARLS and SHARE datasets allows for the analysis of frailty and depression trajectories. In the study, we primarily utilize data from CHARLS (2011–2018) to examine the association between different trajectory patterns of frail and depressive symptoms and the occurrence of gastrointestinal and liver disease within Chinese middle- and old-age populations. In addition, we investigate the independent association between the FI or frail symptoms and incident gastrointestinal or liver disease. The independent correlation between CESD scores or depressive symptoms and onset gastrointestinal or liver disease is also explored. To strengthen the robustness of our findings, data from SHARE (2011–2017) representing European middle- and old-age populations was used to perform a sensitivity analysis for validation.

GBTM is a statistical method designed to analyze the heterogeneity of individual developmental trajectories in longitudinal data [32]. Compared with traditional methods, GBTM can be used to perform more detailed analysis by accounting for diverse change patterns among individuals within a group, thereby segmenting the group into multiple subgroups with distinct developmental trajectories [33].

Exploring the association between distinct trajectory patterns of frailty and depression and gastrointestinal or liver disease among middle-aged and older adults is beneficial for theoretically supporting new insights into the interaction between aging, frailty, depression, and digestive disease, which can provide a more precise stratification and classification of target risk factors and suited interventions for patients with gastrointestinal or liver disease in the aging population.

## 2. Materials and Methods

### 2.1. Study Population

In our study, we used data from two databases: CHARLS and SHARE. CHARLS served as the primary database for investigating the associations between frailty and depression trajectories and gastrointestinal or liver disease. Additionally, to strengthen and validate our findings and extend the conclusions to various regions, countries, and ethnic groups, the SHARE cohort was analyzed to explore the correlation between frailty and depression trajectories and gastrointestinal disease, due to the deficiency of liver disease’s status in the questionnaire.

CHARLS is a nationally representative dataset compiled from a baseline survey conducted nationwide from June 2011 to March 2012 that collected microdata representing households and individuals in China using a stratified, multistage sampling design. The study covered 150 country-level units, 450 village-level units, and approximately 17,000 people in 10,000 households. The Standards for the Reporting of Cohort Studies in Surgery (STROCSS) were followed in this cohort study. All study protocols were approved by the Biomedical Ethics Review Committee of Peking University (IRB00001052-11015 on 20 January 2011, 15 August 2013, 20 May 2015 and 2 June 2018), and every participant provided written informed consent. More details about CHARLS can be found at https://charls.pku.edu.cn/gy/gyxm.htm (assessed on 29 August 2024) [34].

SHARE is a multinational, rolling longitudinal panel survey that has gathered data from participants in Europe since 2004, collecting microdata representing older adults’ health, social networks, family structures, work, and retirement and economic circumstances. All study protocols were approved by the Ethics Council of the Max Planck Society (IRB: No 723/2009 on 8 June 2021), and every participant provided written informed consent. More details about SHARE can be found at https://share-eric.eu/ (assessed on 9 February 2026) [35].

**Figure 1:**
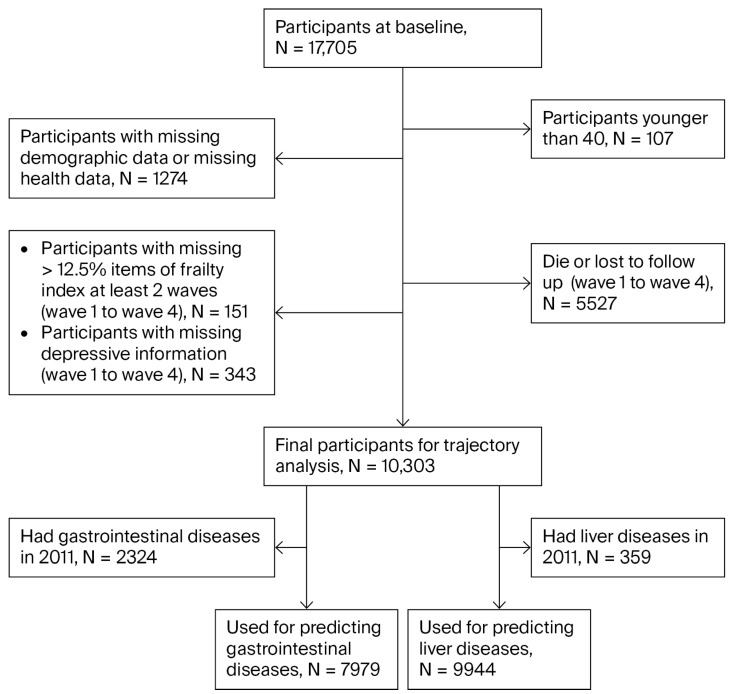
Flowchart of participant selection from CHARLS.

Our study included data from four consecutive survey waves spanning 2011 to 2018, covering 17,705 participants in the CHARLS cohort (Figure 1). First, we excluded participants under the age of 40 years (N = 107). Next, we excluded participants with missing demographic or missing health data (N = 1274); participants missing > 12.5% of items from the frailty index for least two waves (N = 151); and patients missing information on depression (N = 343). We then excluded participants who died or were lost to follow-up (N = 5527). To maximize sample retention, 10,303 participants remained for the trajectory analysis. After excluding participants who had gastrointestinal disease in 2011 (N = 2324), 7979 participants were used for predicting gastrointestinal disease. After excluding participants who had liver disease in 2011 (N = 359), 9944 participants were included for the prediction of liver disease. The baseline characteristics of the included and excluded participants in the CHARLS cohort are detailed in Supplementary Table S1. In the sensitivity study, we included data from four consecutive survey waves from 2011 to 2017. From the initial SHARE sample of 57,982 participants, first, participants younger than 40 years were excluded (N = 122). Next, individuals with missing demographic and health data (N = 2753) were excluded, along with individuals who were missing frailty index data during follow-up or missing information on depression during follow-up (N = 2164). We then excluded participants who died or were lost to follow-up (N = 48,811). After excluding participants with gastrointestinal disease in 2011, 5834 participants were included for the final sensitivity analysis (Supplementary Figure S1).

### 2.2. Frailty Assessment

For the CHARLS dataset, based on previous relevant research, frailty was evaluated using a 32-item frailty index (FI) [36]. Frailty index ranged from 0 to 1, multiplied by 100, with frailty symptoms categorized as yes (FI > 32) or no (FI < 32). For SHARE, frailty was evaluated using a 30-item frailty index (FI), where higher scores imply more severe frailty. Detailed information on the two research cohorts used for the frailty index is shown in Supplementary Figures S2–S4 and Table S2.

### 2.3. Depression Assessment

In the CHARLS cohort, depressive symptoms were measured using the Chinese short form of the CESD-10 (Center for Epidemiologic Studies Depression Scale-10) developed by Radloff [37], a concise and effective tool for assessing depressive mood widely employed in clinical research, in a large community-based sample of older adults in Hong Kong (n = 635), Boey found good internal consistency (α = 0.78) and acceptable test–retest reliability (r = 0.44) for the 10-item Chinese version of CES-D [38]. In our study, the CESD-10 demonstrated a Cronbach’s alpha of 0.80 (95% CI: 0.80–0.81) based on 1000 bootstrap resamples. Ten items about depressive symptoms are included, and each one is evaluated on a four-point scale representing the patient’s depression level for the previous week. The CESD-10 includes the following 10 items: (1) Feeling bothered by things that usually don’t bother me; (2) Having trouble concentrating; (3) Feeling down; (4) Feeling that everything you do requires effort; (5) Feeling hopeful about the future; (6) Feeling fearful; (7) Having trouble sleeping; (8) Feeling cheerful; (9) Feeling lonely; (10) Feeling unable to “get going”. CESD-10 scores ranged from 0 to 30, with higher scores implying more severe depression. Participants with a CESD-10 score higher than 10 were identified as depressed. Detailed information on the research cohort for CESD-10 scores is shown in Supplementary Figure S5. In the SHARE cohort, depressive symptoms were measured using EuroD-12 scores, which is a brief self-report measure of late-life depression developed by a European consortium to facilitate cross-cultural comparisons of depressive symptoms across Europe, Larraga in the validation study of Spanish older adults, reported both high internal consistency (α = 0.75 and 0.79) and acceptable test–retest reliability (weighted κ = 0.60) [39]. The scale ranged from 0 to 12, with higher scores indicating more severe depression. Participants with EuroD-12 scores higher than 4 were classified as depressed (Supplementary Figure S6).

### 2.4. Gastrointestinal and Liver Disease Assessment

In CHARLS, gastrointestinal and liver diseases were assessed through self-report questionnaires and treated as binary variables, with reports from 2018 serving as the basis for the primary analysis. The question asked was “Have you been diagnosed with Liver disease (except fatty liver, tumors, and cancer)” or “Stomach or other digestive disease (except for tumor or cancer)”. In SHARE, gastrointestinal disease was assessed through self-report questionnaires, where the question asked was “Has a doctor ever told you that you had/Do you currently have Stomach or duodenal ulcer, peptic ulcer”.

### 2.5. Covariates

Based on a previous CHARLS study [40], the baseline covariates selected were categorized into demographic factors, socioeconomic factors, and health status factors. Socioeconomic factors comprised social work (yes or no), living alone (yes or no), and work status (yes or no). Health status factors comprised smoking status (never, former, or current), drinking status (never, former, or current), sleep duration (less than 7.5 h/d or more than 7.5 h/d), self-rated health (good, fair, or poor), hypertension (yes or no), diabetes (yes or no), activities in daily living (continuous variable), and instrumental activities in daily living (continuous variable).

Based on a previous SHARE study [41,42], the baseline covariates selected were categorized into demographic factors, socioeconomic factors, and health status factors. Socioeconomic factors comprised living alone (yes or no) and work status (yes or no). Health status factors comprised smoking status (never, former, or current), drinking status (never, former, or current), self-rated health (good, fair, or poor), hypertension (yes or no), diabetes (yes or no), activities in daily living (continuous variable), and instrumental activities of daily living (continuous variable).

### 2.6. Trajectory Group Assessment

Group-based trajectory modeling (GBTM) was used to evaluate the long-term trajectories of frailty and depression over the follow-up period.

First, we constructed models with two to five trajectory groups. The model selection process began by specifying an initial five-group, cubic polynomial model. We initially optimized the model by reducing the number of groups and the polynomial order to determine the model that best fits the data. We then determined the appropriate model based on criteria such as the lowest BIC value, the minimum AIC value, the maximum entropy, the average posterior probability = 70%, and the minimum sample size for each category = 5%. For CHARLS, we identified three groups: Group 1 (G1) was defined as “stable and robust with no depression”, Group 2 (G2) was defined as “moderate persistent frailty and depression”, and Group 3 (G3) was defined as “escalating frailty and high depression”. We adopted the above-mentioned criteria to identify three groups based on the optimal GBTM, and it was evident that the three groups represented in SHARE were similar to those observed in CHARLS. Details on the model selection process are shown in Supplementary Tables S3–S5.

### 2.7. Statistical Analysis

We performed statistical analyses using R 4.5.2 and Stata 18.0. The bilateral *p*-value was considered significant at *p* < 0.05.

We used Stata 18.0 to select the participants included in the study. The baseline characteristics of participants were grouped by trajectories. Categorical variables were reported as counts, and continuous variables were reported as means or medians. Chi-square tests for categorical variables and ANOVA or Kruskal–Wallis rank sum tests for continuous variables were used to analyze the differences among trajectory groups.

We used Stata 18.0 to construct a binary logistic regression model. In accordance with previous research [40], binary logistic models were used to explore the correlation between trajectory groups and gastrointestinal or liver disease in 2018 by calculating ORs and 95% CIs.

We used R 4.5.2 to perform the remaining statistical analyses. For subgroup analyses, we stratified populations based on main demographic variables (e.g., gender, education, age group, marital status, residence) to examine heterogeneous associations between depression and frailty trajectories and gastrointestinal or liver disease. The interaction effects between different subgroups and trajectories were examined.

In accordance with previous research, the proportional hazards (PHs) assumption was applied using the Schoenfeld residuals test for each covariate. Only one variable assumption violation was observed. Cox regression analyses were used to evaluate the association between frailty index or CESD-10 scores and gastrointestinal disease or liver disease. Frailty was classified as none, pre-frail, or frail according to the frailty index calculated at baseline [43]. Depression was classified as none, mild, or severe according to CESD-10 scores calculated at baseline [44]. The independent association between frailty symptoms or depressive symptoms and gastrointestinal disease or liver disease was also evaluated using Cox regression analysis. Hazard ratios (HRs) with 95% confidence intervals (CIs) were calculated using four models whose covariates were in line with the above-mentioned binary logistic regression. The potential nonlinear associations between the frailty index or CESD-10 scores and gastrointestinal or liver disease were plotted visually using restricted cubic splines.

Subgroup analyses were also performed to examine heterogeneous associations between frailty or depressive symptoms and gastrointestinal or liver disease. The interaction effects between different subgroups and frailty symptoms or depressive symptoms were examined. Statistical significance was determined at a two-tailed *p* value < 0.05.

We conducted several sensitivity analyses to test the robustness of our conclusions: (1) To calculate the occurrence of gastrointestinal or liver disease at different time points during the follow-up time, the discrete-time complementary loglog survival model was used to estimate the hazard ratios (HRs) of the association. (2) To clarify the potential influence of drug use on disease occurrence in the CHARLS cohort, we assessed the correlation between frailty and depression trajectories and gastrointestinal or liver disease after excluding patients who had used gastrointestinal or liver drugs or who had gastrointestinal or liver diseases in 2011. GBTM was used to classify trajectories, and a binary logistic model was used to evaluate the relationship between frailty and depression trajectories and gastrointestinal or liver disease. (3) Using the SHARE cohort in 2011 as the baseline and following up until 2017, we assessed the relationship between frailty and depression trajectories and the occurrence of gastrointestinal disease, ultimately including 5834 participants. We validated the analysis using a statistical approach consistent with previous research. (4) To clarify the potential influence of drug use on disease occurrence in the SHARE cohort, we assessed the correlation between frailty and depression trajectories and ulcer disease after excluding patients who had used ulcer drugs or who had gastrointestinal disease in 2011 based on a binary logistic regression model that calculated ORs and 95% CIs.

## 3. Results

### 3.1. Estimated Trajectories of Frailty and Depression Modeling

**Figure 2:**
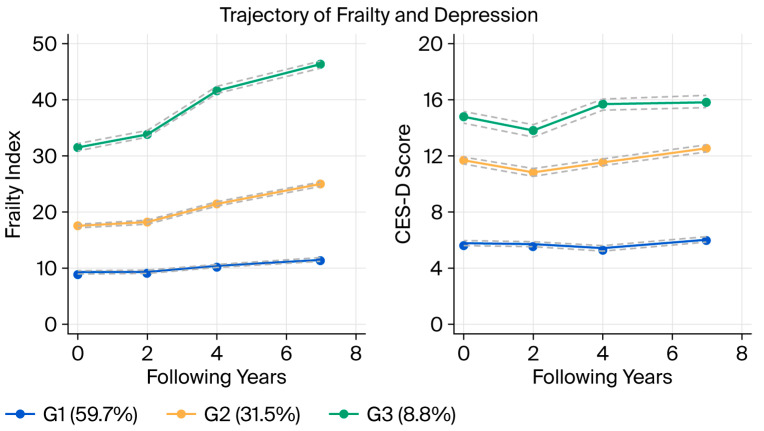
Trajectories of frailty and depression from wave 1 to wave 4 (CHARLS). G1: stable and robust with no depression; G2: moderate persistent frailty and depression; G3: escalating frailty and high depression.

As shown in Figure 2, we identified three trajectory groups using GBTM. The three distinct trajectory groups were defined as follows: G1 was defined as “stable and robust with no depression” (59.7%)—this group displayed a low frailty index and low CESD-10 scores; G2 was defined as “moderate persistent frailty and depression” (31.5%)—this group revealed a gradual increase in the frailty index and CESD scores that approached the cutoff value; G3 was defined as “escalating frailty and high depression” (8.8%)—this group was characterized by a continuously deteriorating frailty index and stable CESD scores.

### 3.2. Baseline Characteristics of Trajectory Groups

The baseline characteristics of participants in the CHARLS cohort classified by trajectory group are presented in Table 1. For the total cohort of 10,303 participants, the mean age was 57.56 (SD 8.73) years, the mean CESD-10 score was 8.32 (SD 6.25), the mean IADL was 0.36 (0.90), and the mean ADL was 0.29 (SD 0.85). A total of 4372 (45.9%) participants were male, and 442 (4.3%) lived alone. In total, 9288 (90.1%) were married, 6366 (61.8%) were current smokers, 3402 (33.0%) were current drinkers, 1148 (11.2%) had a high school education, 3580 (34.7%) lived in urban areas, 7578 (73.6%) participants had a self-rated health status of good, 2487 (24.1%) had hypertension, 575 (5.6%) had diabetes, 4817 (46.8%) engaged in social work, 7121 (69.1%) were working, and 3097 (30.1%) had sufficient sleep.

In comparison to participants in G1 (reference) group, there was a greater likelihood that participants in G2 and G3 had higher IADL scores, a higher frailty index, and higher CESD-10 scores.

### 3.3. Baseline Characteristics

Of the 7979 participants without gastrointestinal disease at baseline, 5151 (64.6%) were in G1, 2271 (28.5%) were in G2, and 557 (7.0%) were in G3. In 2018, after the follow-up time, 1269 participants (15.9%) were diagnosed with gastrointestinal disease, and more than half of the participants belonged to G2 and G3. Participants with gastrointestinal disease were more likely to be female, reside in rural areas, have a lower educational level, and have insufficient sleep time.

Of the 9944 participants without liver disease at baseline, 6024 (60.6%) were in G1, 3067 (30.8%) were in G2, and 853 (8.6%) were in G3. In 2018, after the follow-up time, 533 participants (5.4%) were diagnosed with liver disease, and more than half of the participants belonged to G2 and G3. Participants with liver disease were more likely to reside in rural areas, have a lower educational level, have higher IADL, and have insufficient sleep time (Supplementary Table S6).

### 3.4. Association Between Frailty and Depression Trajectories and Gastrointestinal or Liver Disease

As shown in Table 2, after excluding participants with baseline gastrointestinal or liver diseases, participants in the moderate persistent frailty and depression group (G2) (OR = 2.07, 95% CI [1.82, 2.36], *p* < 0.001) and the escalating frailty and high depression group (G3) (OR = 2.78, 95% CI [2.27, 3.41], *p* < 0.001) were significantly more likely to suffer gastrointestinal disease compared with those in the stable and robust with no depression group in Model 1. Participants in G2 (OR = 1.67, 95% CI [1.38, 2.03], *p* < 0.001) and G3 (OR = 2.98, 95% CI [2.32, 3.83], *p* < 0.001) had a significantly higher risk of developing liver disease than those in G1 in Model 1. The results are also significant in Model 2, Model 3, and Model 4.

### 3.5. Association Between Frailty and Gastrointestinal or Liver Disease

After excluding participants with baseline gastrointestinal or liver diseases, during the 7-year follow-up time, elevated symptoms of frailty exhibited an evident correlation with gastrointestinal or liver disease (Table 3, Supplementary Figures S7 and S8). A total of 1763 instances of gastrointestinal disease occurred. Using none as the reference, the correlations of pre-frailty and frailty with gastrointestinal disease risk remained significant in Model 4, with HRs (95% CI) of 1.51 (1.35–1.68) and 2.10 (1.68–2.62), respectively. A total of 705 instances of liver disease occurred, and the associations of pre-frailty and frailty with liver disease risk remained evident in the fully adjusted model, with HRs (95% CI) of 1.51 (1.26, 1.81) and 2.42 (1.75–3.34), respectively.

**Figure 3:**
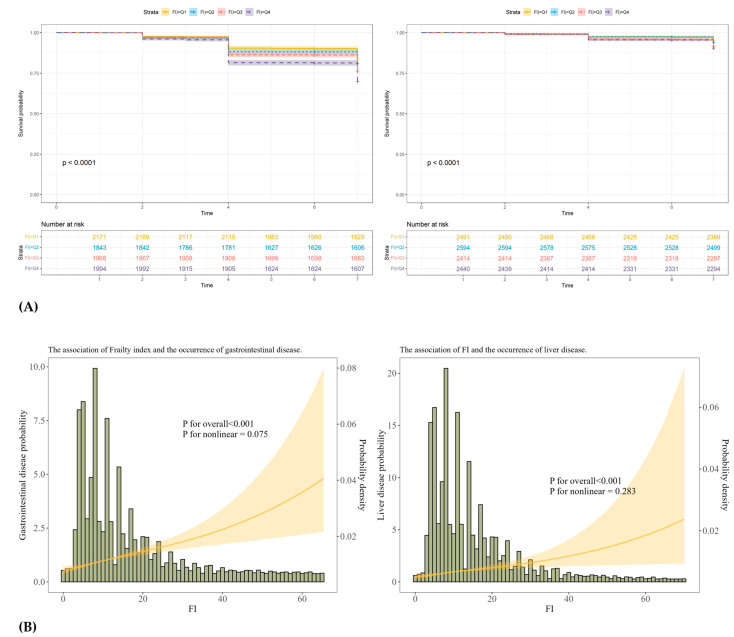
Association of frailty index scores with new-incidence gastrointestinal or liver diseases in the CHARLS participants, determined using RCS analysis. Kaplan–Meier survival curve for new-incidence gastrointestinal or liver disease in CHARLS participants by frailty index quartile. Adjusted for age, gender, education, marital status, residence, social work, living alone, work, self-rated health, smoking status, drinking status, hypertension, diabetes, sleep duration, IADL, and ADL. (**A**) Kaplan–Meier survival curve for new-incidence gastrointestinal or liver disease in the CHARLS participants by frailty index quartiles. (**B**) Restricted cubic splines for new-incidence gastrointestinal or liver disease in CHARLS participants by frailty index.

After excluding participants with baseline gastrointestinal or liver diseases, during the 7-year follow-up period, Table 4 presents the associations between the frailty index, as both a continuous variable and by quartiles, and the occurrence of gastrointestinal or liver disease after excluding participants with baseline gastrointestinal or liver disease (Supplementary Figures S9 and S10). Each unit increase in the frailty index was associated with a 4% higher risk of gastrointestinal disease and a 2% higher risk of liver disease in Model 4. The restricted cubic spline analysis represented the nonlinear correlation between the frailty index and new-onset gastrointestinal disease or liver disease. Kaplan–Meier survival curves for new-onset gastrointestinal diseases stratified by frailty index quartiles demonstrated significant differences in the incidence rates of new-onset gastrointestinal disease and liver disease during follow-up (log-rank *p* < 0.001) (Figure 3).

### 3.6. Association Between Depression and Gastrointestinal or Liver Disease

After excluding participants with baseline gastrointestinal or liver diseases, during the 7-year follow-up period, elevated symptoms of depression exhibited an evident correlation with gastrointestinal or liver disease (Table 5, Supplementary Figures S11 and S12). A total of 1750 instances of gastrointestinal disease occurred. Using none as the reference, the correlations of mild and severe depression with gastrointestinal disease risk remained significant in Model 4, with HRs (95% CI) of 1.31 (1.17–1.48) and 1.66 (1.44–1.91), respectively. A total of 701 instances of liver disease occurred. The associations of mild and severe depression with liver disease risk remained evident in Model 4, with HRs (95% CI) of 1.22 (1.01, 1.47) and 1.36 (1.09–1.70), respectively.

After excluding participants with baseline gastrointestinal or liver diseases, during the 7-year follow-up period, Table 6 presents the associations between CESD10 scores, as both a continuous variable and by quartiles, and the occurrence of gastrointestinal or liver disease after excluding participants with baseline gastrointestinal or liver diseases (Supplementary Figures S13 and S14). Each unit increase in the CESD10 scores was associated with a 4% higher risk of gastrointestinal disease and a 2% higher risk of liver disease in Model 4. The restricted cubic spline analysis represented the nonlinear correlation between CESD10 scores and new-occurrence gastrointestinal disease or liver disease.

**Figure 4:**
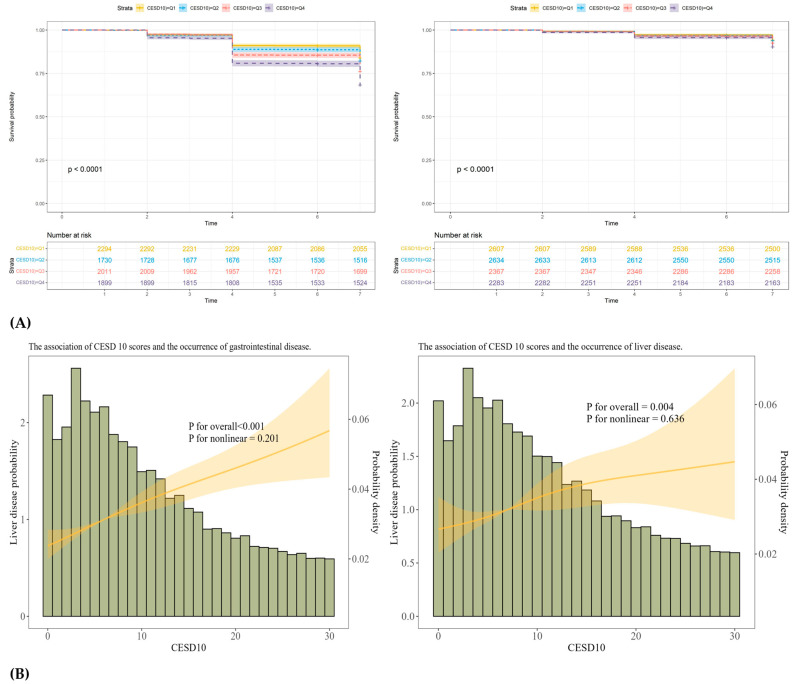
Association between CESD10 scores and new-incidence gastrointestinal or liver diseases in CHARLS participants, determined using RCS analysis. Kaplan–Meier survival curve for new-incidence gastrointestinal or liver disease in CHARLS participants by CESD10 scores quartile. Adjusted for age, gender, education, marital status, residence, social work, living alone, work, self-rated health, smoking status, drinking status, hypertension, diabetes, sleep duration, IADL, and ADL. (**A**) Kaplan–Meier survival curve for new-incidence gastrointestinal or liver disease in the CHARLS participants by CESD10 score quartiles. (**B**) Restricted cubic splines for new-incidence gastrointestinal or liver disease in CHARLS participants by CESD10 scores.

Kaplan–Meier survival curves for new-onset gastrointestinal diseases stratified by CESD10 quartiles demonstrated significant differences in the incidence rates of new-onset gastrointestinal disease and liver disease during follow-up (log-rank *p* < 0.001) (Figure 4).

### 3.7. Subgroup Analyses

**Figure 5:**
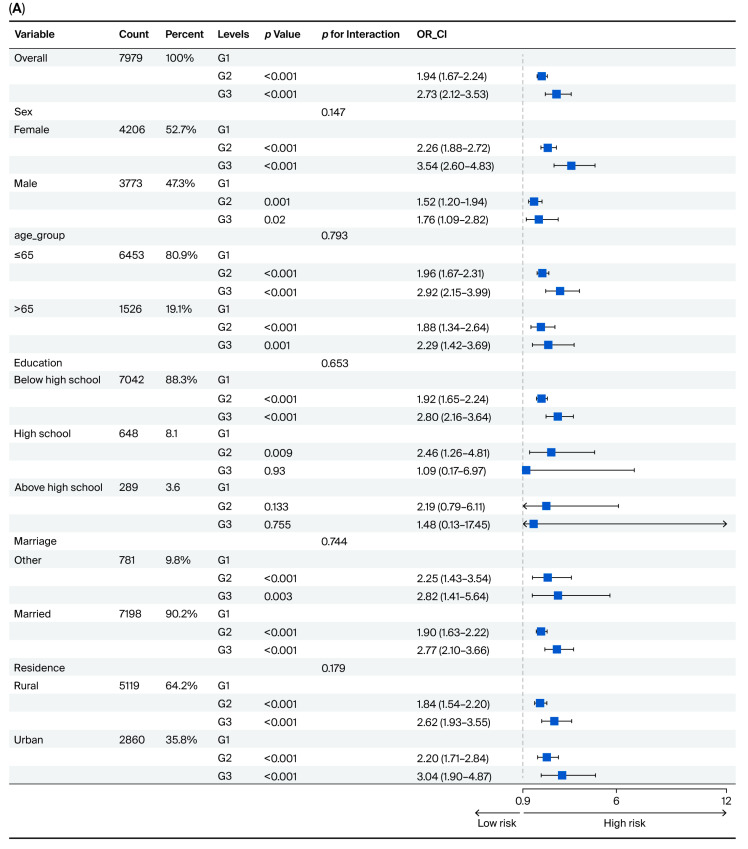

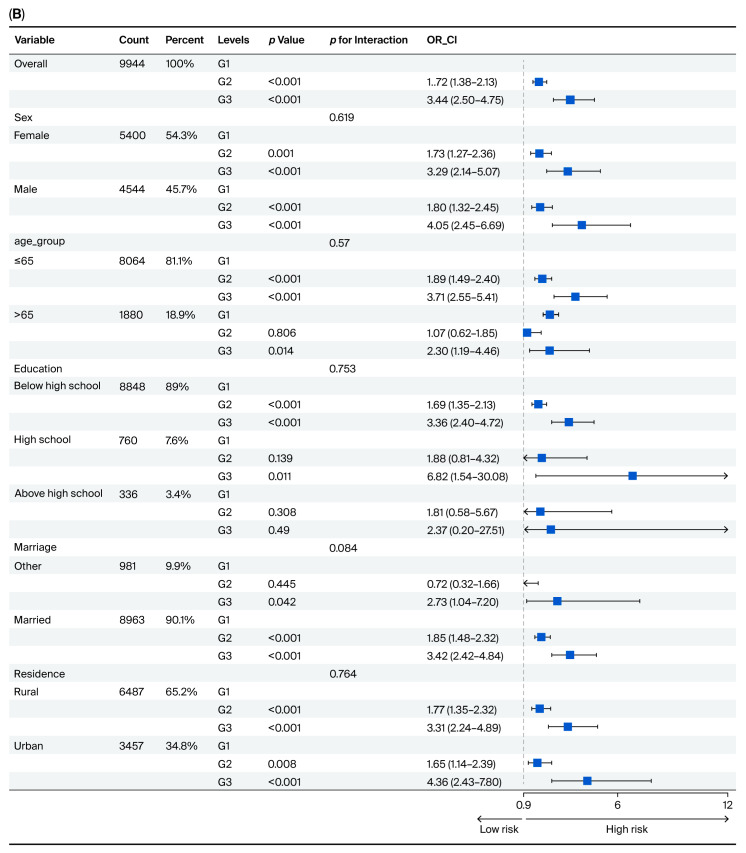
Subgroup and interaction analyses of the associations between frailty and depression trajectories and gastrointestinal or liver disease. (**A**) Subgroup and interaction analyses of the association between frailty and depression trajectories and gastrointestinal disease. (**B**) Subgroup and interaction analyses of the association between frailty and depression trajectories and liver disease. Adjusted for age, gender, education, marital status, residence, social work, living alone, work, self-rated health, smoking status, drinking status, hypertension, diabetes, sleep duration, IADL, and ADL.

Our research included subgroup analyses evaluating the potential impacts of modifying gender, age group, education, marriage, and residence (Figure 5). In the CHARLS cohort, the relationship between depression and frailty trajectories and new-onset gastrointestinal disease occurrence remained, with no significant interaction effects observed. The association between frailty symptoms and gastrointestinal disease remained, with no significant interaction effects observed, except for gender. The association between frailty symptoms and liver disease remained, with no significant interaction effects observed (Supplementary Figure S15). Depressive symptoms showed no interactions for either outcome (Supplementary Figure S16). In the SHARE cohort, depressive symptoms and gastrointestinal disease had no interactions, except for age, education, and residence (Supplementary Figure S17).

### 3.8. Sensitivity Analysis

**Figure 6:**
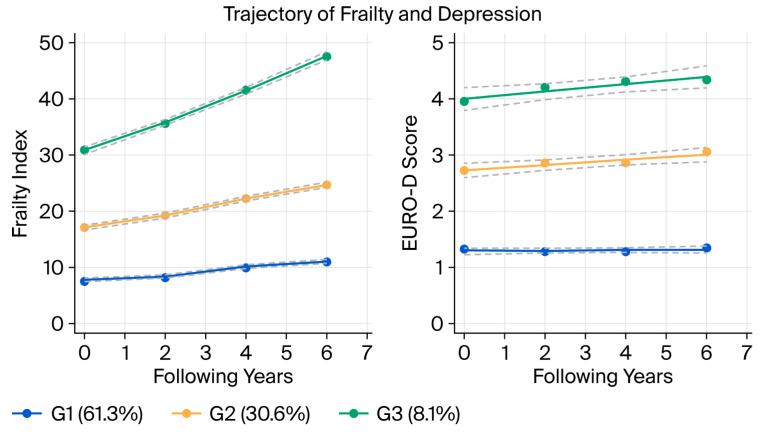
Trajectories of frailty and depression from wave 1 to wave 4 (SHARE). G1: stable and robust with no depression; G2: moderate persistent frailty and borderline depression; G3: escalating frailty and depression.

For comparison, we conducted various sensitivity analyses to enhance the robustness of our findings. (1) Two discrete-time complementary loglog survival models were constructed to estimate hazard ratios. For Model 4, participants in G2 (HR = 1.64, 95% CI [1.46, 1.83], *p* < 0.001) and G3 (HR = 2.11, 95% CI [1.75, 2.55], *p* < 0.001) had significantly greater risks of liver disease compared with those in G1 (Supplementary Table S7). To complement the evidence for this association, the independent effect of baseline frailty and depression measured at a single time point on gastrointestinal or liver disease was assessed using binary logistic regression. (2) The correlation between frailty and depression trajectories and gastrointestinal or liver disease after excluding participants using gastrointestinal or liver drugs at baseline and excluding participants with baseline gastrointestinal disease remained stable when using logistic regression models (Supplementary Figures S18 and S19, Supplementary Tables S8–S15). (3) Using the SHARE database in 2011 and follow-up until 2017 (Supplementary Table S16), we identified three trajectories of frailty and depression, which was similar to the trajectories identified in the CHARLS cohort (Figure 6, Supplementary Tables S17–S19). G1 was defined as “stable and robust with no depression” (61.3%)—this group displayed a low frailty index and low EURO-D scores; G2 was defined as “moderate persistent frailty and borderline depression” (30.6%)—this group revealed a gradual increase in the frailty index and EURO-D scores that nearly approached the cutoff value; G3 was defined as “escalating frailty and depression” (8.1%)—this group was characterized by a continuously deteriorating frailty index and stable EURO-D scores (Table 7 and Table S20). (4) The relationship between frailty and depression trajectories and gastrointestinal disease after excluding participants using gastrointestinal drugs at baseline in the SHARE cohort remained stable in the logistic regression models (Supplementary Figure S20 and Tables S21–S24).

## 4. Discussion

In our study, we utilized data from CHARLS to conduct a longitudinal follow-up analysis of 10,303 participants over a period of seven years. We identified three different combined trajectories of frailty and depression (G1–G3). G1 was defined as “stable and robust with no depression” (59.7%); G2 as “moderate persistent frailty and depression” (31.5%); G3 as “escalating frailty and high depression” (8.8%).

After excluding participants with baseline gastrointestinal or liver disease, the results of our binary logistic regression model showed that moderate persistent frailty and depression (G2, OR = 2.07) and escalating frailty and high depression (G3, OR = 2.78) were significantly associated with gastrointestinal disease in Model 1. G2 (OR = 1.67) and G3 (OR = 2.98) were additionally strongly associated with liver disease in Model 1. In the sensitivity analyses, when frailty and depression were estimated as a single time point, the relation was still consistent. The discrete-time survival model was applied to this study to predict the hazard ratios of gastrointestinal or liver disease within the follow-up time interval (2013, 2015). The findings from the discrete-time survival complementary loglog model concur with the results of the binary logistic regression. After excluding participants with gastrointestinal or liver diseases or who used gastrointestinal or liver drugs at baseline, we conducted several sensitivity analyses, and the results were in line with the primary analysis. G3 showed stronger associations with gastrointestinal and liver diseases than G2, indicating that individuals with more severe frailty and depression face a greater burden of gastrointestinal and liver diseases. In this study, nearly 40.3% of participants were included in G2 and G3. These groups need particular attention, and interventions designed to provide psychological screening and emotional and social support for this population may help reduce the likelihood of gastrointestinal and liver diseases.

Numerous studies have explored the mechanisms underlying the association between frailty and depression trajectories and outcomes of gastrointestinal and liver diseases, and the existing literature proposes several potential mechanisms through which frailty influences gastrointestinal diseases. Lang et al. (2009) [45] discussed the intestinal characteristics of frailty include dysregulated immune responses, a state of chronic low-grade mucositis, increased permeability, impaired absorption, and alterations in the gut microbiota. Inflammatory aging additionally affects intestinal immunity, and when damage predominates, frailty occurs [45]. Serra-Prat et al. (2009) [46] designed a clinical study and found that regarding intestinal absorption, frail patients exhibit impaired gastrointestinal motility, gallbladder alterations, and changes in gallbladder contractile proteins, whether fasting or postprandial [46]. Peterson et al. (2014) [47] reviewed the literature and found that in terms of intestinal permeability, frail patients have impaired epithelial gastrointestinal barrier function, with increased intestinal cell apoptosis, proliferation, and permeability [47]. Furthermore, Jackson et al. (2016) [48] found that frailty is significantly negatively correlated with gut microbial diversity [48], and numerous reports have also described mechanisms linking frailty and the liver, with frailty and chronic liver disease sharing multiple core pathophysiological pathways: insulin resistance, chronic low-grade systemic inflammation, sarcopenia-associated ectopic fat accumulation, vitamin D deficiency, and oxidative stress imbalance. Dziegielewska-Gesiak et al. (2023) [49] reviewed the literature and found that these are not only central mechanisms in the onset and progression of frailty but also key drivers of the development of MASLD and its progression to liver fibrosis and even liver cancer [49]. Sun et al. (2026) [50] found that depression and gastrointestinal diseases share common neurobiological pathways, including the disruption of HPA axis negative feedback, abnormal regulation of the 5-HT system, gut microbiota imbalance, dysregulation of inflammatory cytokine profiles, and abnormal secretion of brain–gut peptides. Chronic psychological stress disrupts the hepatic “tryptophan–kynurenine metabolism” pathway via the “catecholamine–ADRB2–QPRT” axis, thereby impairing CD8^+^T-cell mitochondrial function and accelerating the development of liver cancer [50].

In our study, we found that moderate persistent frailty and depression (G2), as well as severe frailty and high levels of depression (G3), were associated with gastrointestinal and liver diseases. Frailty and depression indicators in a multidimensional model remained associated with these conditions, a finding that is consistent with previous research. The association between frailty and gastrointestinal diseases is well documented in the literature. In prospective cohorts, Zhang et al. (2023) found that frailty and pre-frailty states have been associated with the risk of late-onset IBD [51]. Su et al. (2023) analyzed data from the UK Biobank indicate that frailty significantly increases the risk of gastrointestinal hemorrhage [52]. Additionally, higher frailty indices are associated with poor outcomes following tumor resection in elderly patients with gastrointestinal cancer, and the prevalence of frailty is high in gastric cancer and has an adverse impact on patient outcomes [53].

The association between frailty and liver disease has been extensively studied in the literature. Zhang et al. (2025) designed the epidemiological analyses utilized a cohort from the UK Biobank found that baseline frailty was associated with an increased risk of chronic liver disease [54]. The frailty index is an independent predictor of cirrhosis progression, mortality, and unplanned hospitalization, and is applicable to patients with both compensated and decompensated cirrhosis. Porter et al. (2024) found frailty is associated with an increased risk of in-hospital mortality, complications, and resource utilization among liver transplant recipients [55].

Numerous studies have reported an association between depression and gastrointestinal disorders. Zheng et al. (2026) designed a three-phase study using a UK cohort found that depression is a modifiable risk factor for gastrointestinal disorders [56]. Loftus et al. (2011) conducted a retrospective study found that young patients with Crohn’s disease had a significantly higher risk of anxiety disorders and depression compared with a control group without Crohn’s disease [57]. Eustis et al. (2022) conducted the US National Health and Nutrition Examination Survey found that people with gastrointestinal symptoms were more likely to experience depressive symptoms [58].

Numerous reports have highlighted the association between depression and liver disease. Gu et al. (2022) conducted a systematic review and revealed that depression may increase the risk of non-alcoholic fatty liver disease in women [59] and that there is a strong association between non-alcoholic fatty liver disease and depression, with the two conditions interacting and presenting a higher combined risk. In a South Korean cohort study of adults conducted by Mohamed et al. (2014) [60], depression was associated with an increased risk of hepatic steatosis. A review of studies identified via electronic databases and manual searches found a high prevalence of depression among liver transplant candidates [60].

Our findings align with prior research on frailty and depression trajectories in aging populations. For example, Yuan et al. (2023) designed a study utilizing the CHARLS dataset identified four distinct trajectories that encompass frailty, cognitive decline, and depression [61]. Hu et al. (2025) conducted an analysis from HRS identified five trajectory groups of frailty and depression, ranging from stable and robust health to increasing frailty accompanied by persistent depression [62]. Trajectories provided a more accurate representation of the dynamic changes in frailty and depression in contrast to the independent effects of frailty and depression assessed at a single time point. The primary trajectory group in our study was stable and robust with no depression (G1), comprising 59.7% of the total participants. This indicated that these participants sustained a relatively stable and suboptimal state of health over an extend period.

In the fully adjusted model, G3 demonstrated the highest odds ratio (OR) for gastrointestinal disease compared with G1 using the binary logistic regression model. The analysis of the three trajectory groups indicated that participants in G3 showed escalating frailty, aligned with high depression. This may arise from the bidirectional relation between frailty and depression. Future studies employing Mendelian randomization or interventional designs are needed to test causality and directly quantify the proposed mediators. Previous research has documented the mechanisms underlying this bidirectional correlation, while Deng et al. (2023) conducted a meta-analysis has indicated that depression and frailty interact among older adults [63]. Fu et al. (2024) conducted ligand-binding domain-associated scoring regression analysis that revealed a shared genetic basis for depression and frailty in genome-wide association study data (GWAS) [64]. Barlattani reviewed the literature and found the identified mechanism and findings may be able to elucidate the combined influence of frailty and depression trajectories on the heighted risk of gastrointestinal disease. From a broader perspective, based on emerging neurobiological frameworks [65], Jiang et al. (2024) found that there is an association between inflammatory markers, brain volume, and episodic depression: brain volume in these regions and three inflammatory markers—C-reactive protein, neutrophils, and white blood cells—significantly mediate the association between frailty and depression [66]. However, the bidirectional associations between frailty and depression should be interpreted as correlational rather than causal. Although inflammation, neuroendocrine dysregulation, and gut–brain axis alterations represent plausible mechanistic pathways, none of these mediators were directly measured. Therefore, our mechanistic discussion remains speculative and hypothesis-generating.

Three similar trajectory groups were identified in the CHARLS and SHARE cohorts, and the direction of association with gastrointestinal diseases was consistent, although there were differences in the odds ratios (for example, the OR for G3 in relation to gastrointestinal diseases was 5.032 in the SHARE cohort, which was higher than the 2.78 observed in the CHARLS cohort). This discrepancy may be partly attributable to differences in the cultural backgrounds of Chinese and European populations, the use of different depression scales in the two studies, and cultural variations in the perception of frailty and depression.

In our study, we also explored the relationship between frailty or depression and gastrointestinal or liver disease occurrence in middle-age and older Chinese individuals from static perspectives utilizing the CHARLS database. The results from the static single-node follow-up study showed that, compared with participants who were not frail, pre-frail and frail participants at baseline were at a higher risk of developing gastrointestinal or liver disease. Compared with participants without depression, participants with mild or severe depression at baseline were at a higher risk of suffering gastrointestinal or liver disease. The results of our study suggest that FI or CESD10 scores can serve as a biomarker for stratifying gastrointestinal or liver disease. Using data from 7979 participants, this study found a robust and significant positive linear relationship between the FI and the risk of gastrointestinal disease. Using data from 9494 participants, the study found a positive linear correlation between the FI and the risk of liver disease. RCS analyses were used to confirm and plot clear relationships. These findings are consistent with previous research. Peng et al. (2025) conducted a prospective cohort study using the UKB revealed an association between early frailty status and the risk of suffering gastroesophageal reflux disease [67], while Liu et al. (2023) conducted additional research based on data from the UK Biobank and revealed that frailty is associated with an elevated risk of liver disease [68]. Furthermore, Li et al. (2024) conducted meta-analyses have suggested that depression elevates the incidence of non-alcoholic fatty liver disease (NAFLD) [69]. Zhang et al. (2023) conducted the UKB prospective cohort study and found that both frailty and pre-frailty to be associated with an increased risk of developing inflammatory bowel disease in older adults [51]. Wang et al. (2024) conducted the correlation analysis and revealed that depressive symptoms were positively related to sleep disorders, somatic symptoms, and FGIDs [70]. The CHARLS cohort study indicates that, among middle-age and elderly individuals in China, those exhibiting moderate worsening, high improvement, or high stability of depressive symptoms over time face an increased risk of chronic liver disease.

The study has several strengths: First, the participants included in the cohort were a representative sample of adults age 40 and older, with a focus on China’s local population for greater targeting. Second, the GBTM offered a thorough and precise representation of patterns of frailty and depression in the follow-up period. Third, the study analyzed associations using binary logistic regression and discrete-time survival models, effectively addressing the censored data of the longitudinal study. Fourth, sensitivity analyses were conducted on the primary analysis to exclude the impact of participants’ baseline use of gastrointestinal and liver drugs on disease outcomes. Fifth, this study utilized the SHARE database, with results generalized to different regions and populations. Sixth, we supplemented the association between frailty or depression as independent symptoms and frailty indices or depression scores with gastrointestinal and liver diseases. Cox proportional hazards regression analysis demonstrated that frailty or depression was associated with gastrointestinal or liver diseases, while Kaplan–Meier survival analysis revealed a progressive increase in the incidence of gastrointestinal and liver diseases from the Q1 to Q4 quartiles. Seventh, we conducted subgroup analyses to assess the consistency of results across different population characteristics.

On the other hand, this study has several limitations: Firstly, the diagnosis of gastrointestinal and liver diseases was primarily determined through self-reporting, raising concerns regarding diagnostic reliability and recall bias. Self-reporting is commonly used in large cohort studies, and any non-differential misclassification would likely attenuate the true association toward the null. Prior validation studies support this interpretation [71]. Secondly, the analyzed sample constituted 58.5% of the total CHARLS cohort. The included sample exhibited lower educational attainment and higher smoking prevalence compared to the excluded group, indicating a potential selection bias due to attrition. Thirdly, the study was restricted to individuals 40 years of age and above. While this enhances the internal validity for the specific sample, it limits the generalizability of findings to younger populations. Fourth, despite our efforts to adjust for a wide range of covariates, residual confounding (e.g., inflammatory markers, dietary patterns, or medication adherence) cannot be fully excluded. Fifth, the mechanistic interpretations of the bidirectional relationship between frailty and depression in Section 4 were not directly measured in the study.

Our findings underscore the importance of the early detection and management of frailty and depression as a potential strategy for mitigating gastrointestinal and liver diseases. For patients with these conditions, frailty- and depression-based risk stratification enables clinicians to tailor preventive strategies, potentially incorporating integrated interventions targeting frailty and depression. This provides a theoretical basis for exploring frailty and depression as risk factors for gastrointestinal and liver diseases.

## 5. Conclusions

In our study, we utilized the CHARLS and SHARE databases to investigate three trajectory groups of frailty and depression and then analyze the association between dynamic changes in frailty and depression and gastrointestinal and liver disease in adults 40 years of age and older in various territories and ethnic populations. In the CHARLS cohort, G3 was more strongly associated with gastrointestinal and liver diseases than G2, suggesting that individuals with more severe frailty and depressive symptoms face a greater burden of gastrointestinal and liver diseases. The current findings underscore the significance of clinical screening for fluctuations in frailty and depression, as these conditions markedly elevate the risk of gastrointestinal and liver disease. Interventions designed to enhance and alleviate mental health and adaptive capacity may contribute to a decrease in the incidence of gastrointestinal and liver diseases. More detailed risk stratification factors for gastrointestinal and liver diseases can be investigated in the future based on our research.

## Figures and Tables

**Table 1:** Baseline characteristics of trajectory groups for frailty and depression (CHARLS).

Variables	Overall	G1	G2	G3	*p*
Number	10,303 (100.0%)	6190 (60.1%)	3208 (31.1%)	905 (8.8%)	
Age, years	57.56 (8.73)	56.21(8.39)	58.87 (8.79)	62.16 (8.58)	<0.001
Gender					<0.001
Male	4732 (45.9%)	3359 (54.3%)	1090 (34.0%)	283 (31.3%)	
Female	5571 (54.1%)	2831 (45.7%)	2118 (66.0%)	622 (68.7%)	
Education					<0.001
Below high school	9155 (88.9%)	5271 (85.2%)	3014 (94.0%)	870 (96.1%)	
High school	789 (7.7%)	628 (10.1%)	135 (4.2%)	26 (2.9%)	
Above high school	359 (3.5%)	251 (4.7%)	59 (1.8%)	9 (1.0%)	
Marital status					<0.001
Married	9288 (90.1%)	5755 (93.0%)	2779 (86.6%)	754 (83.3%)	
Other	1015 (9.9%)	435 (7.0%)	429 (13.4%)	151 (16.7%)	
Residence					<0.001
Urban	3580 (34.7%)	2416 (39.0%)	930 (29.0%)	234 (25.9%)	
Rural	6723 (65.3%)	3774 (61.0%)	2278 (71.0%)	671 (74.1%)	
Living alone					0.004
Yes	442 (4.3%)	3116 (50.3%)	1367 (42.6%)	334 (36.9%)	
No	9861 (95.7%)	3074 (49.7%)	1841 (57.4%)	571 (63.1%)	
Self-rated health					<0.001
Good	7578 (73.6%)	5379 (86.9%)	1889 (58.9%)	310 (34.3%)	
Fair	2306 (22.4%)	747 (12.1%)	1126 (35.1%)	433 (47.8%)	
Poor	419 (4.1%)	64 (1.0%)	193 (6.0%)	162 (17.9%)	
Smoking status					<0.001
Never	6366 (61.8%)	3559 (57.5%)	2182 (68.0%)	625 (69.1%)	
Former	805 (7.8%)	479 (7.7%)	241 (7.5%)	85 (9.4%)	
Current	3132 (30.4%)	2152 (34.8%)	785 (24.5%)	195 (21.5%)	
Drinking status					<0.001
Never	6086 (59.1%)	3372 (57.5%)	2110 (65.8%)	604 (66.7%)	
Former	815 (7.9%)	479 (7.7%)	291 (9.1%)	110 (12.2%)	
Current	3402 (33.0%)	2152 (34.8%)	807 (25.2%)	191 (21.1%)	
Hypertension					<0.001
Yes	2487 (24.1%)	1080 (17.4%)	1007 (31.4%)	400 (44.2%)	
No	7816 (75.9%)	5110 (82.6%)	2201 (68.6%)	505 (55.8%)	
Diabetes					<0.001
Yes	575 (5.6%)	215 (3.5%)	255 (7.9%)	105 (11.6%)	
No	9728 (94.4%)	5975 (96.5%)	2953 (92.1%)	800 (88.4%)	
Social work					
Yes	4817 (46.8%)	3116 (50.3%)	1367 (42.6%)	334 (36.9%)	<0.001
No	5486 (53.2%)	3074 (49.7%)	1841 (57.4%)	571 (63.1%)	
Work					<0.001
Yes	7121 (69.1%)	4599 (74.3%)	2094 (65.3%)	428 (47.3%)	
No	3182 (30.9%)	1591 (25.7%)	1114 (34.7%)	477 (52.7%)	
Sleep duration					<0.001
t ≥ 7.5 h/d	3097 (30.1%)	2100 (33.9%)	814 (25.4%)	183 (20.2%)	
t < 7.5 h/d	7206 (69.9%)	4090 (66.1%)	2394 (74.6%)	722 (79.8%)	
IADL	0.36 (0.90)	0.12 (0.44)	0.49 (0.96)	1.55 (1.63)	<0.001
ADL,	0.29 (0.85)	0.06 (0.31)	0.37 (0.87)	1.55 (1.74)	<0.001
Frailty index	13.52 (10.06)	8.78 (4.96)	17.61 (8.50)	31.60 (13.77)	<0.001
CES-D score	8.32 (6.25)	5.57 (4.35)	11.80 (6.14)	14.80 (6.73)	<0.001

Abbreviations: G1: stable and robust with no depression; G2: moderate persistent frailty and depression; G3: escalating frailty and high depression.

**Table 2:** Binary logistic models examining the relationship between frailty and depression trajectories and gastrointestinal disease or liver disease status in 2018 (CHARLS).

Characteristics	Model 1		Model 2		Model 3		Model 4	
	OR (95% CI)	*p* Value	OR (95% CI)	*p* Value	OR (95% CI)	*p* Value	OR (95% CI)	*p* Value
Gastrointestinal Disease								
G1	Reference		Reference		Reference		Reference	
G2	2.07 (1.82, 2.36)	<0.001	2.02 (1.76, 2.31)	<0.001	2.02 (1.76, 2.31)	<0.001	1.94 (1.67, 2.24)	<0.001
G3	2.78 (2.27, 3.41)	<0.001	2.79 (2.25, 3.45)	<0.001	2.80 (2.26, 3.48)	<0.001	2.73 (2.12, 3.53)	<0.001
Liver Disease								
G1	Reference		Reference		Reference		Reference	
G2	1.67 (1.38, 2.03)	<0.001	1.96 (1.60, 2.40)	<0.001	1.98 (1.62, 2.43)	<0.001	1.72 (1.38, 2.13)	<0.001
G3	2.98 (2.32, 3.83)	<0.001	3.72 (2.85, 4.86)	<0.001	3.83 (2.92, 5.03)	<0.001	3.44 (2.50, 4.75)	<0.001

**Table 3:** Association of frailty symptoms with new-onset gastrointestinal or liver diseases in the CHARLS cohort.

Characteristics	Cases	Model 1		Model 2		Model 3		Model 4	
		HR (95% CI)	*p* Value	HR (95% CI)	*p* Value	HR (95% CI)	*p* Value	HR (95% CI)	*p* Value
Gastrointestinal disease	1761								
None	669	Reference		Reference		Reference		Reference	
Pre-frailty	842	1.54 (1.39, 1.70)	<0.001	1.50 (1.35, 1.66)	<0.001	1.49 (1.35, 1.66)	<0.001	1.51 (1.35, 1.68)	<0.001
Frailty	250	2.18 (1.89, 2.52)	<0.001	2.12 (1.82, 2.46)	<0.001	2.09 (1.80, 2.44)	<0.001	2.10 (1.68, 2.62)	<0.001
Liver disease	705								
None	234	Reference		Reference		Reference		Reference	
Pre-frailty	350	1.60 (1.35, 1.89)	<0.001	1.71 (1.45, 2.03)	<0.001	1.71 (1.45, 2.03)	<0.001	1.51 (1.26, 1.81)	<0.001
Frailty	121	2.19 (1.76, 2.73)	<0.001	2.51 (1.99, 3.16)	<0.001	2.52 (2.00, 3.19)	<0.001	2.42 (1.75, 3.34)	<0.001

Model 1: Unadjusted. Model 2: Adjusted for age, gender, education, marital status, and residence. Model 3: Adjusted for age, gender, education, marital status, residence, social work, living alone, and work. Model 4: Adjusted for age, gender, education, marital status, residence, social work, living alone, work, self-rated health, smoking status, drinking status, hypertension, diabetes, sleep duration, IADL, and ADL.

**Table 4:** The relationship between the frailty index and gastrointestinal or liver disease incidence.

Characteristics	Cases	Model 1		Model 2		Model 3		Model 4	
		HR (95% CI)	*p* Value	HR (95% CI)	*p* Value	HR (95% CI)	*p* Value	HR (95% CI)	*p* Value
Gastrointestinal disease									
Frailty index (per 1 unit)	1761	1.02 (1.02, 1.03)	<0.001	1.02 (1.02, 1.03)	<0.001	1.02 (1.02, 1.03)	<0.001	1.04 (1.03, 1.05)	<0.001
Q1 (4.58 [<6.25])	349	Reference		Reference		Reference		Reference	
Q2 (7.92 [6.36–10.38])	348	1.20 (1.03, 1.39)	0.018	1.19 (1.03, 1.39)	0.019	1.20 (1.03, 1.39)	0.019	1.25 (1.07, 1.45)	0.004
Q3 (12.50 [10.49–16.52])	464	1.52 (1.32, 1.75)	<0.001	1.49 (1.29, 1.72)	<0.001	1.49 (1.29, 1.71)	<0.001	1.54 (1.33, 1.78)	<0.001
Q4 (22.94 [16.63–65.40])	600	2.03 (1.78, 2.32)	<0.001	1.97 (1.72, 2.26)	<0.001	1.96 (1.70, 2.24)	<0.001	1.89 (1.62, 2.22)	<0.001
*p* for trend		<0.001		<0.001		<0.001		<0.001	
Liver disease									
Frailty index (per 1 unit)	705	1.02 (1.01, 1.03)	<0.001	1.02 (1.02, 1.03)	<0.001	1.03 (1.02, 1.03)	<0.001	1.04 (1.03, 1.05)	<0.001
Q1 (4.58 [<6.70])	121	Reference		Reference		Reference		Reference	
Q2 (8.04 [6.80–10.83])	154	1.22 (0.96, 1.55)	0.099	1.25 (0.99, 1.59)	0.066	1.25 (0.99, 1.59)	0.065	1.20 (0.94, 1.53)	<0.001
Q3 (13.95 [10.94–17.30])	200	1.73 (1.38, 2.16)	<0.001	1.88 (1.49, 2.36)	<0.001	1.88 (1.49, 2.36)	<0.001	1.61 (1.27, 2.05)	<0.001
Q4 (23.77 [17.41–70.09])	230	1.98 (1.59, 2.47)	<0.001	2.28 (1.81, 2.87)	<0.001	2.28 (1.81, 2.88)	<0.001	1.80 (1.37, 2.36)	<0.001
*p* for trend		<0.001		<0.001		<0.001		<0.001	

Model 1: Unadjusted. Model 2: Adjusted for age, gender, education, marital status, and residence. Model 3: Adjusted for age, gender, education, marital status, residence, social work, living alone, and work. Model 4: Adjusted for age, gender, education, marital status, residence, social work, living alone, work, self-rated health, smoking status, drinking status, hypertension, diabetes, sleep duration, IADL, and ADL.

**Table 5:** Associations between depressive symptoms and new-onset gastrointestinal or liver disease incidence.

Characteristics	Cases	Model 1		Model 2		Model 3		Model 4	
		HR (95% CI)	*p* Value	HR (95% CI)	*p* Value	HR (95% CI)	*p* Value	HR (95% CI)	*p* Value
Gastrointestinal Disease	1750								
None	980	Reference		Reference		Reference		Reference	
Mild	435	1.52 (1.36, 1.70)	<0.001	1.46 (1.30, 1.64)	<0.001	1.46 (1.30, 1.63)	<0.001	1.31 (1.17, 1.48)	<0.001
Severe	335	2.20 (1.94, 2.49)	<0.001	2.05 (1.80, 2.33)	<0.001	2.03 (1.79, 2.31)	<0.001	1.66 (1.44, 1.91)	<0.001
Liver Disease	701								
None	382	Reference		Reference		Reference		Reference	
Mild	183	1.37 (1.15, 1.64)	<0.001	1.45 (1.21, 1.73)	<0.001	1.45 (1.21, 1.73)	<0.001	1.22 (1.01, 1.47)	0.034
Severe	136	1.69 (1.39, 2.05)	<0.001	1.85 (1.51, 2.27)	<0.001	1.85 (1.51, 2.27)	<0.001	1.36 (1.09, 1.70)	0.007

**Table 6:** The relationship between CESD scores and gastrointestinal or liver disease incidence.

Characteristics	Cases	Model 1		Model 2		Model 3		Model 4	
		HR (95% CI)	*p* Value	HR (95% CI)	*p* Value	HR (95% CI)	*p* Value	HR (95% CI)	*p* Value
Gastrointestinal disease									
CESD scores (per 1 unit)	1750	1.05 (1.04, 1.06)	<0.001	1.05 (1.04, 1.06)	<0.001	1.05 (1.04, 1.05)	<0.001	1.04 (1.03, 1.04)	<0.001
Q1 (2 [<3])	366	Reference		Reference		Reference		Reference	
Q2 (5 [3–6])	306	1.12 (0.97, 1.31)	0.129	1.11 (0.95, 1.29)	0.195	1.10 (0.95, 1.29)	0.200	1.07 (0.92, 1.25)	0.372
Q3 (9 [7–11])	478	1.56 (1.26, 1.78)	<0.001	1.51 (1.31, 1.73)	<0.001	1.50 (1.31, 1.72)	<0.001	1.38 (1.20, 1.58)	<0.001
Q4 (16 [12–30])	600	2.17 (1.91, 2.47)	<0.001	2.03 (1.78, 2.32)	<0.001	2.02 (1.76, 2.31)	<0.001	1.68 (1.46, 1.94)	<0.001
*p* for trend		<0.001		<0.001		<0.001		<0.001	
Liver disease									
CESD scores (per 1 unit)	701	1.04 (1.02,1.05)	<0.001	1.04 (1.03, 1.05)	<0.001	1.04 (1.03, 1.05)	<0.001	1.02 (1.01, 1.04)	<0.001
Q1 (2 [<3])	147	Reference		Reference		Reference		Reference	
Q2 (5 [3–6])	159	1.07 (0.86, 1.34)	0.532	1.12 (0.89, 1.40)	0.333	1.12 (0.89, 1.40)	0.323	1.02 (0.82, 1.29)	0.833
Q3 (10 [7–11])	175	1.32 (1.06, 1.64)	0.013	1.41 (1.13, 1.76)	0.003	1.41 (1.13, 1.76)	0.002	1.18 (0.94, 1.49)	0.150
Q4 (16 [12–30])	220	1.74 (1.41, 2.14)	<0.001	1.94 (1.56, 2.41)	<0.001	1.95 (1.57, 2.42)	<0.001	1.43 (1.12, 1.81)	0.003
*p* for trend		<0.001		<0.001		<0.001		<0.001	

**Table 7:** Binary logistic models examining the relationship between frailty and depression trajectories and gastrointestinal disease status in 2017 (SHARE).

Variables	Model 1		Model 2		Model 3		Model 4	
	OR (95% CI)	*p*	OR (95% CI)	*p*	OR (95% CI)	*p*	OR (95% CI)	*p*
Trajectories of frailty and depression (ref: G1)								
G2	2.393 (1.743, 3.286)	<0.001	2.464 (1.764, 3.442)	<0.001	2.316 (1.654, 3.243)	<0.001	2.035 (1.359, 3.048)	<0.001
G3	5.032 (3.428, 7.388)	<0.001	5.331 (3.473, 8.184)	<0.001	4.949 (3.222, 7.601)	<0.001	4.588 (2.561, 8.218)	<0.001
Age			0.990 (0.972, 1.008)	0.283	0.978 (0.958, 0.999)	0.044	0.982 (0.958, 1.006)	0.135
BMI			1.008 (0.978, 1.039)	0.626	1.006 (0.975, 1.038)	0.696	0.997 (0.961, 1.034)	0.871
Gender (ref: Female)								
Male			1.110 (0.828, 1.487)	0.485	1.137 (0.846, 1.527)	0.394	1.129 (0.801, 1.592)	0.488
Education (ref: Below high school)								
High school			0.990 (0.716, 1.371)	0.954	1.028 (0.742, 1.424)	0.867	1.010 (0.709, 1.439)	0.957
Above high school			0.829 (0.561, 1.224)	0.345	0.889 (0.601, 1.317)	0.558	0.698 (0.442, 1.102)	0.123
Marital status (ref: Others)								
Married			1.133 (0.813, 1.580)	0.460	0.992 (0.612, 1.609)	0.974	0.909 (0.538, 1.535)	0.721
Residence (ref: Urban)								
Rural			1.180 (0.878, 1.586)	0.273	1.175 (0.874, 1.580)	0.287	1.096 (0.791, 1.520)	0.581
Living alone (ref: No)								
Yes					0.850 (0.497, 1.455)	0.553	0.799 (0.444, 1.437)	0.453
Work (ref: No)								
Yes					0.610 (0.415, 0.896)	0.012	0.639 (0.420, 0.972)	0.037
Self-rated health (ref: Poor)								
Fair							0.884 (0.470, 1.662)	0.702
Good							0.594 (0.303, 1.166)	0.130
Nice							0.702 (0.333, 1.477)	0.351
Excellent							0.264 (0.086, 0.807)	0.019
Smoking status (ref: Never)								
Former							0.966 (0.673, 1.386)	0.851
Current							0.906 (0.581, 1.414)	0.663
Drinking status (ref: Never)								
Former							0.510 (0.058, 4.514)	0.545
Current							0.528 (0.060, 4.657)	0.565
Hypertension (ref: No)								
Yes							0.912 (0.571, 1.456)	0.577
Diabetes (ref: No)								
Yes							0.909 (0.650, 1.456)	0.698
IADL							0.677 (0.445, 1.030)	0.069
ADL							1.021 (0.744, 1.402)	0.896

Note. ADL: activities of daily life; IADL: instrumental activity of daily life.

## Data Availability

The data presented in this study are openly available in CHARLS at https://charls.pku.edu.cn/ and SHARE at https://share-eric.eu/.

## Supplementary Materials

The following supporting information can be downloaded at: https://www.mdpi.com/article/10.3390/healthcare14131896/s1.

## References

[1] Sperber A.D., Bangdiwala S.I., Drossman D.A., Ghoshal U.C., Simren M., Tack J., Whitehead W.E., Dumitrascu D.L., Fang X., Fukudo S. (2021). Worldwide Prevalence and Burden of Functional Gastrointestinal Disorders, Results of Rome Foundation Global Study. Gastroenterology.

[2] Tang X., Wang P., Huang S., Peng J., Zhang W., Shi X., Shi L., Zhong X., Lyu M., Zhou X. (2024). Trend of Gastrointestinal and Liver Diseases in China: Results of the Global Burden of Disease Study, 2019. Chin. Med. J..

[3] Wang Y., Liu M., Yang F., Chen H., Wang Y., Liu J. (2024). The Associations of Socioeconomic Status, Social Activities, and Loneliness with Depressive Symptoms in Adults Aged 50 Years and Older across 24 Countries: Findings from Five Prospective Cohort Studies. Lancet Healthy Longev..

[4] Hernández-Évole H., Jiménez-Esquivel N., Pose E., Bataller R. (2024). Alcohol-Associated Liver Disease: Epidemiology and Management. Ann. Hepatol..

[5] Shay J.E.S., Singh A. (2023). The Effect of Obesity on Gastrointestinal Disease. Gastroenterol. Clin. N. Am..

[6] Wu Y., Li Y., Giovannucci E. (2021). Potential Impact of Time Trend of Lifestyle Risk Factors on Burden of Major Gastrointestinal Cancers in China. Gastroenterology.

[7] Agus A., Planchais J., Sokol H. (2018). Gut Microbiota Regulation of Tryptophan Metabolism in Health and Disease. Cell Host Microbe.

[8] Bisgaard T.H., Allin K.H., Keefer L., Ananthakrishnan A.N., Jess T. (2022). Depression and Anxiety in Inflammatory Bowel Disease: Epidemiology, Mechanisms and Treatment. Nat. Rev. Gastroenterol. Hepatol..

[9] Christensen C., Knudsen A., Arnesen E.K., Hatlebakk J.G., Sletten I.S., Fadnes L.T. (2024). Diet, Food, and Nutritional Exposures and Inflammatory Bowel Disease or Progression of Disease: An Umbrella Review. Adv. Nutr..

[10] Veronese N., Pilotto A. (2024). The Importance of Multidimensional Frailty in Clinical Practice. J. Clin. Med..

[11] Hoogendijk E.O., Afilalo J., Ensrud K.E., Kowal P., Onder G., Fried L.P. (2019). Frailty: Implications for Clinical Practice and Public Health. Lancet.

[12] Asrani S.K., Devarbhavi H., Eaton J., Kamath P.S. (2019). Burden of Liver Diseases in the World. J. Hepatol..

[13] Zhong Q., Zhou R., Huang Y.-N., Huang R.-D., Li F.-R., Chen H.-W., Wei Y.-F., Liu K., Cao B.-F., Liao K.-Y. (2025). Frailty and Risk of Metabolic Dysfunction-Associated Steatotic Liver Disease and Other Chronic Liver Diseases. J. Hepatol..

[14] Wu S., Yang Z., Liu S., Zhang Q., Zhang S., Zhu S. (2023). Frailty Status and Risk of Irritable Bowel Syndrome in Middle-Aged and Older Adults: A Large-Scale Prospective Cohort Study. eClinicalMedicine.

[15] Fan J., Yu C., Guo Y., Bian Z., Sun Z., Yang L., Chen Y., Du H., Li Z., Lei Y. (2020). Frailty Index and All-Cause and Cause-Specific Mortality in Chinese Adults: A Prospective Cohort Study. Lancet Public Health.

[16] Rohrmann S., Veronese N. (2020). Epidemiology of Frailty in Older People. Frailty and Cardiovascular Diseases.

[17] Rakel R.E. (1999). Depression. Prim. Care Clin. Off. Pract..

[18] Ghia J.-E., Blennerhassett P., Collins S.M. (2008). Impaired Parasympathetic Function Increases Susceptibility to Inflammatory Bowel Disease in a Mouse Model of Depression. J. Clin. Investig..

[19] Youssef N.A., Abdelmalek M.F., Binks M., Guy C.D., Omenetti A., Smith A.D., Diehl A.M.E., Suzuki A. (2013). Associations of Depression, Anxiety and Antidepressants with Histological Severity of Nonalcoholic Fatty Liver Disease. Liver Int..

[20] Koutny F., Frey V., Datz C., Gensluckner S., Prosenz J., Langthaler P., Maieron A., Flamm M., Weghuber D., Iglseder B. (2026). Association between Depression and Metabolic Dysfunction-Associated Steatotic Liver Disease: A Cross-Sectional Analysis from the Paracelsus 10,000 Study. Hepatol. Int..

[21] Söderquist F., Syk M., Just D., Kurbalija Novicic Z., Rasmusson A.J., Hellström P.M., Ramklint M., Cunningham J.L. (2020). A Cross-Sectional Study of Gastrointestinal Symptoms, Depressive Symptoms and Trait Anxiety in Young Adults. BMC Psychiatry.

[22] Sun Y., Li X., Liu H., Li Y., Gui J., Zhang X., Li X., Sun L., Wang C., Li J. (2024). Predictive Role of Depressive Symptoms on Frailty and Its Components in Chinese Middle-Aged and Older Adults: A Longitudinal Analysis. BMC Public Health.

[23] Shao L., Zhu X., Li D.-L., Wu L., Lu X., Fan Y., Qiao Z., Hou L., Pan C.-W., Ke C. (2024). Quantifying Depressive Symptoms on Incidence of Common Chronic Diseases and Multimorbidity Patterns in Middle-Aged and Elderly Chinese Adults. J. Psychiatr. Res..

[24] Qian F., Zheng G., Chen Z. (2026). Bidirectional Causal Association between Frailty and Six Psychiatric Disorders. Eur. Arch. Psychiatry Clin. Neurosci..

[25] Soysal P., Veronese N., Thompson T., Kahl K.G., Fernandes B.S., Prina A.M., Solmi M., Schofield P., Koyanagi A., Tseng P.-T. (2017). Relationship between Depression and Frailty in Older Adults: A Systematic Review and Meta-Analysis. Ageing Res. Rev..

[26] Álvarez-Bustos A., Carnicero-Carreño J.A., Sanchez-Sanchez J.L., Garcia-Garcia F.J., Alonso-Bouzón C., Rodríguez-Mañas L. (2022). Associations between Frailty Trajectories and Frailty Status and Adverse Outcomes in Community-dwelling Older Adults. J. Cachexia Sarcopenia Muscle.

[27] Jang A.R., Sagong H., Yoon J.Y. (2022). Frailty Trajectory among Community-Dwelling Middle-Aged and Older Adults in Korea: Evidence from the Korean Longitudinal Study of Aging. BMC Geriatr..

[28] Xiang S., Li Y., Li Y., Xie S., Wang C., Che X., Du Y. (2026). Associations of Phenotypic Age Acceleration, Genetic Risk, and Lifestyle with Chronic Digestive Diseases: A Large-Scale Longitudinal Cohort Study. J. Nutr. Health Aging.

[29] Guo J., Su M., Huang J., Wang X., Li J., Wu H., He Y. (2025). Longitudinal Bidirectional Association between Gastrointestinal Disease and Depression Symptoms among Middle-Aged and Older Adults in China. Arch. Public Health.

[30] Zhou J., Chen H., Lin C. (2024). Frailty in the Elderly Is Associated with an Increased Risk of Depression: A Systematic Review and Meta-Analysis. Alpha Psychiatry.

[31] Chen D., Zhang Y., Huang T., Jia J. (2023). Depression and Risk of Gastrointestinal Disorders: A Comprehensive Two-Sample Mendelian Randomization Study of European Ancestry. Psychol. Med..

[32] Nagin D.S., Jones B.L., Elmer J. (2024). Recent Advances in Group-Based Trajectory Modeling for Clinical Research. Annu. Rev. Clin. Psychol..

[33] Nguena Nguefack H.L., Pagé M.G., Katz J., Choinière M., Vanasse A., Dorais M., Samb O.M., Lacasse A. (2020). Trajectory Modelling Techniques Useful to Epidemiological Research: A Comparative Narrative Review of Approaches. Clin. Epidemiol..

[34] Zhao Y., Hu Y., Smith J.P., Strauss J., Yang G. (2014). Cohort Profile: The China Health and Retirement Longitudinal Study (CHARLS). Int. J. Epidemiol..

[35] Boersch-Supan M., Boersch-Supan A., Andersen-Ranberg K., Borbye-Lorenzen N., Cofferen J., Deza-Lougovski Y.I., Groh R., Holmgaard S., Horton H.M., Kerschner E. (2026). Cohort Profile Update: Survey of Health, Ageing and Retirement in Europe: Biomarker Data for Age-Related Health Conditions. medRxiv.

[36] Zeng P., Li M., Cao J., Zeng L., Jiang C., Lin F. (2024). Association of Metabolic Syndrome Severity with Frailty Progression among Chinese Middle and Old-Aged Adults: A Longitudinal Study. Cardiovasc. Diabetol..

[37] Chen H., Mui A.C. (2014). Factorial Validity of the Center for Epidemiologic Studies Depression Scale Short Form in Older Population in China. Int. Psychogeriatr..

[38] Boey K.W. (1999). Cross-Validation of a Short Form of the CES-D in Chinese Elderly. Int. J. Geriat. Psychiatry.

[39] Larraga L., Saz P., Dewey M.E., Marcos G., Lobo A. (2006). Validation of the Spanish Version of the EURO-D Scale: An Instrument for Detecting Depression in Older People. Int. J. Geriat. Psychiatry.

[40] Yan R., Hu Y., Yang J., Wang H., Wang Y., Song G. (2025). Depressive Symptoms Trajectories and Cardiovascular Disease in Chinese Middle-Aged and Older Adults: A Longitudinal Cohort Study. J. Affect. Disord..

[41] Zhang Z., Xu H., Zhang R., Yan Y., Ling X., Meng Y., Zhang X., Wang Y. (2025). Frailty and Depressive Symptoms in Relation to Cardiovascular Disease Risk in Middle-Aged and Older Adults. Nat. Commun..

[42] Jayanama K., Theou O., Godin J., Mayo A., Cahill L., Rockwood K. (2022). Relationship of Body Mass Index with Frailty and All-Cause Mortality among Middle-Aged and Older Adults. BMC Med..

[43] Gao Q., Li P., Lu Z., Ma M., Zhang N., Lu Y., Yu J. (2025). Association of Frailty and Its Trajectory with the Risk of Cancer: Evidence from the China Health and Retirement Longitudinal Study (CHARLS). BMC Public Health.

[44] Song J., Wu X., Zhang Y., Song P., Zhao Y. (2023). Association between Changes in Depressive Symptoms and Falls: The China Health and Retirement Longitudinal Study (CHARLS). J. Affect. Disord..

[45] Lang P.-O., Michel J.-P., Zekry D. (2009). Frailty Syndrome: A Transitional State in a Dynamic Process. Gerontology.

[46] Serra-Prat M., Palomera E., Clave P., Puig-Domingo M. (2009). Effect of Age and Frailty on Ghrelin and Cholecystokinin Responses to a Meal Test. Am. J. Clin. Nutr..

[47] Peterson L.W., Artis D. (2014). Intestinal Epithelial Cells: Regulators of Barrier Function and Immune Homeostasis. Nat. Rev. Immunol..

[48] Jackson M.A., Jeffery I.B., Beaumont M., Bell J.T., Clark A.G., Ley R.E., O’Toole P.W., Spector T.D., Steves C.J. (2016). Signatures of Early Frailty in the Gut Microbiota. Genome Med..

[49] Dzięgielewska-Gęsiak S., Muc-Wierzgoń M. (2023). Inflammation and Oxidative Stress in Frailty and Metabolic Syndromes—Two Sides of the Same Coin. Metabolites.

[50] Sun R., Jiao D., Yuan W., Wang H., Ren L., Fu Z., Zhang J., Yue X., Wu Z., Li C. (2026). Chronic Stress Drives Liver Cancer by Impairing the Hepatic Kynurenine Pathway and Immune Surveillance. Nat. Metab..

[51] Zhang Q., Liu S., Yuan C., Sun F., Zhu S., Guo S., Wu S., Zhang S. (2023). Frailty and Pre-Frailty with Long-Term Risk of Elderly-Onset Inflammatory Bowel Disease: A Large-Scale Prospective Cohort Study. Ann. Epidemiol..

[52] Su H., Luo Q., Wang X., Yan W. (2023). Frailty Combined with Nutritional Risk Score in Predicting Postoperative Complications of Elderly Patients with Gastrointestinal Malignancies. Asian J. Surg..

[53] Zhang F., Yan Y., Ge C. (2024). Frailty as a Predictor of Adverse Outcomes in Patients with Gastric Cancer: A Systematic Review and Meta-Analysis of 75,357 Patients. Ageing Res. Rev..

[54] Zhang H., Li L., Zhang L., Li Z., Ma Y., Huang W., Zeng R., Luo D., Wu Y., Meng M. (2025). Causal Association and Genetic Correlation of Frailty with Metabolic Dysfunction-Associated Steatotic Liver Disease: Epidemiological and Genetic Analyses. Innov. Med..

[55] Porter G., Sakowitz S., Mallick S., Vadlakonda A., Curry J., Ali K., Balian J., Benharash P. (2024). Association of Frailty with Clinical and Financial Outcomes Following Liver Transplantation. Clin. Transplant..

[56] Zheng B., Li D., Zhu D., Yang Y., Yang H., Gao Y., Li S., Wang Y., Zhang X. (2026). Association of Depression and Gastrointestinal Diseases: A Three-Stage Study. Psychol. Med..

[57] Loftus E.V., Guérin A., Yu A.P., Wu E.Q., Yang M., Chao J., Mulani P.M. (2011). Increased Risks of Developing Anxiety and Depression in Young Patients with Crohn’s Disease. Am. J. Gastroenterol..

[58] Eustis S.J., McCall M.W., Murphy E.A., Wirth M.D. (2022). Association between Gastrointestinal Symptoms and Depression in a Representative Sample of Adults in the United States: Findings from National Health and Nutrition Examination Survey (2005–2016). J. Acad. Consult.-Liaison Psychiatry.

[59] Gu Y., Zhang W., Hu Y., Chen Y., Shi J. (2022). Association between Nonalcoholic Fatty Liver Disease and Depression: A Systematic Review and Meta-Analysis of Observational Studies. J. Affect. Disord..

[60] Mohamed S., Sabki Z.A., Zainal N.Z. (2014). Depression and Psychosocial Correlates of Liver Transplant Candidates: A Systematic Review: Depression in Liver Transplant Candidates. Asia-Pac. Psychiatry.

[61] Yuan Y., Peng C., Burr J.A., Lapane K.L. (2023). Frailty, Cognitive Impairment, and Depressive Symptoms in Chinese Older Adults: An Eight-Year Multi-Trajectory Analysis. BMC Geriatr..

[62] Hu C., Sun X., Li Z., He Y., Han B., Wu Z., Liu S., Jin L. (2025). Multitrajectories of Frailty and Depression with Cognitive Function: Findings from the Health and Retirement Longitudinal Study. J. Cachexia Sarcopenia Muscle.

[63] Deng M.-G., Liu F., Liang Y., Wang K., Nie J.-Q., Liu J. (2023). Association between Frailty and Depression: A Bidirectional Mendelian Randomization Study. Sci. Adv..

[64] Fu W., Xu R., Bian P., Li X., Yang K., Wang X. (2024). Exploring the Shared Genetic Basis of Major Depressive Disorder and Frailty. J. Affect. Disord..

[65] Barlattani T., Cavatassi A., Bologna A., Socci V., Trebbi E., Malavolta M., Rossi A., Martiadis V., Tomasetti C., De Berardis D. (2025). Glymphatic System and Psychiatric Disorders: Need for a New Paradigm?. Front. Psychiatry.

[66] Jiang R., Noble S., Rosenblatt M., Dai W., Ye J., Liu S., Qi S., Calhoun V.D., Sui J., Scheinost D. (2024). The Brain Structure, Inflammatory, and Genetic Mechanisms Mediate the Association between Physical Frailty and Depression. Nat. Commun..

[67] Peng L., Li X., Qi J., Shan Y., Zhang L., Yang Z., Wu X., Agogo G.O., Liu Z., Mao G. (2025). Pre-Frailty Is Associated with Higher Risk of Gastroesophageal Reflux Disease: A Large Prospective Cohort Study. Sci. Rep..

[68] Liu F., Peng Y., Wang P., Qiao Y., Si C., Wang X., Zhang M., Chen L., Song F. (2023). Associations of Physical Frailty with Incidence and Mortality of Overall and Site-Specific Cancers: A Prospective Cohort Study from UK Biobank. Prev. Med..

[69] Li S., Li S., Duan F., Lu B. (2024). Depression and NAFLD Risk: A Meta-Analysis and Mendelian Randomization Study. J. Affect. Disord..

[70] Wang J., Meng N., Chen K., Huang X., Feng L., Yang C., Li Z., Sun X. (2024). The Relationship between Depressive Symptoms and Functional Gastrointestinal Disorders (FGIDs): The Chain Mediating Effect of Sleep Disorders and Somatic Symptom. Depress. Anxiety.

[71] Bielecky A., Smith P.M. (2014). Methods of Soliciting Self-Reported Chronic Conditions in Population Surveys: Don’t Ask, Don’t Report?. Qual. Quant..

