# Supplementary material for: Trajectories of Frailty and Depression and Their Associations with the Risk of Gastrointestinal and Liver Disease: Findings from China Health and Retirement Longitudinal Study and Validation of Survey of Health, Aging and Retirement in Europe
Source: Healthcare (Basel). 2026 Jun 30;14(13):1896. doi: 10.3390/healthcare14131896 (PMC13362453; doi:10.3390/healthcare14131896)
Supplement: Supplementary file 1 [file healthcare-14-01896-s001.zip › healthcare-4222769-supplementaryend-final.pdf]

Supplementary Materials

Supplementary Table S1. Baseline characteristics of participants included and excluded across waves 1-4 in the CHARLS cohort.

Supplementary Figure S1. Flow chart of participants selected from SHARE.

Supplementary Figure S2. The distribution of frailty index in the CHARLS cohort (wave 3).

Supplementary Table S2. Definition of frailty index and control variables.

Supplementary Figure S3. The relationship between frailty index and age stratified by gender in the CHARLS cohort (wave 3).

Supplementary Figure S4. The distribution of frailty index in the SHARE cohort (wave 3).

Supplementary Figure S5. The distribution of CESD-10 score in the CHARLS cohort (wave 3).

Supplementary Figure S6. The distribution of EURO-D score in the SHARE cohort (wave 3).

Supplementary Table S3. Group-based trajectory model fit for trajectories of frailty and depression (CHARLS).

Supplementary Table S4. The parameters of trajectories of frailty and depression (based on model 333-333) (CHARLS).

Supplementary Table S5. Odds of correct classification for trajectories of frailty and depression.

Supplementary Table S6. Baseline characteristics of participants by the status of gastrointestinal diseases in 2017(SHARE).

Supplementary Figure S7. The proportional hazards (PHs) assumption using Schoenfeld residuals test for each covariate for Cox model for the relationship between frailty and gastrointestinal disease incidence (CHARLS).

Supplementary Figure S8. The proportional hazards (PHs) assumption using Schoenfeld residuals test for each covariate for Cox model for the relationship between frailty and liver disease incidence (CHARLS).

Supplementary Figure S9. The proportional hazards (PHs) assumption using Schoenfeld residuals test for each covariate for Cox model for the relationship between frailty index and gastrointestinal disease incidence (CHARLS).

Supplementary Figure S10. The proportional hazards (PHs) assumption using Schoenfeld residuals test for each covariate for Cox model for the relationship between frailty index and liver disease incidence (CHARLS).

Supplementary Figure S11. The proportional hazards (PHs) assumption using Schoenfeld residuals test for each covariate for Cox model for the relationship between depression and gastrointestinal disease incidence (CHARLS).

Supplementary Figure S12. The proportional hazards (PHs) assumption using Schoenfeld residuals test for each covariate for Cox model for the relationship between depression and liver disease incidence (CHARLS).

Supplementary Figure S13. The proportional hazards (PHs) assumption using Schoenfeld residuals test for each covariate for Cox model for the relationship between CES-D10 score and gastrointestinal disease incidence (CHARLS).

Supplementary Figure S14. The proportional hazards (PHs) assumption using Schoenfeld residuals test for each covariate for Cox model for the relationship between CES-D10 score and liver disease incidence (CHARLS).

Supplementary Figure S15. Subgroup analysis of the associations between frail symptoms and gastrointestinal or liver disease.

Supplementary Figure S16. Subgroup analysis of the associations between frail symptoms and gastrointestinal or liver disease.

Supplementary Figure S17. Subgroup analysis of the associations between frailty and depression trajectories and gastrointestinal disease (SHARE).

Supplementary Table S7. Sensitivity analysis of frailty and depression trajectories for gastrointestinal and liver disease by discrete survival model by discrete complementary log-log regression.

Supplementary Figure S18. Trajectories of frailty and depression from wave 1 to wave 4 excluding participants with gastrointestinal disease and who had used gastrointestinal drugs (CHARLS).

Supplementary Table S8. Group-based trajectory model fit for trajectories of frailty and depression excluding participants with gastrointestinal disease and who had used gastrointestinal drugs (CHARLS).

Supplementary Table S9. The Parameters of trajectories of frailty and depression excluding participants with gastrointestinal disease and who had used gastrointestinal drugs (based on model 333-333) (CHARLS).

Supplementary Table S10. Odds of correct classification for trajectories of frailty and depression excluding participants with gastrointestinal disease and who had used gastrointestinal drugs.

Supplementary Table S11. Binary Logistic Regression models examining the relationship between frailty and depression trajectories and gastrointestinal disease status in 2018 excluding participants with gastrointestinal disease and who had used gastrointestinal

drugs.

Supplementary Figure S19. Trajectories of frailty and depression from wave 1 to wave 4 excluding participants with liver disease and who had used liver drugs (CHARLS).

Supplementary Table S12. Group-based trajectory model fit for trajectories of frailty and depression excluding participants with liver disease and who had used liver drugs (CHARLS).

Supplementary Table S13. The parameters of trajectories of frailty and depression excluding participants with liver disease and who had used liver drugs (based on model 333-333) (CHARLS).

Supplementary Table S14. Odds of correct classification for trajectories of frailty and depression excluding participants with liver disease and who had used liver drugs.

Supplementary Table S15. Binary Logistic Regression models examining the relationship between frailty and depression trajectories and liver disease status in 2018 excluding participants with liver disease and who had used liver drugs.

Supplementary Table S16. Baseline characteristics of participants by the status of gastrointestinal diseases in 2017(SHARE).

Supplementary Table S17. Group-based trajectory model fit for trajectories of frailty and depression (SHARE).

Supplementary Table S18. The parameters of trajectories of frailty and depression (based on model 332-111) (SHARE).

Supplementary Table S19. Odds of correct classification for trajectories of frailty and depression (SHARE).

Supplementary Table S20. Association of frailty and depression trajectories with new-onset gastrointestinal diseases of SHARE (discrete survival model).

Supplementary Figure S20. Trajectories of frailty and depression from wave 1 to wave 4 excluding participants with gastrointestinal disease and who had used gastrointestinal drugs (SHARE).

Supplementary Table S21. Group-based trajectory model fit for trajectories of frailty and depression excluding participants with gastrointestinal disease and who had used gastrointestinal drugs (SHARE).

Supplementary Table S22. The parameters of trajectories of frailty and depression excluding participants with gastrointestinal disease and who had used gastrointestinal drugs (based on model 312-211) (SHARE).

Supplementary Table S23. Odds of correct classification for trajectories of frailty and depression excluding participants with gastrointestinal disease and who had used gastrointestinal drugs (SHARE).

Supplementary Table S24. Binary Logistic Regression models examining the relationship between frailty and depression trajectories and gastrointestinal disease status in 2017 excluding participants with gastrointestinal disease and who had used gastrointestinal drugs (SHARE).

Supplementary Table S1. Baseline characteristics of participants included and excluded across waves 1-4 in the CHARLS cohort.

Variable	Included (10303)	Excluded (7295)	P-value
Age, years	57.561(8.733)	60.219(11.425)	<0.001
Gender, %			<0.001
Male	4732(45.9)	3735(51.2)	
Female	5571(54.1)	3558(48.8)	

Residence, %			<0.001
Urban	3580(34.7)	3547(48.6)	
Rural	6723(65.3)	3748(51.4)	
Education, %			<0.001
Below high school	9155(88.9)	6194(85.1)	
High school	789(7.7)	634(8.7)	
Above high school	359(3.5)	451(6.2)	
Marital status, %			<0.001
Married	9288(90.1)	6056(83.2)	
Other	1015(9.9)	1227(16.8)	
Smoking status, %			<0.001
Never	6366(61.8)	4154(64)	
Former	805(7.8)	611(9.4)	

Current	3132(30.4)	1729(26.6)	
Drinking status, %			0.12
Never	6086(59.1)	4156(58.3)	
Former	815(7.9)	626(8.8)	
Current	3402(33.0)	2348(32.9)	
Hypertension, %			<0.001
Yes	2487(24.1)	2033(28.8)	
No	7816(75.9)	5025(71.2)	
Diabetes, %			<0.001
Yes	575(5.6)	486(6.9)	
No	9728(94.4)	6509(93.1)	
Living alone, %			<0.001
Yes	442(4.3)	592(8.1)	

No	9861(95.7)	6703(91.9)	
Social work, %			0.98
Yes	4817(46.8)	2719(46.7)	
No	5486(53.2)	3099(53.3)	
Work, %			<0.001
Yes	7121(69.1)	3661(53.5)	
No	3182(30.9)	3185(46.5)	
Sleep duration, %			1.00
t≥7.5h/d	3097(30.1)	1709(30.1)	
t<7.5h/d	7206(69.9)	3977(69.9)	
ADL			
IADL	0.287(0.855)	0.487(1.240)	<0.001
	0.358(0.896)	0.652(1.349)	<0.001

Frailty index	13.254(10.057)	15.620(13.612)	<0.001
CES-D score	8.324(6.253)	8.544(6.467)	0.035

Supplementary Figure S1. Flow chart of participants selected from SHARE.

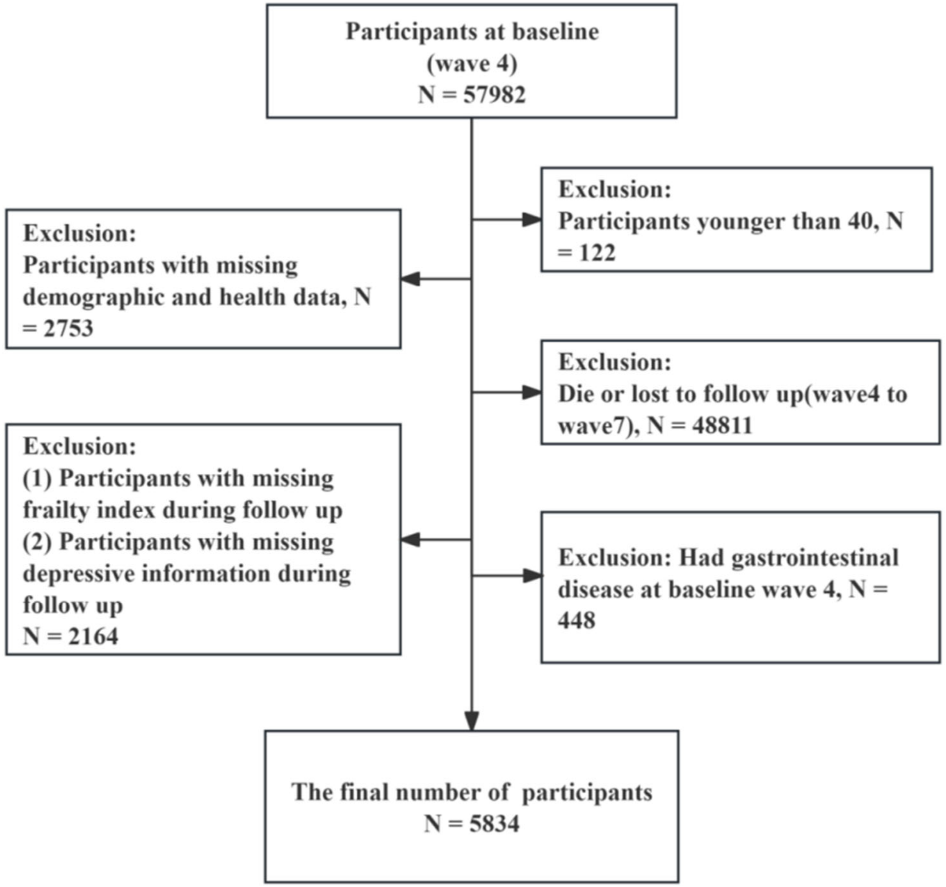

Supplementary Figure S2. The distribution of frailty index in the CHARLS cohort (wave 3).

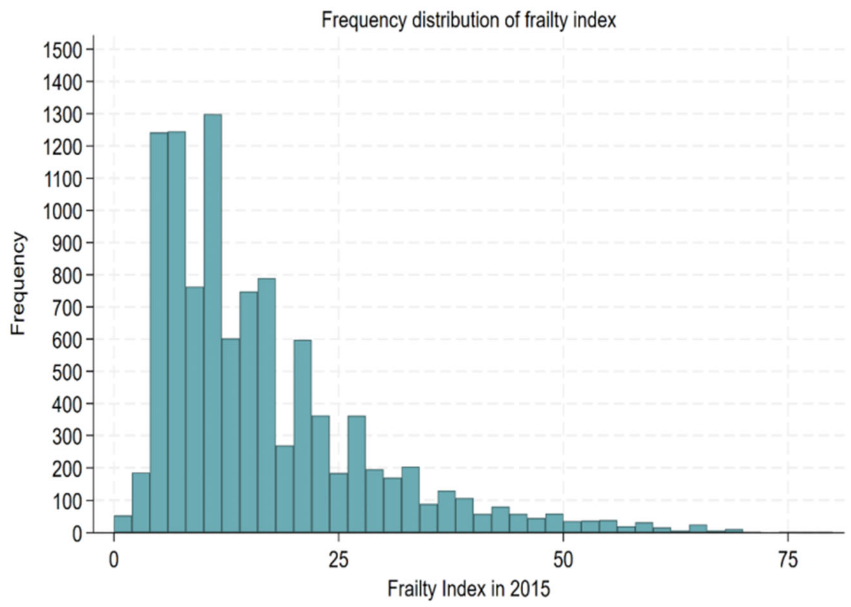

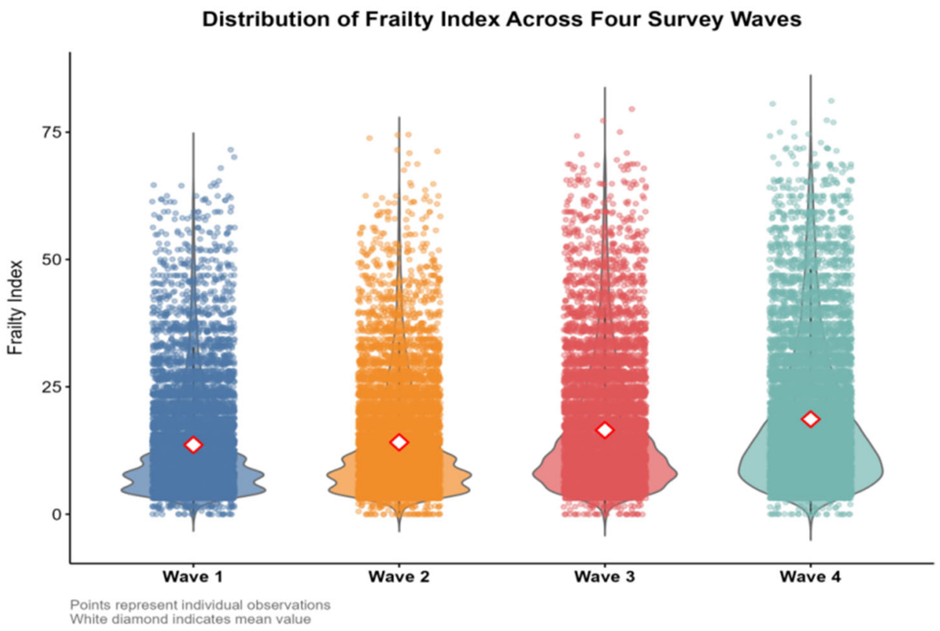

Supplementary Figure S2. The Distribution of CESD-10 score in the CHARLS cohort.

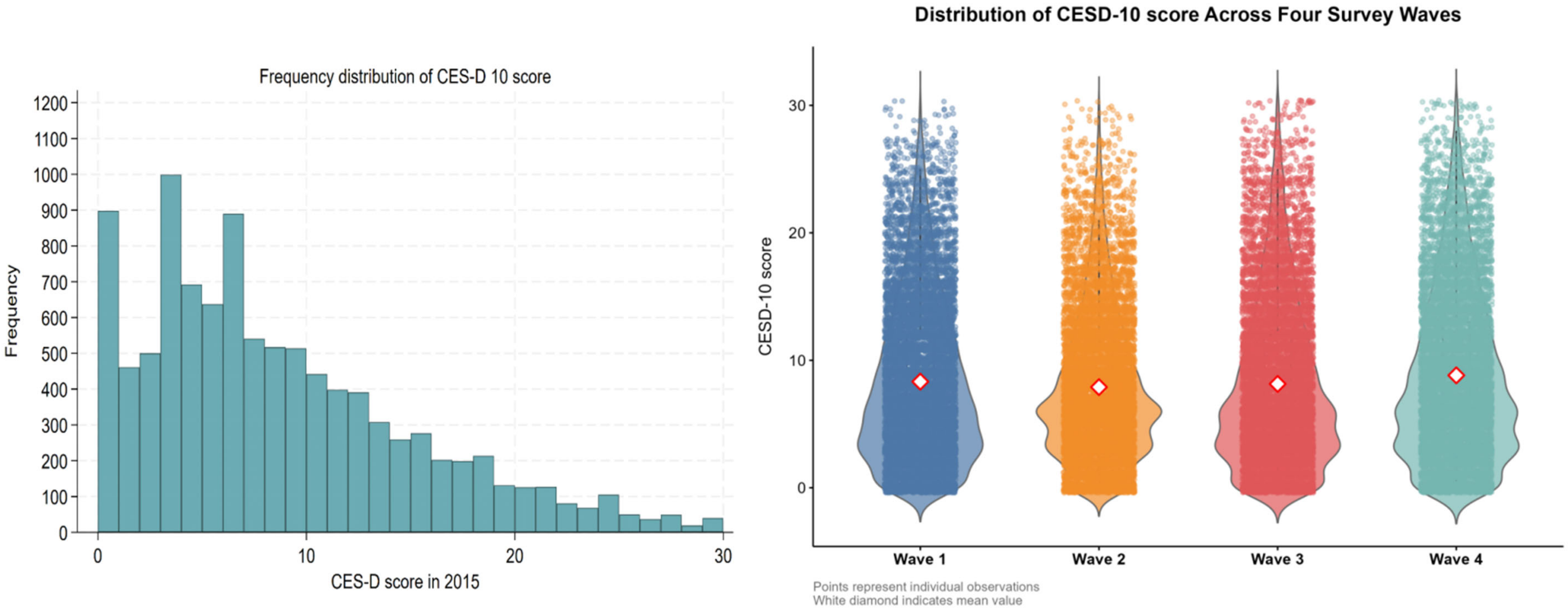

Supplementary Table S2. Definition of frailty index and control variables.

Number	Description of the items	Categorization
1	Self-reported diagnosed hypertension by doctors	Yes=1, No=0
2	Self-reported diagnosed diabetes by doctors	Yes=1, No=0
3	Self-reported diagnosed heart disease by doctors	Yes=1, No=0
4	Self-reported diagnosed stroke by doctors	Yes=1, No=0
5	Self-reported diagnosed cancer by doctors	Yes=1, No=0
6	Self-reported diagnosed arthritis by doctors	Yes=1, No=0
7	Self-reported diagnosed lung disease by doctors	Yes=1, No=0

8	Self-reported diagnosed asthma by doctors	Yes=1, No=0
9	Self-reported diagnosed emotional, nervous, or psychiatric problems by doctors	Yes=1, No=0
10	Self-reported diagnosed memory-related disease by doctors	Yes=1, No=0
11	Self-reported diagnosed vision problems	Yes=1, No=0
12	Self-reported diagnosed hearing problems	Yes=1, No=0
13	Self-rated healthy status	Poor or fair = 1, Good = 0
14	Any difficulty with dressing	Yes=1, No=0
15	Any difficulty with bathing or showering	Yes=1, No=0
16	Any difficulty with eating	Yes=1, No=0
17	Any difficulty with getting in or out of bed	Yes=1, No=0

18	Any difficulty with using the toilet	Yes=1, No=0
19	Any difficulty with managing money	Yes=1, No=0
20	Any difficulty with taking medications	Yes=1, No=0
21	Any difficulty with shopping	Yes=1, No=0
22	Any difficulty with preparing hot meals	Yes=1, No=0
23	Any difficulty with doing housework	Yes=1, No=0
24	Any difficulty with walking 100 meters	Yes=1, No=0
25	Any difficulty with getting up from a chair	Yes=1, No=0
26	Any difficulty with climbing several flights of stairs without resting	Yes=1, No=0
27	Any difficulty with lifting or carrying weights over 10 pounds	Yes=1, No=0
28	Any difficulty with picking up a small coin	Yes=1, No=0

29	Any difficulty with stooping, kneeling, or crouching	Yes=1, No=0
30	Any difficulty with reaching arms above shoulder level	Yes=1, No=0
31	Depression: CESD -10 scores	CESD-10<10=0 CESD-10≥10=1
32	Cognition: (memory score + orientation score)/14	Continuous, 0-1

	Description of the items	Categorization
1	Self-reported diagnosed hypertension by doctors	Yes=1, No=0

2	Self-reported diagnosed diabetes by doctors	Yes=1, No=0
3	Self-reported diagnosed heart disease by doctors	Yes=1, No=0
4	Self-reported diagnosed stroke by doctors	Yes=1, No=0
5	Self-reported diagnosed cancer by doctors	Yes=1, No=0
6	Self-reported diagnosed arthritis by doctors	Yes=1, No=0
7	Self-reported diagnosed lung disease by doctors	Yes=1, No=0
8	Self-reported diagnosed emotional disorders, including anxiety, nervous or psychiatric problems by doctors	Yes=1, No=0
9	Self-reported diagnosed Alzheimer's disease by doctors	Yes=1, No=0

10	Self-reported diagnosed vision problems	Yes=1, No=0
11	Self-reported diagnosed hearing problems	Yes=1, No=0
12	Self-rated healthy status	Poor = 1, Fair = 0.75, Good = 0.5, Nice =0.25, Excellent = 0
13	Any difficulty with dressing	Yes=1, No=0
14	Any difficulty with bathing or showering	Yes=1, No=0
15	Any difficulty with eating	Yes=1, No=0
16	Any difficulty with getting in or out of bed	Yes=1, No=0
17	Any difficulty with using the toilet	Yes=1, No=0
18	Any difficulty with managing money	Yes=1, No=0
19	Any difficulty with taking medications	Yes=1, No=0

20	Any difficulty with shopping	Yes=1, No=0
21	Any difficulty with preparing hot meals	Yes=1, No=0
22	Any difficulty with doing housework	Yes=1, No=0
23	Any difficulty with walking 100 meters	Yes=1, No=0
24	Any difficulty with getting up from a chair	Yes=1, No=0
25	Any difficulty with climbing several flights of stairs without resting	Yes=1, No=0
26	Any difficulty with picking a small coin from a table	Yes=1, No=0
27	Any difficulty with stooping, kneeling, or crouching	Yes=1, No=0
28	Any difficulty with reaching arms above shoulder level	Yes=1, No=0

29	Depression: EURO-D scores	EURO-D<4=0<br>EURO-D≥4=1
30	Cognition	Continuous, 0-1

Supplementary Figure S3. The relationship between frailty index and age stratified by gender in the CHARLS cohort (wave 3).

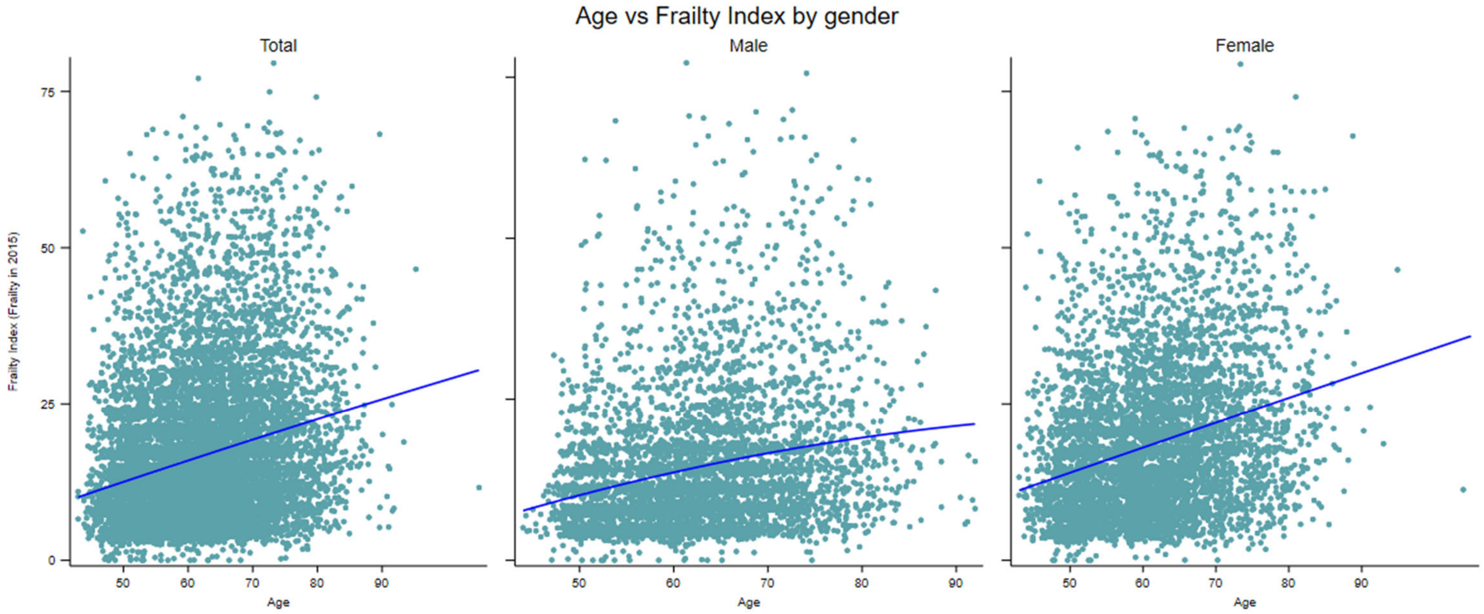

Note. The frailty index and CESD-10 score both showed a right-skewed distribution. The frailty index showed a non-linear relationship with age, with a significant Spearman correlation of 0.2640 ($p<0.001$). Females had evidently higher frailty index scores than males, with means of 18.33(SD=12.40) vs. 14.13(SD=10.70).

Supplementary Figure S4. The distribution of frailty index in the SHARE cohort (wave 3).

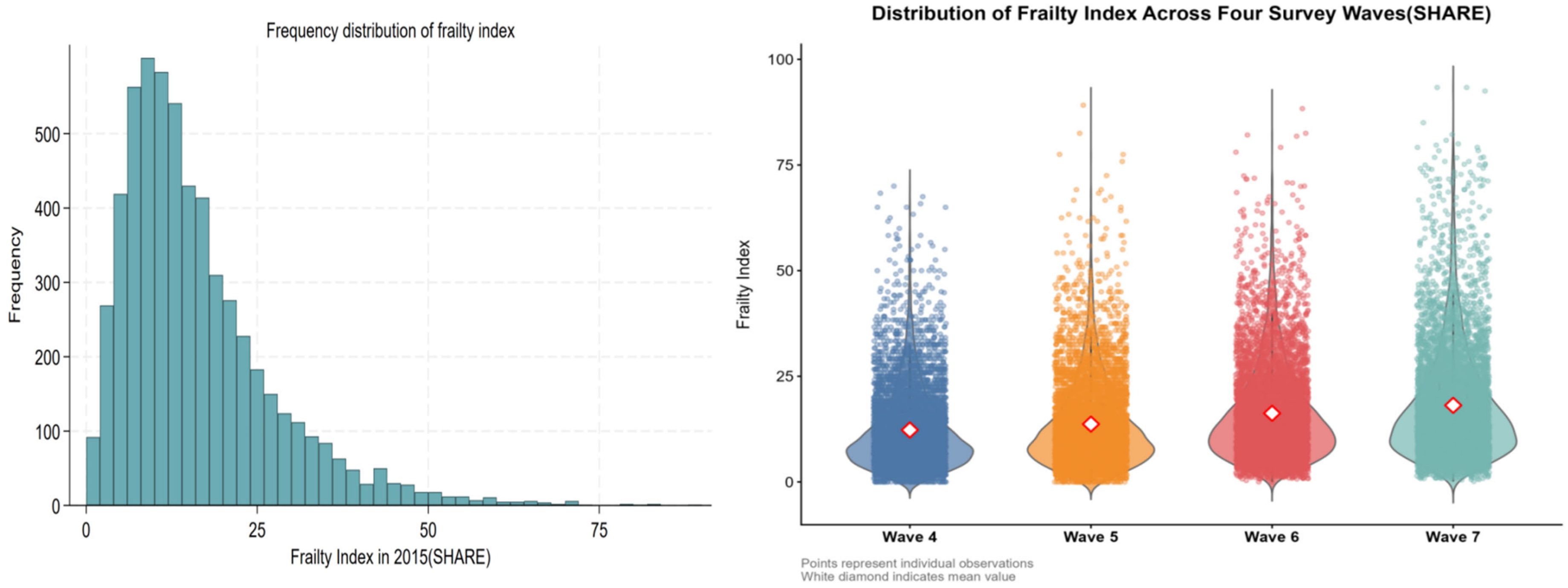

Supplementary Figure S5. The distribution of CESD-10 score in the CHARLS cohort (wave 3).

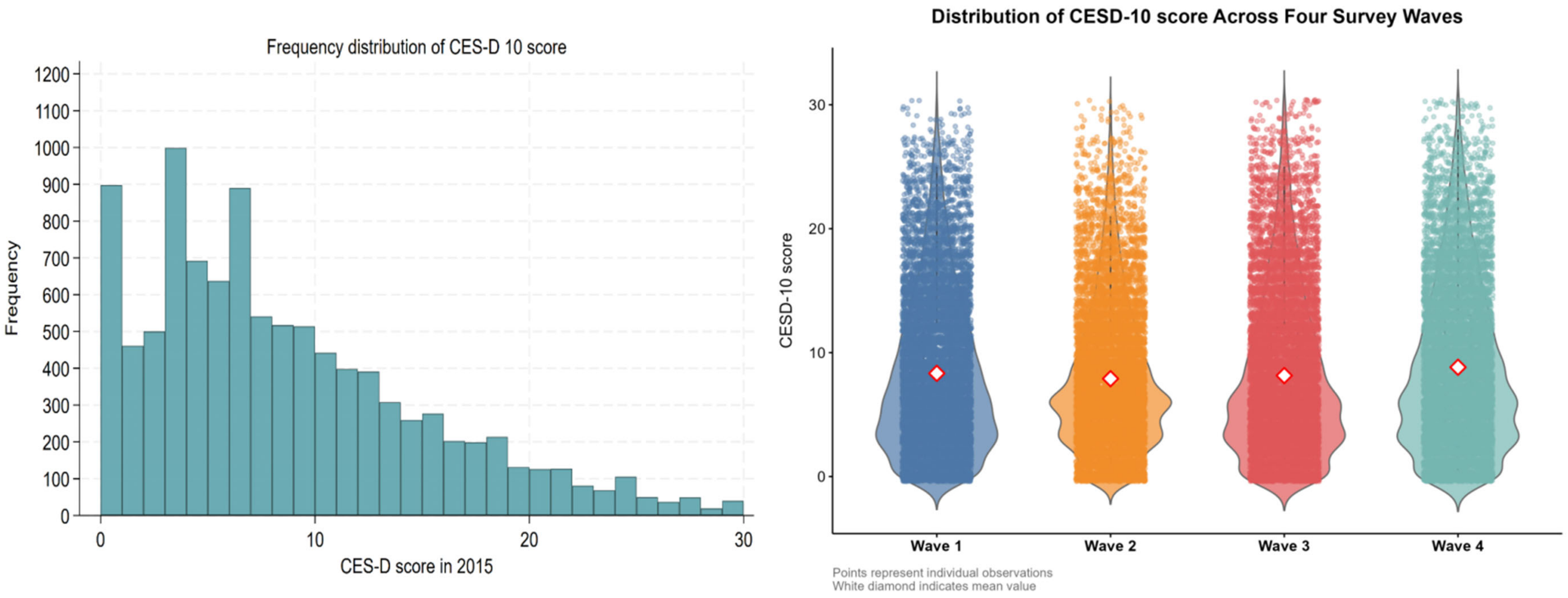

Supplementary Figure S6. The distribution of EURO-D score in the SHARE cohort (wave 3).

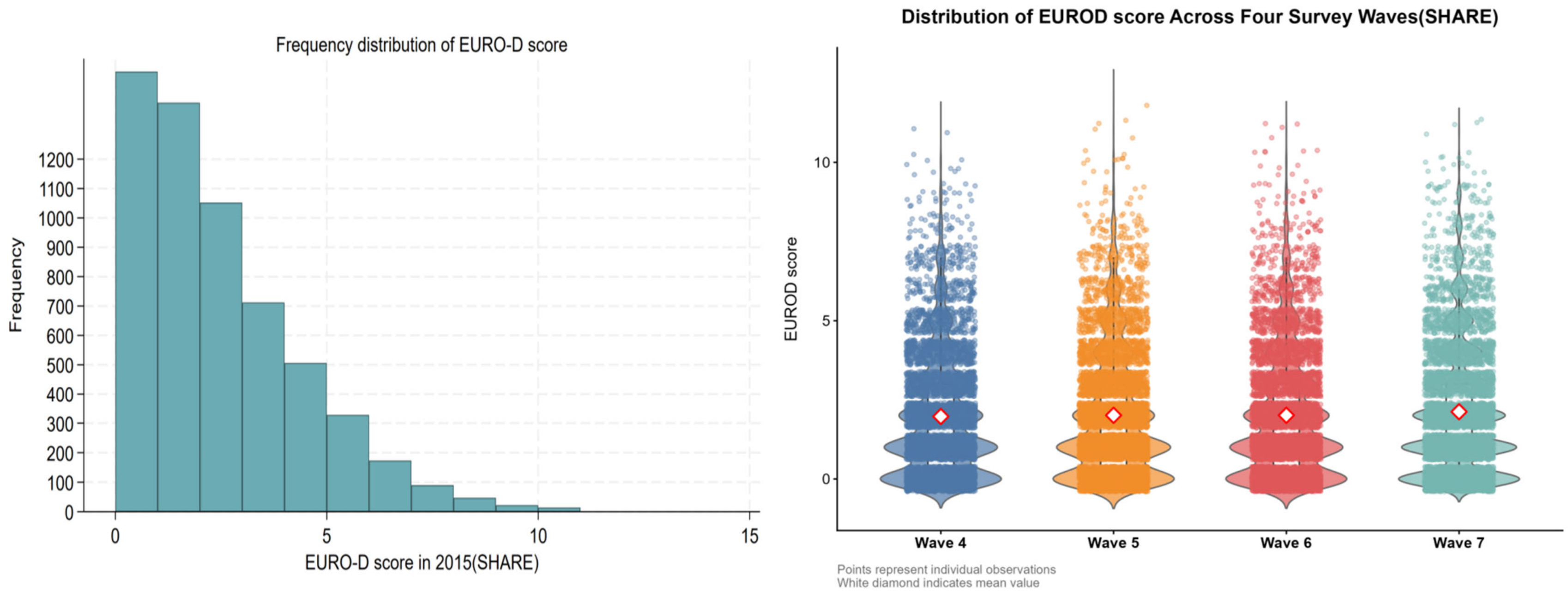

Supplementary Table S3. Group-based trajectory model fit for trajectories of frailty and depression (CHARLS).

Note. AvepP: Average Predicted Probability.

Number of classes	Parameters of trajectory shape	Proportions per class%	AvepP	AIC	BIC	ll	Entropy
2	33-33	24.98-75.02	0.949-0.980	-271051.08	-271070.66	-270963.30	0.906
3	333-333	8.83-59.65	0.918-0.958	-267011.59	-267141.80	-266983.59	0.880
4	3333-3333	3.33-52.56	0.876-0.913	-265554.01	-265726.07	-265517.01	0.859
5 Best	33333-33333	4.19-49.23	0.844-0.944	-264550.89	-264764.80	-264504.89	0.841

fit							
3	333-333	8.83-59.65	0.918-0.958	-267011.59	-267141.80	-266983.59	0.880

Supplementary Table S4. The parameters of trajectories of frailty and depression (based on model 333-333) (CHARLS).

**Frailty-Depression**	**Trajectory-Group**	**Parameter**	**Estimate**	**Standard error**	**T value**	**P value**

Frailty	G1	Intercept	8.77422	0.10192	86.087	<0.001
		Linear	-0.30120	0.17198	-1.751	0.0799
		Quadratic	0.22927	0.06898	3.324	0.0009
		Cubic	-0.01920	0.00665	-2.887	0.0039
	G2	Intercept	17.47536	0.16012	109.137	<0.001
		Linear	-0.75424	0.24696	-3.054	0.0023
		Quadratic	0.67139	0.09899	6.782	<0.001
		Cubic	-0.05873	0.00954	-6.158	<0.001
	G3	Intercept	31.52409	0.31188	101.077	<0.001
		Linear	-1.44828	0.47465	-3.051	0.0023
		Quadratic	1.65773	0.19081	8.688	<0.001
		Cubic	-0.16443	0.01843	-8.923	<0.001
Depression	G1	Intercept	5.28750	0.07860	67.269	<0.001

	Linear	0.14609	0.13033	1.121	0.2623
	Quadratic	-0.13554	0.05225	-2.594	0.0095
	Cubic	0.01730	0.00503	3.435	0.0006
G2	Intercept	11.63373	0.11176	104.093	<0.001
	Linear	-1.09659	0.18201	-6.025	<0.001
	Quadratic	0.39089	0.07286	5.365	<0.001
	Cubic	-0.03096	0.00702	-4.409	<0.001
G3	Intercept	14.73439	0.20030	73.562	<0.001
	Linear	-1.80719	0.33775	-5.351	<0.001
	Quadratic	0.81573	0.13543	6.023	<0.001
	Cubic	-0.07644	0.01307	-5.847	<0.001

Abbreviations: G1: stable and robust with no depression; G2: moderate persistent frailty and depression; G3: escalating frailty and high depression

Supplementary Table S5. Odds of correct classification for trajectories of frailty and depression.

Trajectory group	OCC	OCCw
G1	15.3400	15.6161
G2	24.6989	24.2596
G3	191.9743	190.9626

Note: G1: stable and robust with no depression; G2: moderate persistent frailty and depression; G3: escalating frailty and high depression

OCC: Odds of correct classification;

OCCw: Odds of correct classification using weighted posterior probabilities.

Supplementary Table S6. Baseline characteristics of participants by the status of gastrointestinal diseases in 2017(SHARE).

Variable	Gastrointestinal Diseases in 2017			
	Total	Not had	Had	P
	(N = 5834)	(N = 5630, 96.5%)	(N = 204, 3.5%)	
**Trajectories of frailty and depression, %**				<0.001
G1	3591(61.6)	3517(62.5)	74(36.3)	
G2	1773(30.4)	1688(30.0)	85(41.7)	
G3	470(8.1)	425(7.5)	45(22.1)	
**Age, M±SD**	60.45(8.00)	60.42(8.00)	61.35(7.75)	0.104
**BMI**	26.48 (4.32)	26.45 (4.30)	27.27 (4.89)	0.008

Variable	Gastrointestinal Diseases in 2017			
	Total (N = 5834)	Not had (N = 5630, 96.5%)	Had (N = 204, 3.5%)	P
**Gender, %**				0.846
Male	3021(51.8)	2914(51.8)	107(52.5)	
Female	2813(48.2)	2716(48.2)	97(47.5)	
**Education, %**				0.037
Below high school	2131(36.5)	2042(36.3)	89(43.6)	
High school	2100(36.0)	2027(36.0)	73(35.8)	
Above high school	1603(27.5)	1561(27.7)	42(20.6)	
**Marital status, %**				0.982
Married	4343(74.4)	4191(74.4)	152(74.5)	
Other	1491(25.6)	1439(25.6)	52(25.5)	

Variable	Gastrointestinal Diseases in 2017			
	Total	Not had	Had	P
	(N = 5834)	(N = 5630, 96.5%)	(N = 204, 3.5%)	
**Residence, %**				0.136
Urban	3968(68.0)	3839(68.2)	129(63.2)	
Rural	1866(32.0)	1791(31.8)	75(36.8)	
**Living alone, %**				0.711
Yes	1204(20.6)	1164(20.7)	40(19.6)	
No	4630(79.4)	4466(79.3)	164(80.4)	
**Self-rated health, %**				<0.001
Poor	225(3.9)	207(3.7)	18(8.8)	
Fair	1181(20.2)	1108(19.7)	73(35.8)	
Good	2352(40.3)	2283(40.6)	69(33.8)	

Variable	Gastrointestinal Diseases in 2017			
	Total	Not had	Had	P
	(N = 5834)	(N = 5630, 96.5%)	(N = 204, 3.5%)	
Nice	1453(24.9)	1415(25.1)	38(18.6)	
Excellent	623(10.7)	617(11.0)	6(2.9)	
**Smoking status, %**				0.825
Never	2883(49.4)	2778(49.3)	105(51.5)	
Former	1931(33.1)	1867(33.2)	64(31.4)	
Current	1020(17.5)	985(17.5)	35(17.2)	
**Drinking status, %**				0.357
Never	8(0.1)	1(0.5)	7(0.1)	
Former	1570(26.9)	57(27.9)	1513(26.9)	
Current	4256(73.0)	146(71.6)	4110(73.0)	

Variable	Gastrointestinal Diseases in 2017			
	Total	Not had	Had	P
	(N = 5834)	(N = 5630, 96.5%)	(N = 204, 3.5%)	
**Hypertension, %**				0.012
Yes	2532(43.4)	106(52.0)	2426(43.1)	
No	3302(56.6)	98(48.0)	3204(56.9)	
**Diabetes, %**				0.014
Yes	612(10.5)	32(15.7)	580(10.3)	
No	5222(89.5)	172(84.3)	5050(89.7)	
**Work, %**				<0.001
Yes	2256(38.7)	51(25.0)	2205(39.2)	
No	3578(61.3)	153(75.0)	3425(60.8)	
**IADL, M±SD**	0.10(0.44)	0.13(0.42)	0.10(0.44)	0.240

Variable	Gastrointestinal Diseases in 2017			
	Total (N = 5834)	Not had (N = 5630, 96.5%)	Had (N = 204, 3.5%)	P
**ADL, M±SD**	0.14(0.48)	0.25(0.61)	0.13(0.48)	0.001

Supplementary Figure S7. The proportional hazards (PHs) assumption using Schoenfeld residuals test for each covariate for Cox model for the relationship between frailty and gastrointestinal disease incidence (CHARLS).

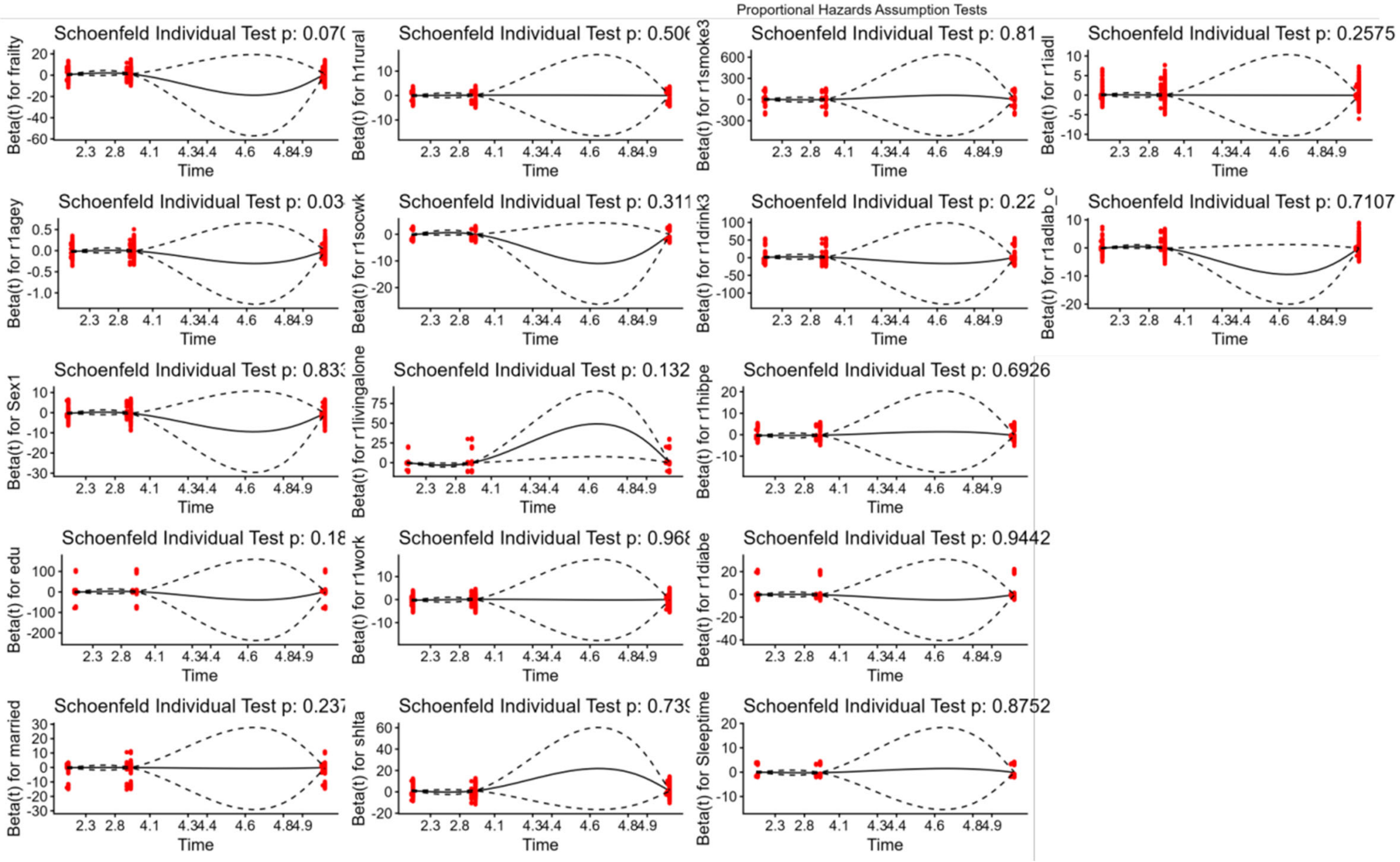
Proportional Hazards Assumption Tests
Schoenfeld Individual Test p: 0.070
Beta(t) for frailty
Schoenfeld Individual Test p: 0.506
Beta(t) for h1rural
Schoenfeld Individual Test p: 0.81
Beta(t) for r1smoke3
Schoenfeld Individual Test p: 0.2575
Beta(t) for r1iadl
Schoenfeld Individual Test p: 0.03
Beta(t) for r1agey
Schoenfeld Individual Test p: 0.311
Beta(t) for r1socwk
Schoenfeld Individual Test p: 0.22
Beta(t) for r1drink3
Schoenfeld Individual Test p: 0.7107
Beta(t) for r1adlab_c
Schoenfeld Individual Test p: 0.833
Beta(t) for Sex1
Schoenfeld Individual Test p: 0.132
Beta(t) for r1livingalone
Schoenfeld Individual Test p: 0.6926
Beta(t) for r1hibpe
Schoenfeld Individual Test p: 0.18
Beta(t) for edu
Schoenfeld Individual Test p: 0.968
Beta(t) for r1work
Schoenfeld Individual Test p: 0.9442
Beta(t) for r1diabe
Schoenfeld Individual Test p: 0.237
Beta(t) for married
Schoenfeld Individual Test p: 0.739
Beta(t) for shlta
Schoenfeld Individual Test p: 0.8752
Beta(t) for Sleeptime
Time

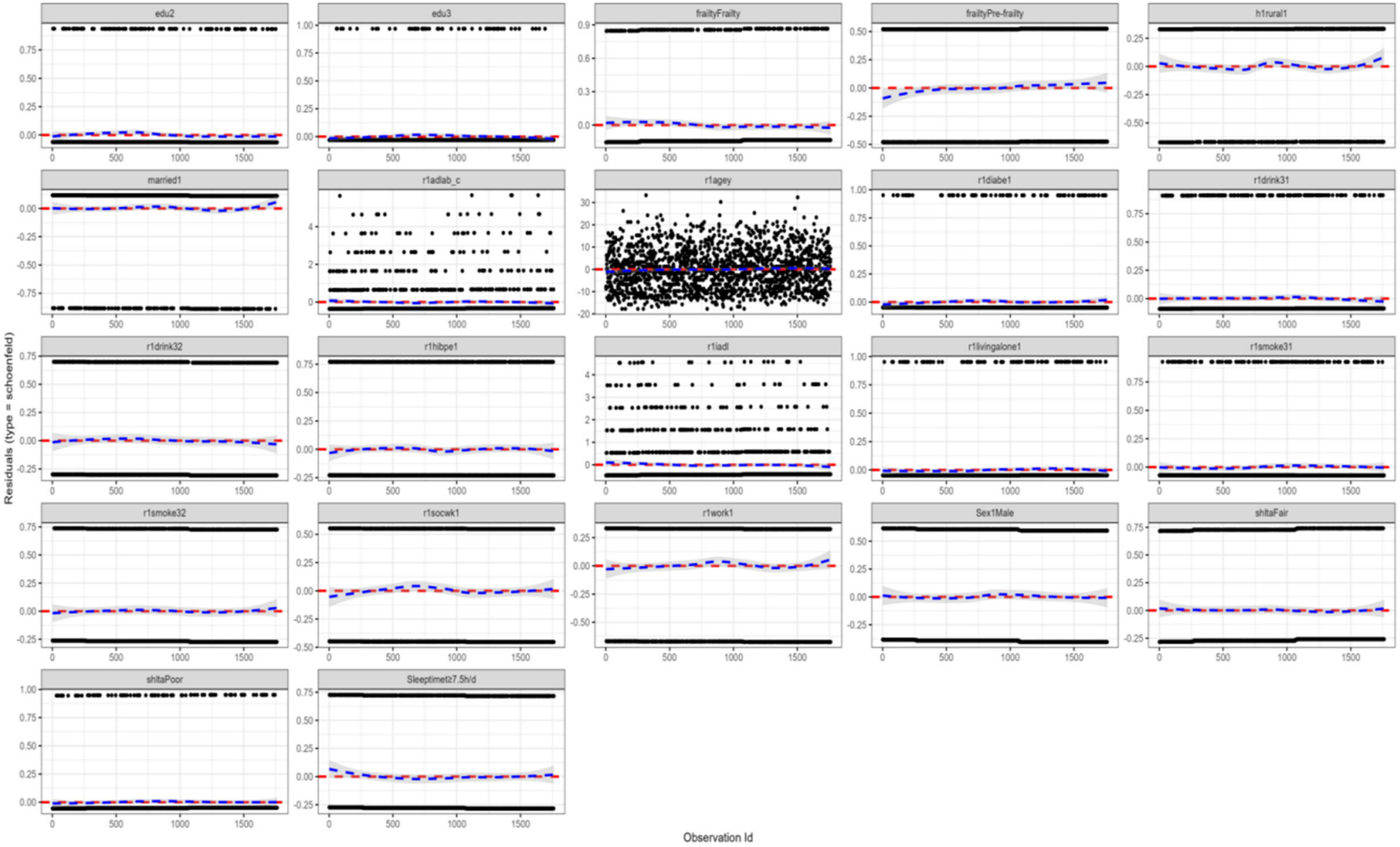
edu2
edu3
frailtyFrailty
frailtyPre-frailty
h1rural1
married1
r1adlab_c
r1agey
r1diabe1
r1drink31
r1drink32
r1hibpe1
r1iadl
r1livingalone1
r1smoke31
r1smoke32
r1socwk1
r1work1
Sex1Male
shltaFair
shltaPoor
Sleeptime≥7.5h/d
Residuals (type = schoenfeld)
Observation Id

Supplementary Figure S8. The proportional hazards (PHs) assumption using Schoenfeld residuals test for each covariate for Cox model for the relationship between frailty and liver disease incidence (CHARLS).

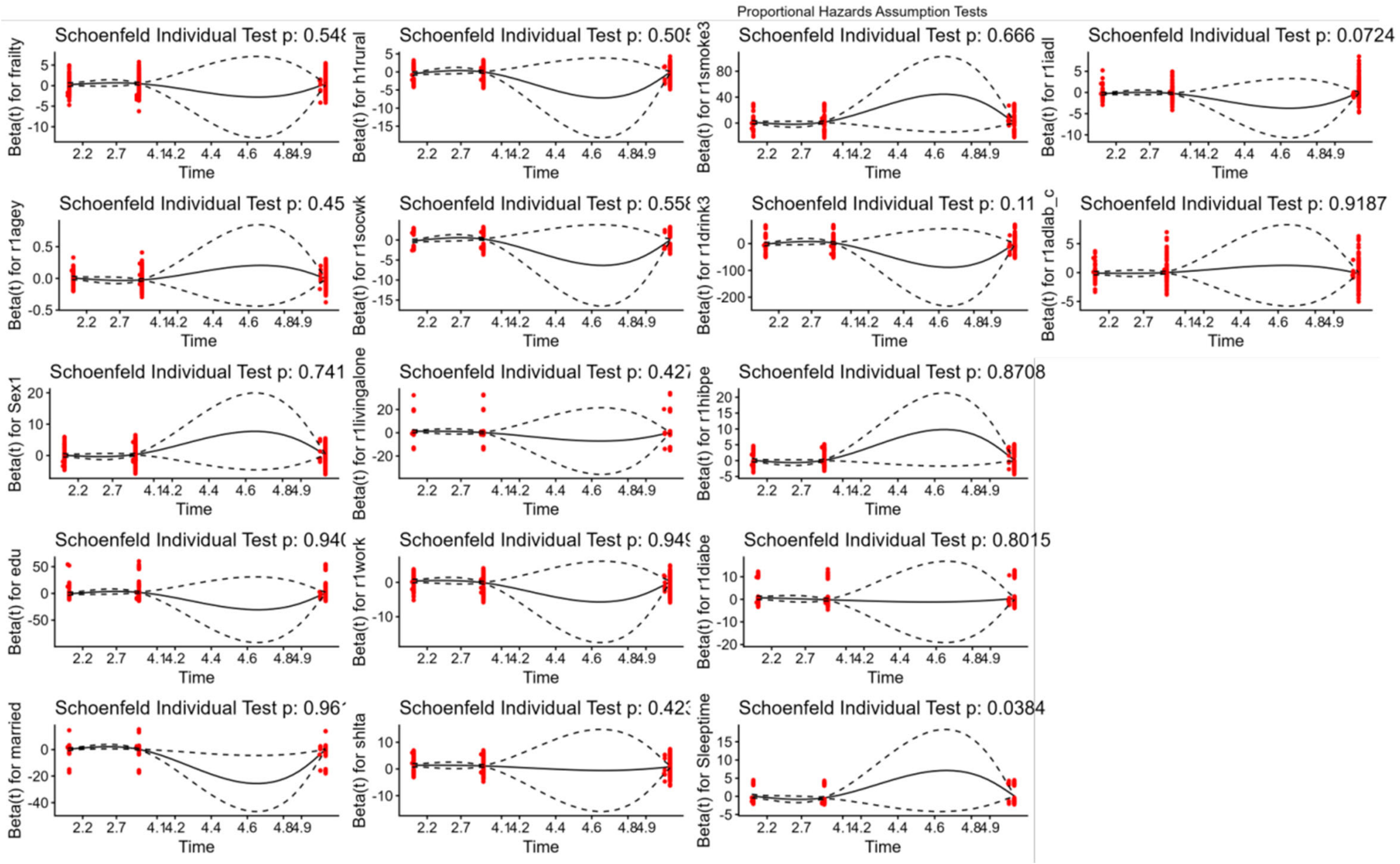
Proportional Hazards Assumption Tests
Schoenfeld Individual Test p: 0.548
Schoenfeld Individual Test p: 0.505
Schoenfeld Individual Test p: 0.666
Schoenfeld Individual Test p: 0.0724
Schoenfeld Individual Test p: 0.45
Schoenfeld Individual Test p: 0.558
Schoenfeld Individual Test p: 0.11
Schoenfeld Individual Test p: 0.9187
Schoenfeld Individual Test p: 0.741
Schoenfeld Individual Test p: 0.427
Schoenfeld Individual Test p: 0.8708
Schoenfeld Individual Test p: 0.940
Schoenfeld Individual Test p: 0.949
Schoenfeld Individual Test p: 0.8015
Schoenfeld Individual Test p: 0.961
Schoenfeld Individual Test p: 0.423
Schoenfeld Individual Test p: 0.0384
Beta(t) for frailty
Beta(t) for h1rural
Beta(t) for r1smoke3
Beta(t) for r1iadl
Beta(t) for r1agey
Beta(t) for r1socwk
Beta(t) for r1drink3
Beta(t) for r1adlab_c
Beta(t) for Sex1
Beta(t) for r1livingalone
Beta(t) for r1hibpe
Beta(t) for edu
Beta(t) for r1work
Beta(t) for r1diabe
Beta(t) for married
Beta(t) for shlta
Beta(t) for Sleeptime
Time

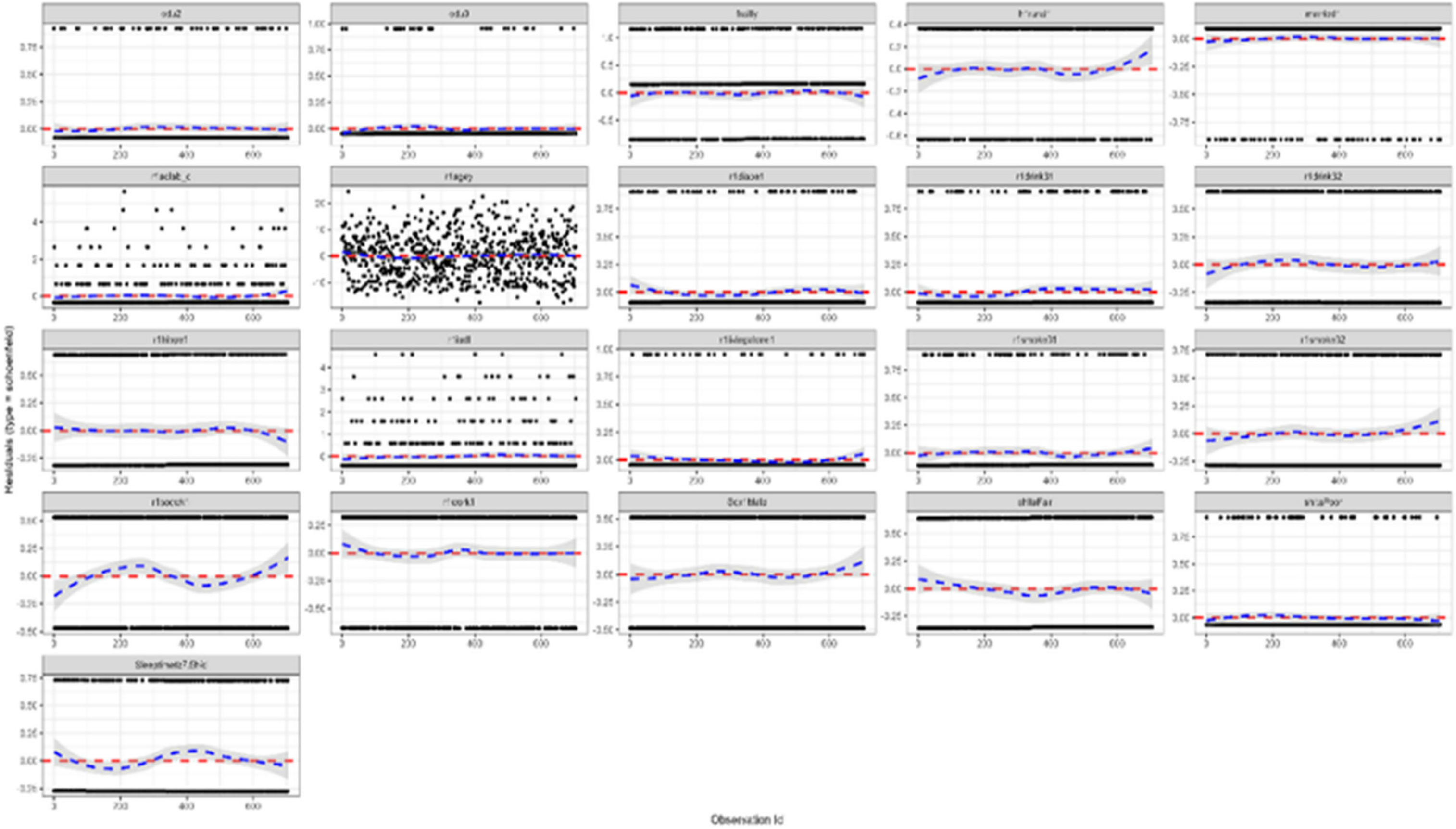
r1agey
r1drink31
r1drink32
r1smoke31
r1smoke32
Residuals (type = schoenfeld)
Observation Id

Supplementary Figure S9. The proportional hazards (PHs) assumption using Schoenfeld residuals test for each covariate for Cox model for the relationship between frailty index and gastrointestinal disease incidence (CHARLS).

Proportional Hazards Assumption Tests

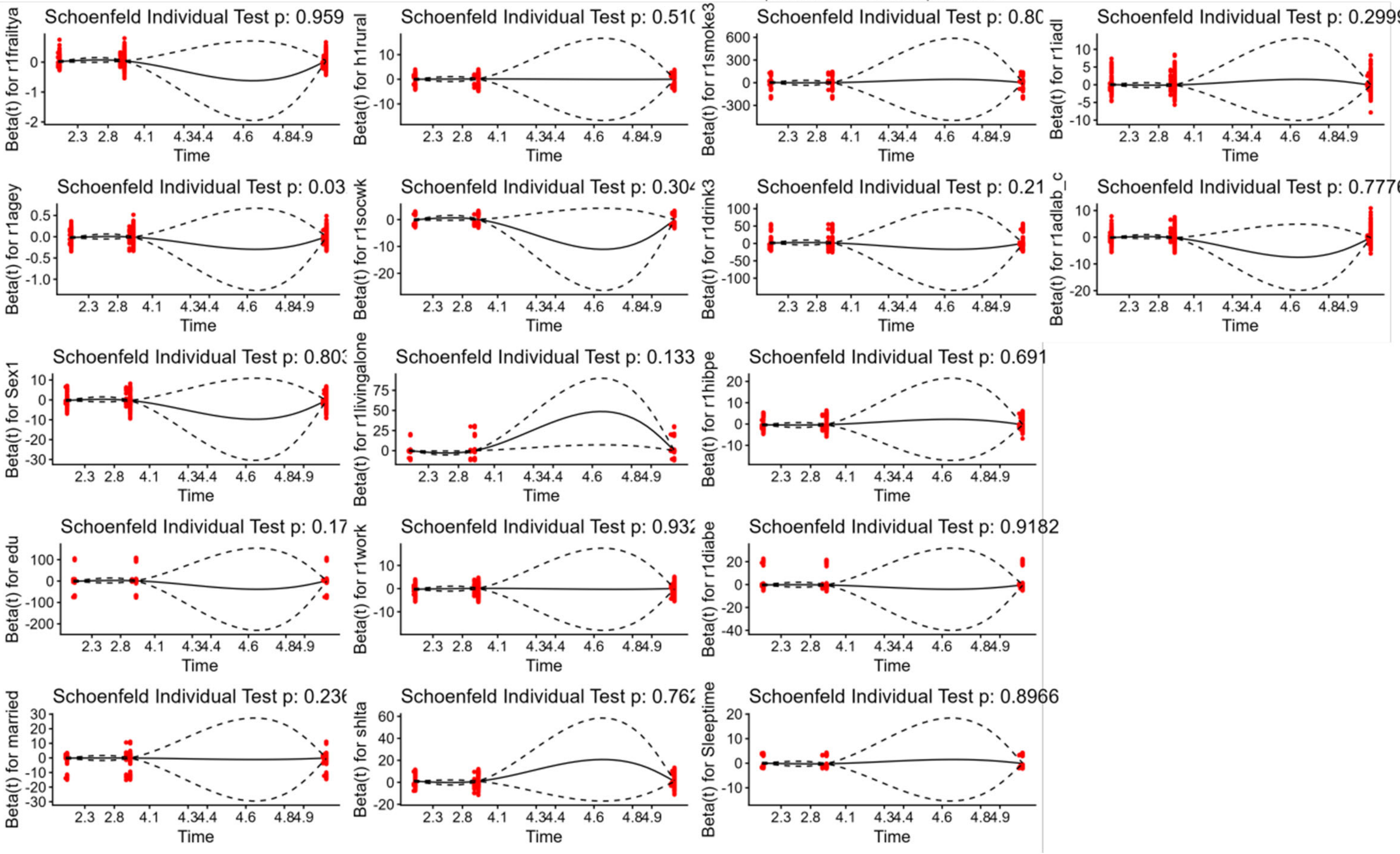

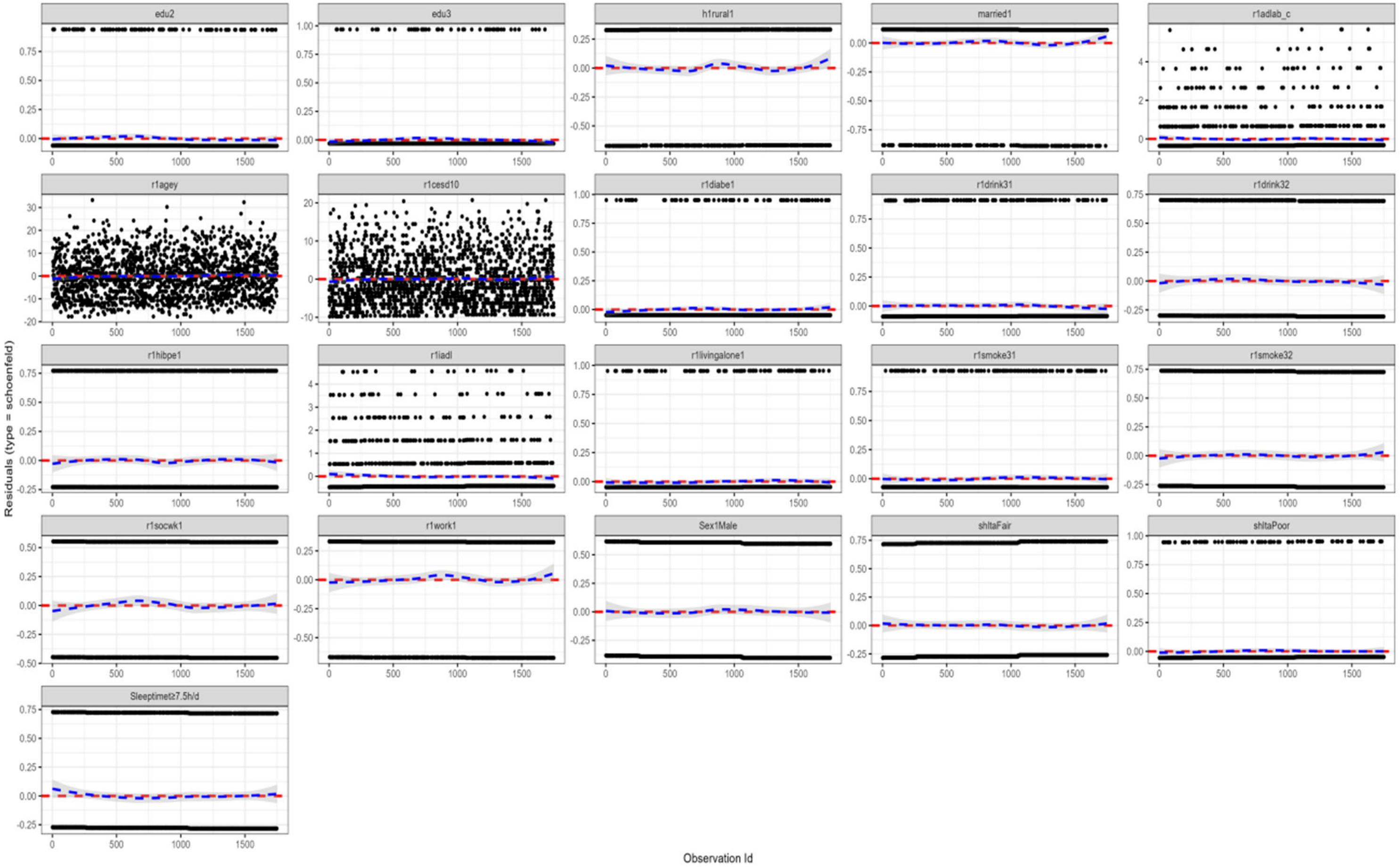

edu2
edu3
h1rural1
married1
r1adlab_c
r1agey
r1cesd10
r1diabe1
r1drink31
r1drink32
r1hibpe1
r1iadl
r1livingalone1
r1smoke31
r1smoke32
r1socwk1
r1work1
Sex1Male
shltaFair
shltaPoor
Sleeptime≥7.5h/d
Residuals (type = schoenfeld)
Observation Id

Supplementary Figure S10. The proportional hazards (PHs) assumption using Schoenfeld residuals test for each covariate for Cox model for the relationship between frailty index and liver disease incidence (CHARLS).

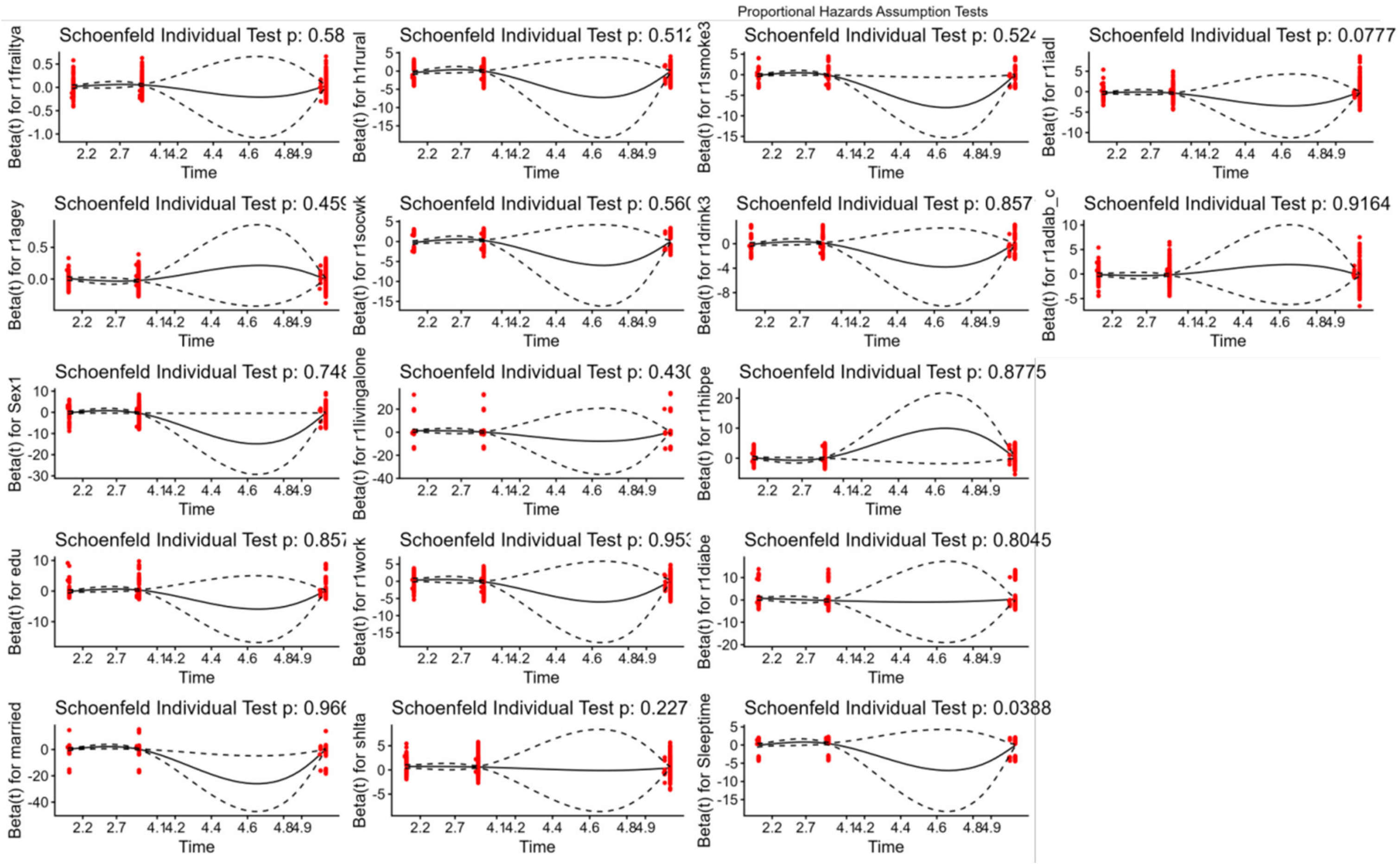
Proportional Hazards Assumption Tests
Schoenfeld Individual Test p: 0.58
Schoenfeld Individual Test p: 0.512
Schoenfeld Individual Test p: 0.524
Schoenfeld Individual Test p: 0.0777
Schoenfeld Individual Test p: 0.459
Schoenfeld Individual Test p: 0.560
Schoenfeld Individual Test p: 0.857
Schoenfeld Individual Test p: 0.9164
Schoenfeld Individual Test p: 0.748
Schoenfeld Individual Test p: 0.430
Schoenfeld Individual Test p: 0.8775
Schoenfeld Individual Test p: 0.857
Schoenfeld Individual Test p: 0.953
Schoenfeld Individual Test p: 0.8045
Schoenfeld Individual Test p: 0.966
Schoenfeld Individual Test p: 0.227
Schoenfeld Individual Test p: 0.0388
Beta(t) for r1frailtya
Beta(t) for h1rural
Beta(t) for r1smoke3
Beta(t) for r1iadl
Beta(t) for r1agey
Beta(t) for r1socwk
Beta(t) for r1drink3
Beta(t) for r1adlab_c
Beta(t) for Sex1
Beta(t) for r1livingalone
Beta(t) for r1hibpe
Beta(t) for edu
Beta(t) for r1work
Beta(t) for r1diabe
Beta(t) for married
Beta(t) for shlta
Beta(t) for Sleeptime
Time

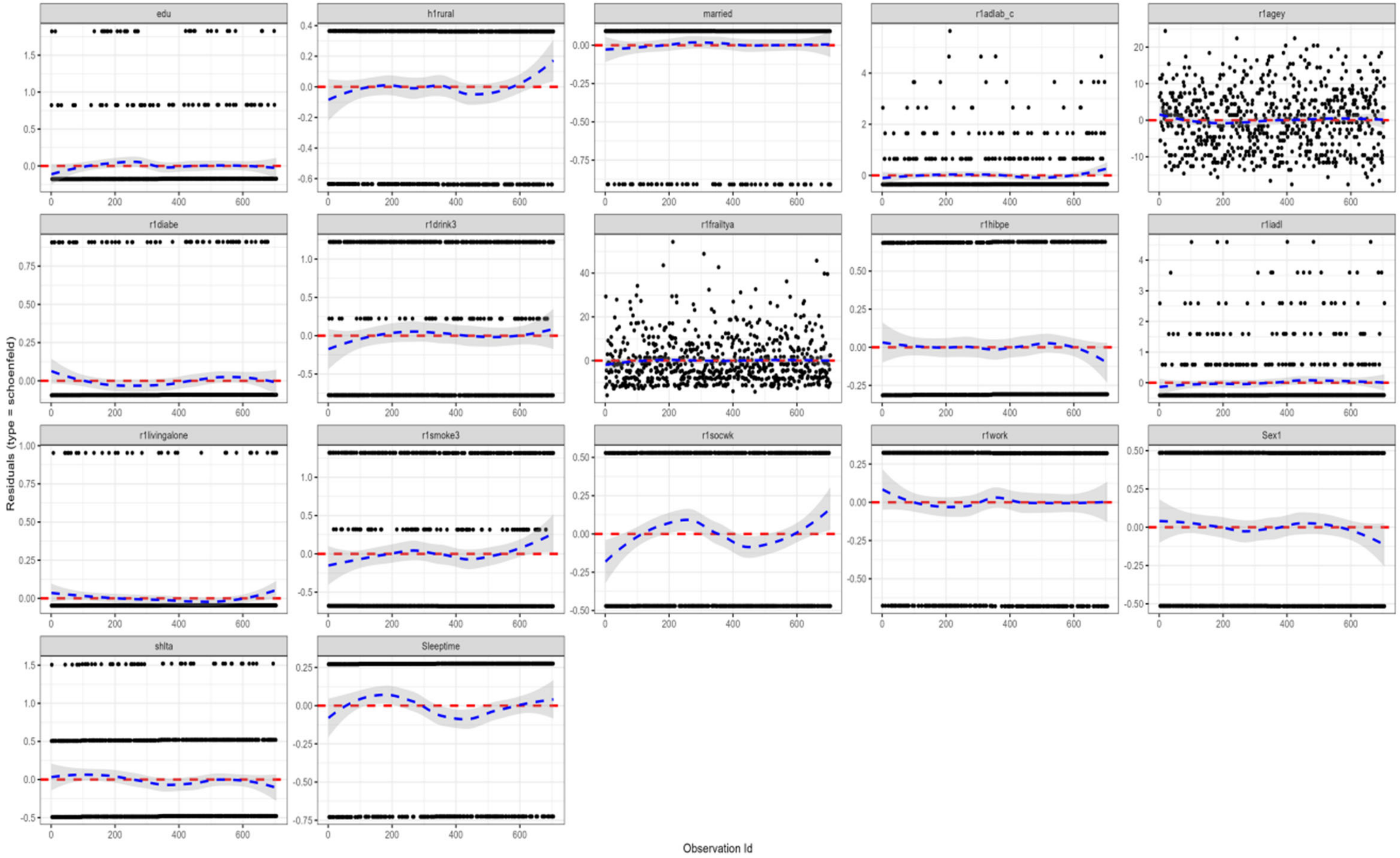
edu
h1rural
married
r1adlab_c
r1agey
r1diabe
r1drink3
r1frailtya
r1hibpe
r1iadl
r1livingalone
r1smoke3
r1socwk
r1work
Sex1
shlta
Sleeptime
Residuals (type = schoenfeld)
Observation Id

Supplementary Figure S11. The proportional hazards (PHs) assumption using Schoenfeld residuals test for each covariate for Cox model for the relationship between depression and gastrointestinal disease incidence (CHARLS).

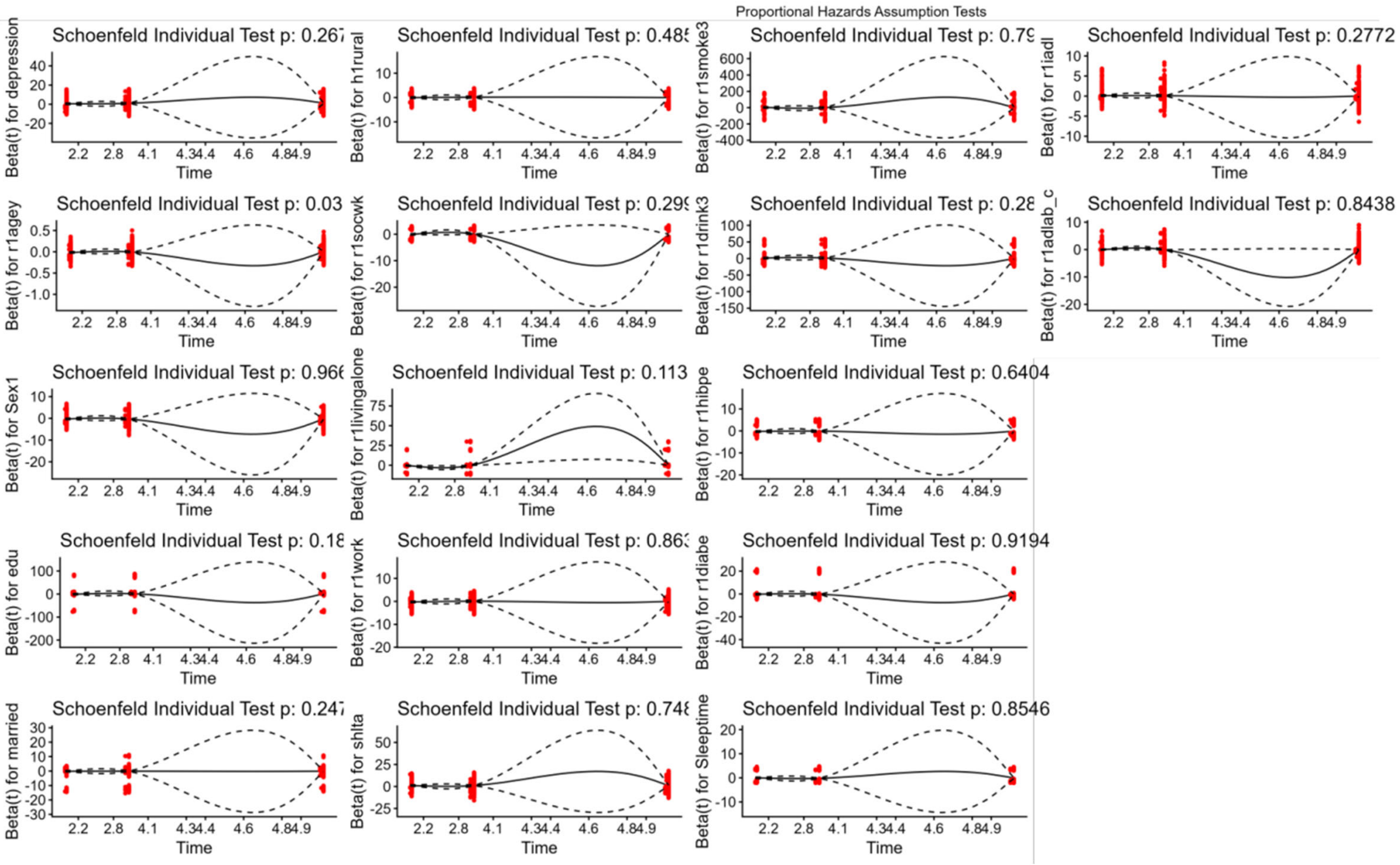
Proportional Hazards Assumption Tests
Schoenfeld Individual Test p: 0.267
Beta(t) for depression
Schoenfeld Individual Test p: 0.485
Beta(t) for h1rural
Schoenfeld Individual Test p: 0.79
Beta(t) for r1smoke3
Schoenfeld Individual Test p: 0.2772
Beta(t) for r1iadl
Schoenfeld Individual Test p: 0.03
Beta(t) for r1agey
Schoenfeld Individual Test p: 0.299
Beta(t) for r1socwk
Schoenfeld Individual Test p: 0.28
Beta(t) for r1drink3
Schoenfeld Individual Test p: 0.8438
Beta(t) for r1adlab_c
Schoenfeld Individual Test p: 0.966
Beta(t) for Sex1
Schoenfeld Individual Test p: 0.113
Beta(t) for r1livingalone
Schoenfeld Individual Test p: 0.6404
Beta(t) for r1hibpe
Schoenfeld Individual Test p: 0.18
Beta(t) for edu
Schoenfeld Individual Test p: 0.863
Beta(t) for r1work
Schoenfeld Individual Test p: 0.9194
Beta(t) for r1diabe
Schoenfeld Individual Test p: 0.247
Beta(t) for married
Schoenfeld Individual Test p: 0.748
Beta(t) for shlta
Schoenfeld Individual Test p: 0.8546
Beta(t) for Sleeptime
Time

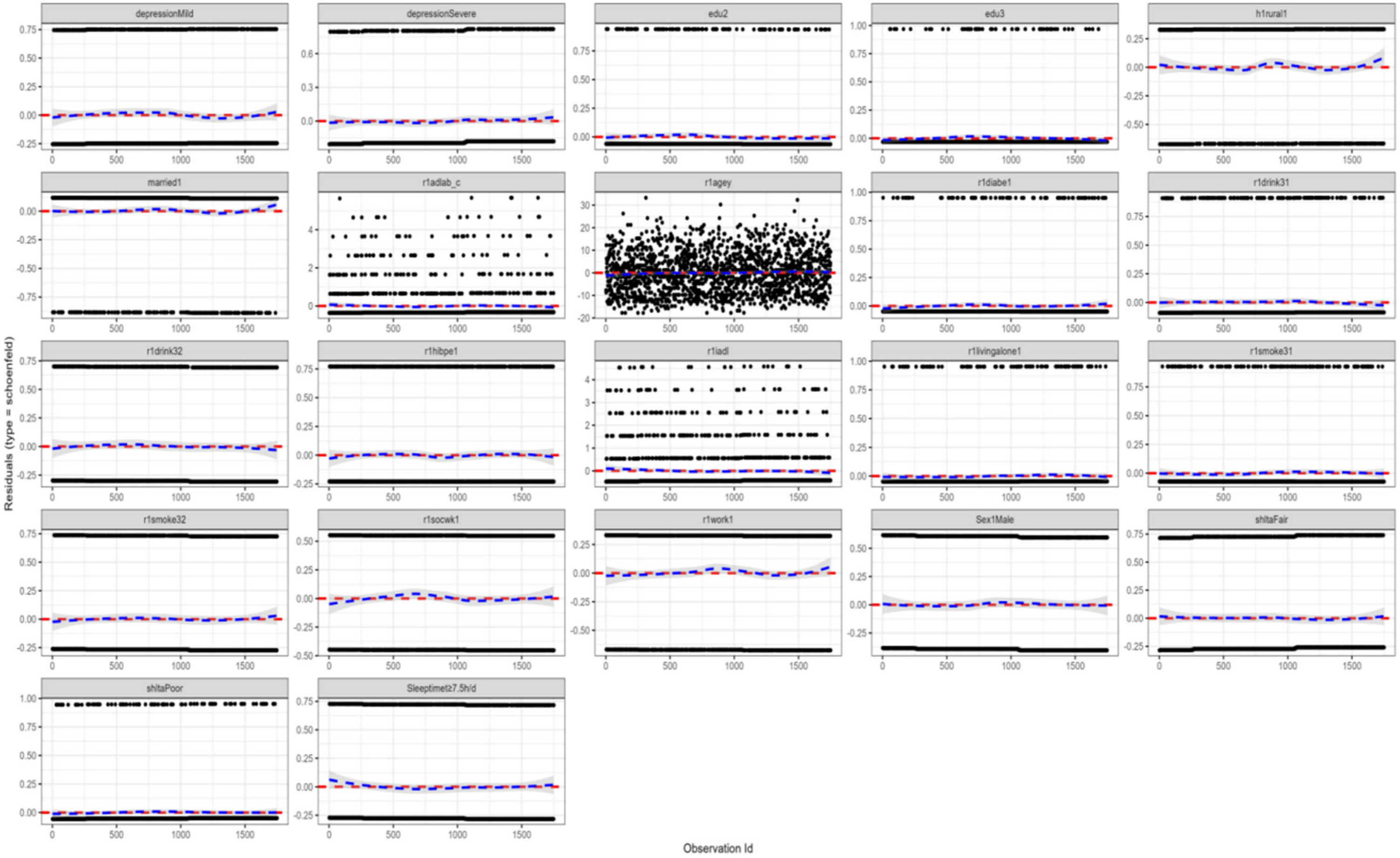
depressionMild
depressionSevere
edu2
edu3
h1rural1
married1
r1adlab_c
r1agey
r1diabe1
r1drink31
r1drink32
r1hibpe1
r1iadl
r1livingalone1
r1smoke31
r1smoke32
r1socwk1
r1work1
Sex1Male
shltaFair
shltaPoor
Sleeptime≥7.5h/d
Residuals (type = schoenfeld)
Observation Id

Supplementary Figure S12. The proportional hazards (PHs) assumption using Schoenfeld residuals test for each covariate for Cox model for the relationship between depression and liver disease incidence (CHARLS).

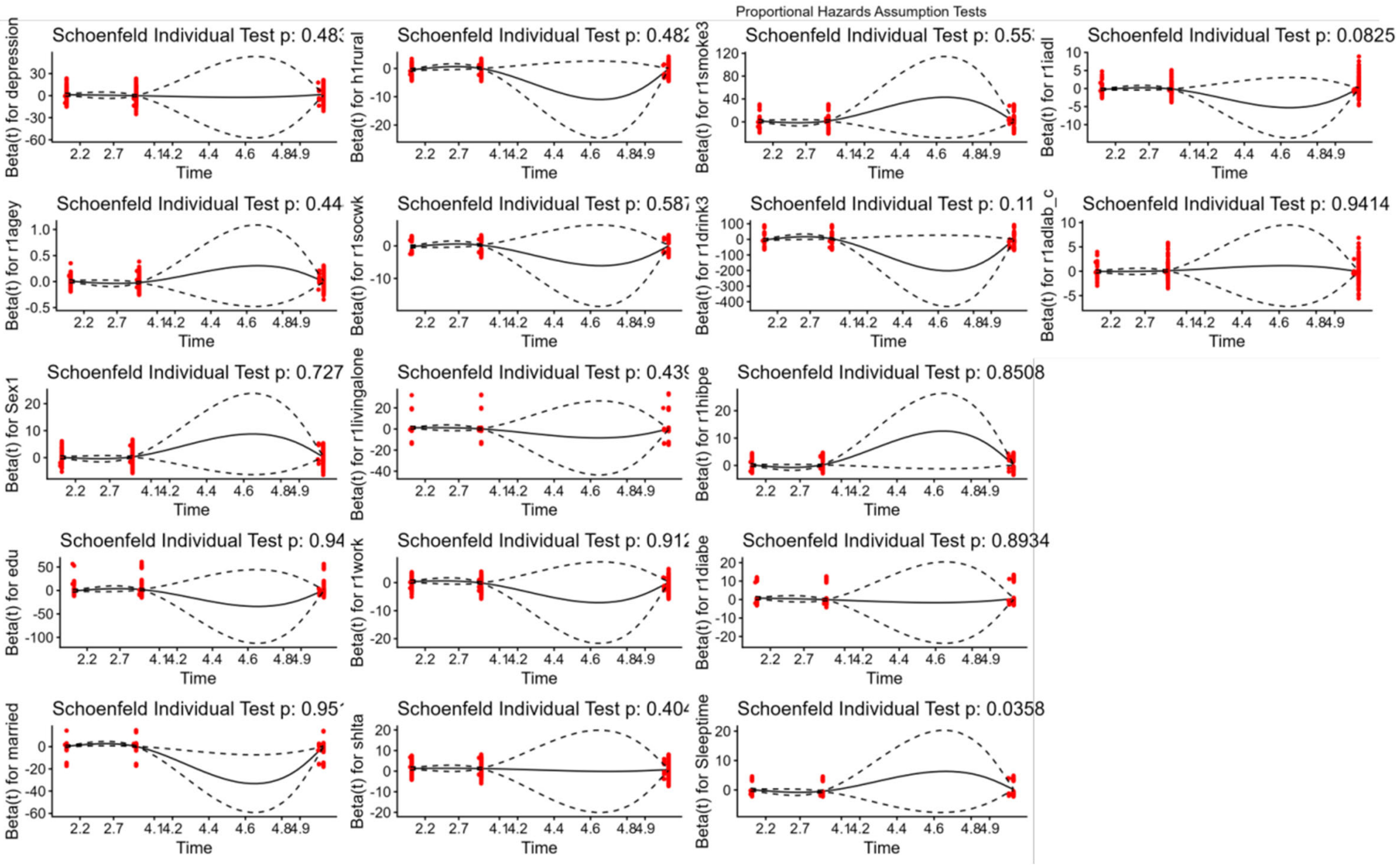
Proportional Hazards Assumption Tests
Schoenfeld Individual Test p: 0.48
Beta(t) for depression
Schoenfeld Individual Test p: 0.48
Beta(t) for h1rural
Schoenfeld Individual Test p: 0.55
Beta(t) for r1smoke3
Schoenfeld Individual Test p: 0.0825
Beta(t) for r1iadl
Schoenfeld Individual Test p: 0.44
Beta(t) for r1agey
Schoenfeld Individual Test p: 0.58
Beta(t) for r1socwk
Schoenfeld Individual Test p: 0.11
Beta(t) for r1drink3
Schoenfeld Individual Test p: 0.9414
Beta(t) for r1adlab_c
Schoenfeld Individual Test p: 0.727
Beta(t) for Sex1
Schoenfeld Individual Test p: 0.43
Beta(t) for r1livingalone
Schoenfeld Individual Test p: 0.8508
Beta(t) for r1hibpe
Schoenfeld Individual Test p: 0.94
Beta(t) for edu
Schoenfeld Individual Test p: 0.91
Beta(t) for r1work
Schoenfeld Individual Test p: 0.8934
Beta(t) for r1diabe
Schoenfeld Individual Test p: 0.95
Beta(t) for married
Schoenfeld Individual Test p: 0.40
Beta(t) for shlta
Schoenfeld Individual Test p: 0.0358
Beta(t) for Sleeptime
Time

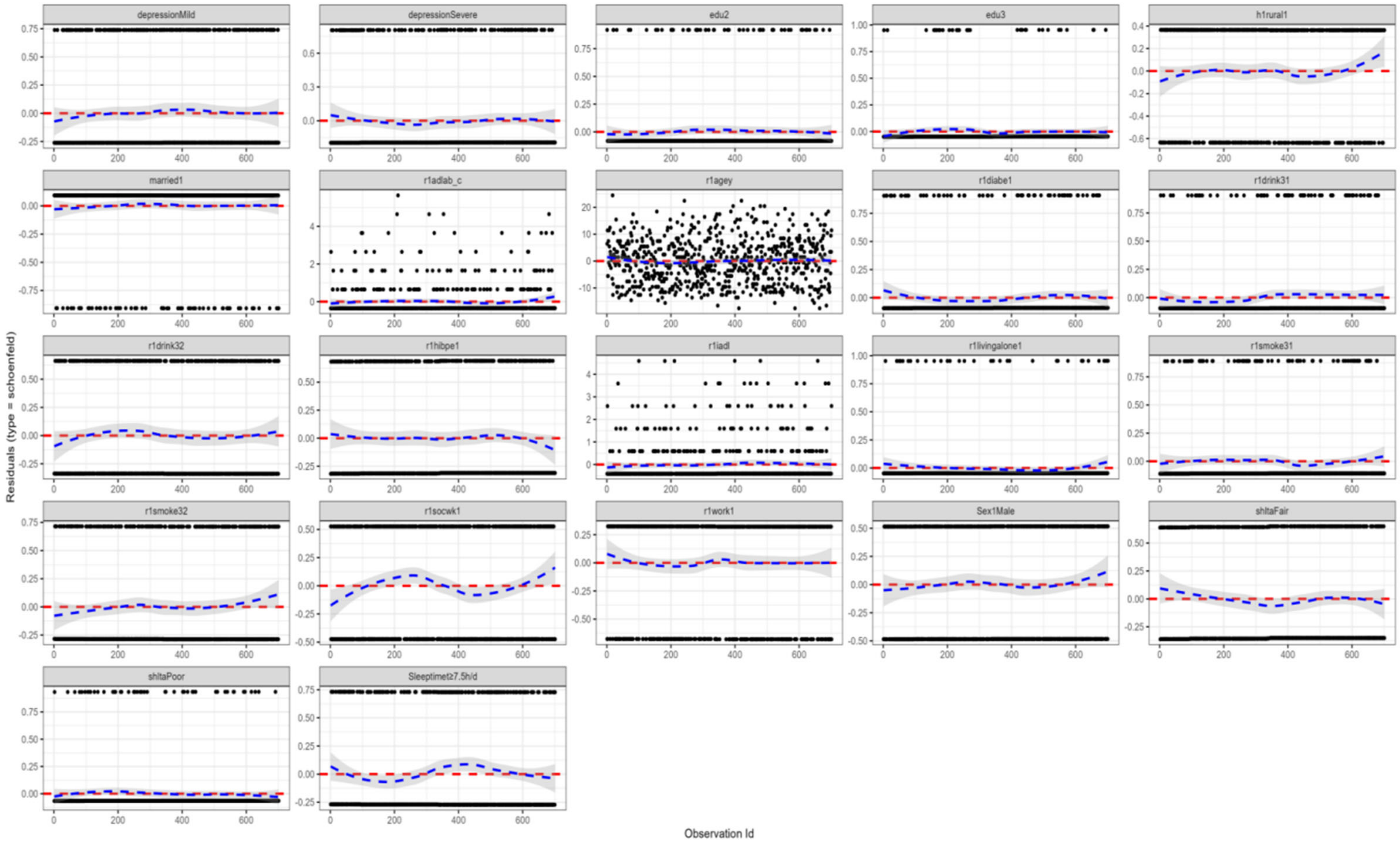
depressionMild
depressionSevere
edu2
edu3
h1rural1
married1
r1adlab_c
r1agey
r1diabe1
r1drink31
r1drink32
r1hibpe1
r1iadl
r1livingalone1
r1smoke31
r1smoke32
r1socwk1
r1work1
Sex1Male
shltaFair
shltaPoor
Sleeptime≥7.5h/d
Residuals (type = schoenfeld)
Observation Id

Supplementary Figure S13. The proportional hazards (PHs) assumption using Schoenfeld residuals test for each covariate for Cox model for the relationship between CES-D10 score and gastrointestinal disease incidence (CHARLS).

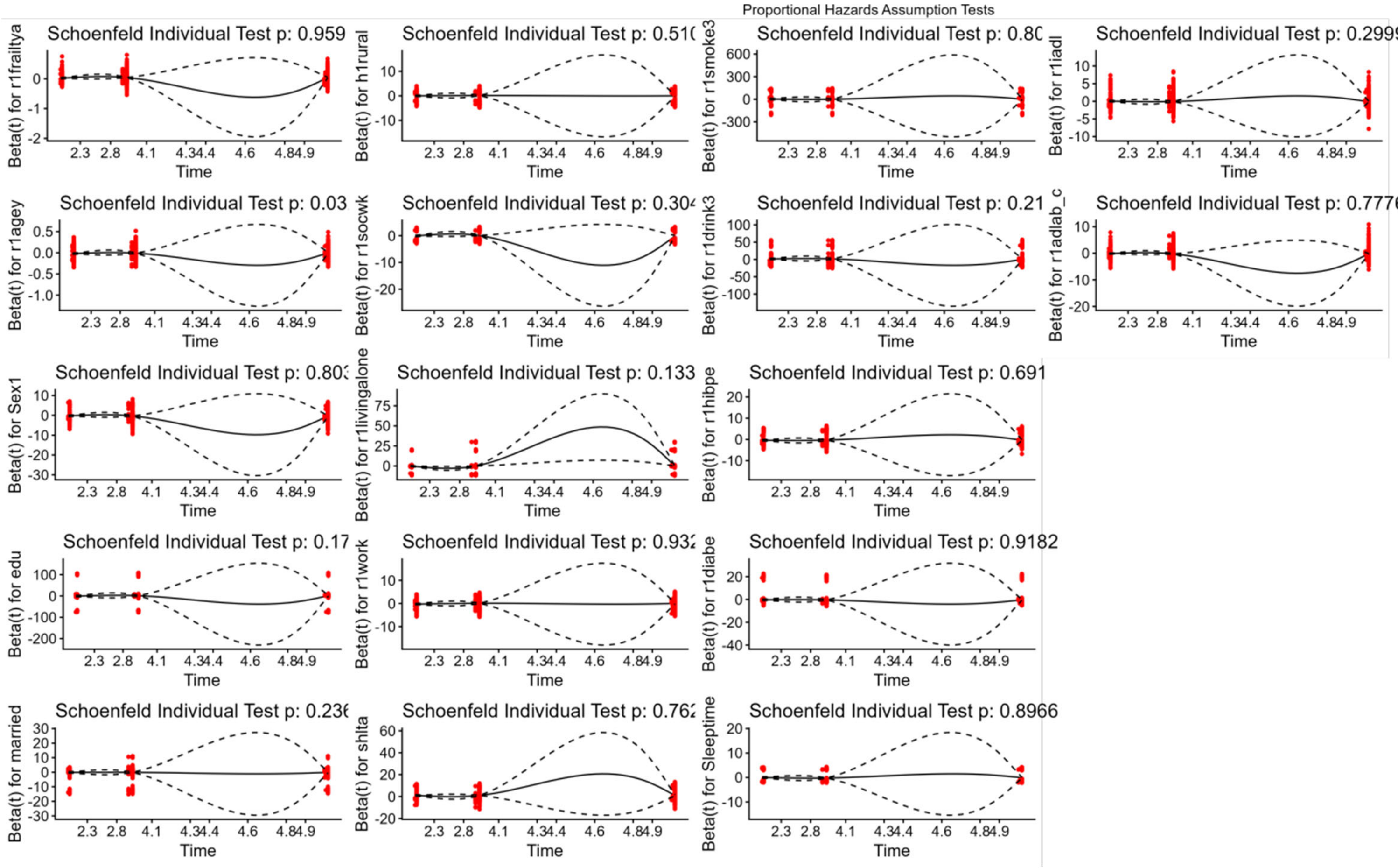

Proportional Hazards Assumption Tests
Schoenfeld Individual Test p: 0.959
Schoenfeld Individual Test p: 0.51
Schoenfeld Individual Test p: 0.80
Schoenfeld Individual Test p: 0.2999
Schoenfeld Individual Test p: 0.03
Schoenfeld Individual Test p: 0.304
Schoenfeld Individual Test p: 0.21
Schoenfeld Individual Test p: 0.7776
Schoenfeld Individual Test p: 0.803
Schoenfeld Individual Test p: 0.133
Schoenfeld Individual Test p: 0.691
Schoenfeld Individual Test p: 0.17
Schoenfeld Individual Test p: 0.932
Schoenfeld Individual Test p: 0.9182
Schoenfeld Individual Test p: 0.236
Schoenfeld Individual Test p: 0.762
Schoenfeld Individual Test p: 0.8966
Beta(t) for r1frailtya
Beta(t) for h1rural
Beta(t) for r1smoke3
Beta(t) for r1iadl
Beta(t) for r1agey
Beta(t) for r1socwk
Beta(t) for r1drink3
Beta(t) for r1adlab_c
Beta(t) for Sex1
Beta(t) for r1livingalone
Beta(t) for r1hibpe
Beta(t) for edu
Beta(t) for r1work
Beta(t) for r1diabe
Beta(t) for married
Beta(t) for shlta
Beta(t) for Sleeptime
Time

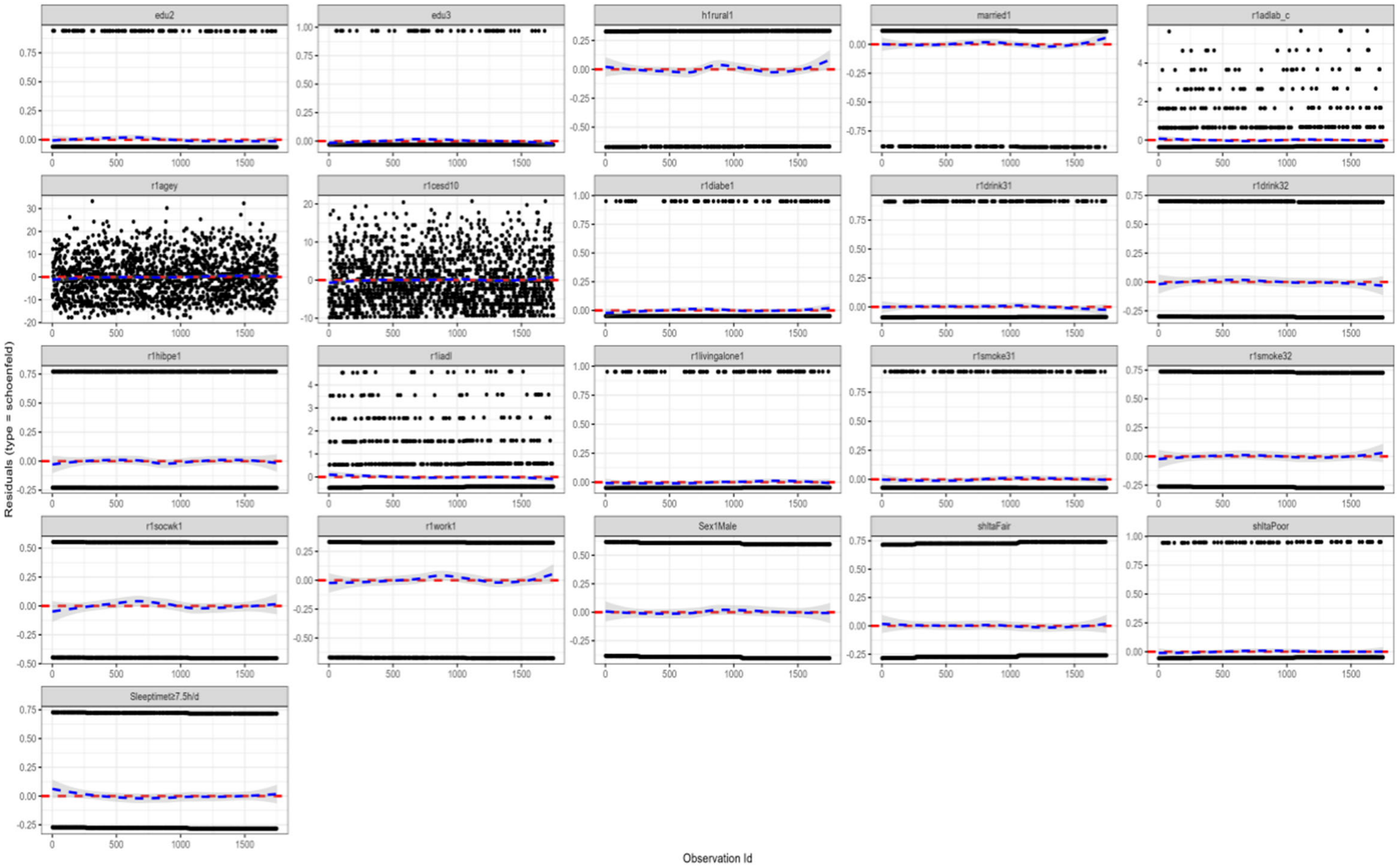
edu2
edu3
h1rural1
married1
r1adlab_c
r1agey
r1cesd10
r1diabe1
r1drink31
r1drink32
r1hibpe1
r1iadl
r1livingalone1
r1smoke31
r1smoke32
r1socwk1
r1work1
Sex1Male
shltaFair
shltaPoor
Sleeptime≥7.5h/d
Residuals (type = schoenfeld)
Observation Id

Supplementary Figure S14. The proportional hazards (PHs) assumption using Schoenfeld residuals test for each covariate for Cox model for the relationship between CES-D10 score and liver disease incidence (CHARLS).

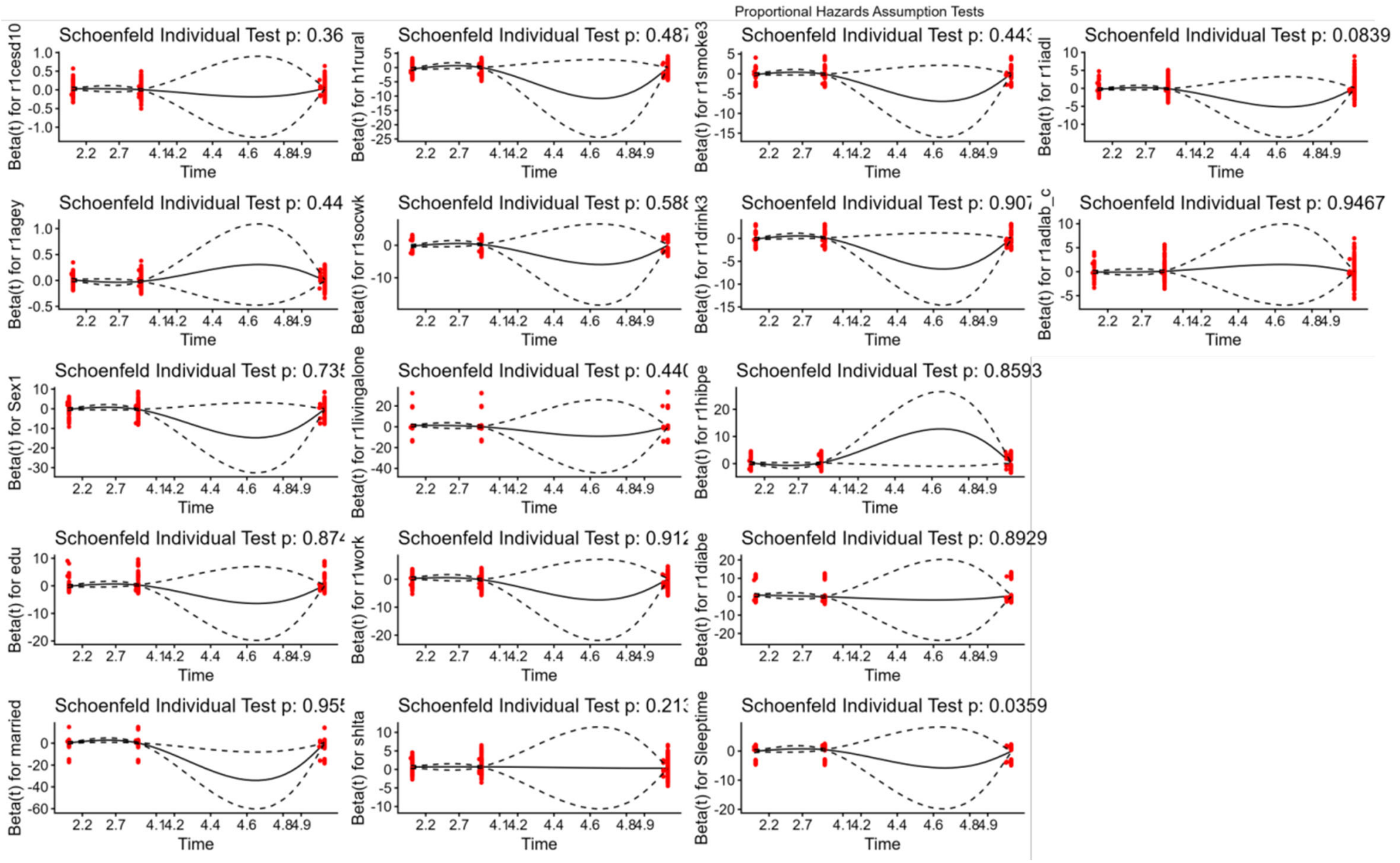
Proportional Hazards Assumption Tests
Schoenfeld Individual Test p: 0.36
Beta(t) for r1cesd10
Schoenfeld Individual Test p: 0.487
Beta(t) for h1rural
Schoenfeld Individual Test p: 0.443
Beta(t) for r1smoke3
Schoenfeld Individual Test p: 0.0839
Beta(t) for r1iadl
Schoenfeld Individual Test p: 0.44
Beta(t) for r1agey
Schoenfeld Individual Test p: 0.588
Beta(t) for r1socwk
Schoenfeld Individual Test p: 0.907
Beta(t) for r1drink3
Schoenfeld Individual Test p: 0.9467
Beta(t) for r1adlab_c
Schoenfeld Individual Test p: 0.735
Beta(t) for Sex1
Schoenfeld Individual Test p: 0.440
Beta(t) for r1livingalone
Schoenfeld Individual Test p: 0.8593
Beta(t) for r1hibpe
Schoenfeld Individual Test p: 0.874
Beta(t) for edu
Schoenfeld Individual Test p: 0.912
Beta(t) for r1work
Schoenfeld Individual Test p: 0.8929
Beta(t) for r1diabe
Schoenfeld Individual Test p: 0.955
Beta(t) for married
Schoenfeld Individual Test p: 0.213
Beta(t) for shlta
Schoenfeld Individual Test p: 0.0359
Beta(t) for Sleeptime
Time

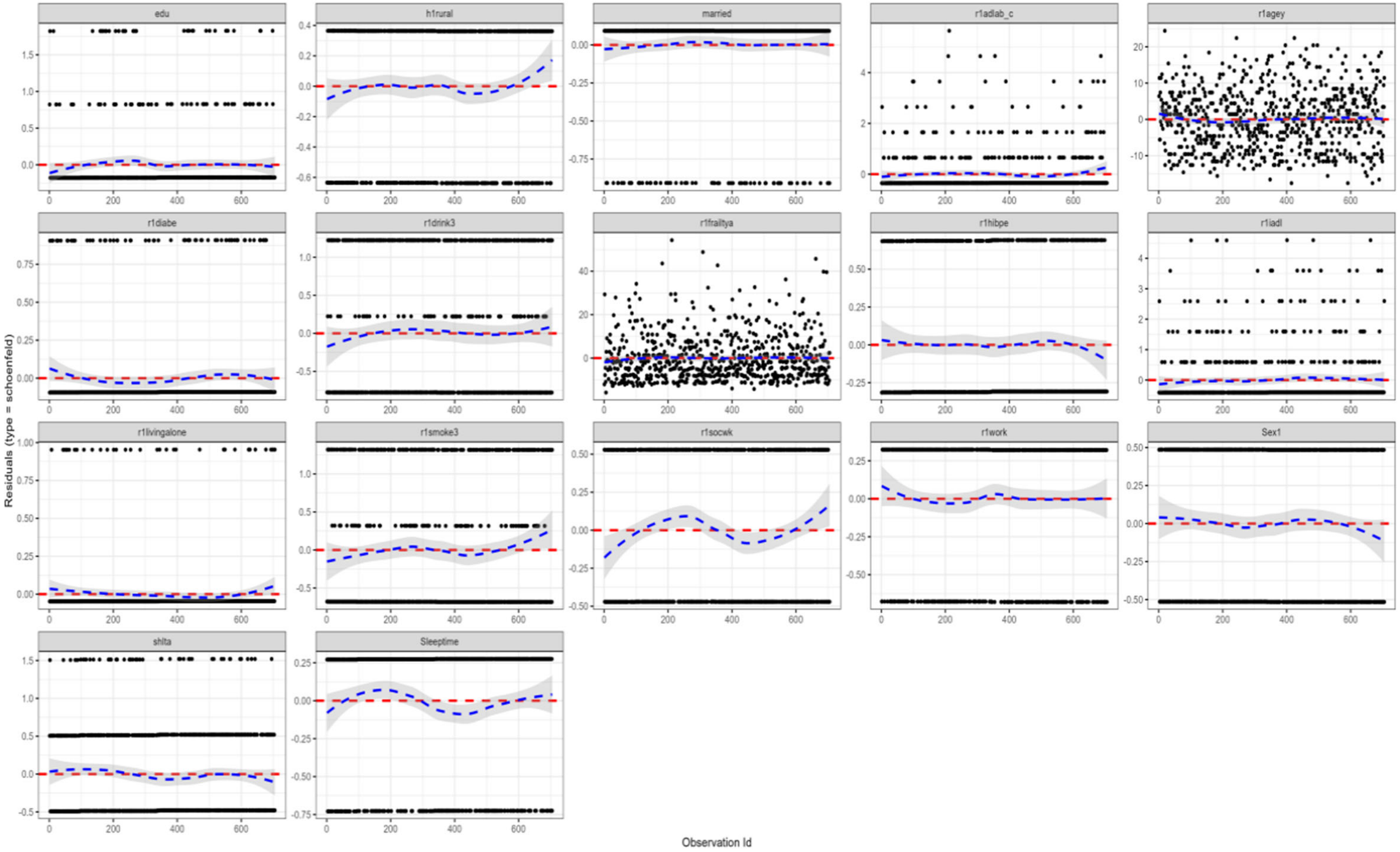
edu
h1rural
married
r1adlab_c
r1agey
r1diabe
r1drink3
r1frailtya
r1hibpe
r1iadl
r1livingalone
r1smoke3
r1socwk
r1work
Sex1
shlta
Sleeptime
Residuals (type = schoenfeld)
Observation Id

Supplementary Figure S15. Subgroup analysis of the associations between frail symptoms and gastrointestinal or liver disease.

Variable	Count	Percent	Levels	P value	P for interaction	HR_CI
Overall	9939	100	None			
			Pre-frailty	<0.001		1.51 (1.26-1.81)
			Frailty	<0.001		2.42 (1.75-3.34)
age_group					0.576	
>65	1876	18.9	None			
			Pre-frailty	0.514		1.18 (0.72-1.92)
			Frailty	0.056		1.97 (0.98-3.96)
<=65	8063	81.1	None			
			Pre-frailty	<0.001		1.58 (1.30-1.91)
			Frailty	<0.001		2.49 (1.71-3.63)
sex					0.362	
Female	5396	54.3	None			
			Pre-frailty	0.001		1.59 (1.22-2.07)
			Frailty	<0.001		2.26 (1.46-3.51)
Male	4543	45.7	None			
			Pre-frailty	0.001		1.50 (1.17-1.92)
			Frailty	<0.001		2.88 (1.75-4.75)
education					0.383	
Below high school	8843	89	None			
			Pre-frailty	<0.001		1.49 (1.23-1.81)
			Frailty	<0.001		2.52 (1.80-3.54)
High school	760	7.6	None			
			Pre-frailty	0.017		2.07 (1.14-3.77)
			Frailty	0.186		2.60 (0.63-10.75)
Above high school	336	3.4	None			
			Pre-frailty	0.354		1.51 (0.63-3.64)
			Frailty	0.998		0.00 (0.00-Inf)
marriage					0.59	
Other	980	9.9	None			
			Pre-frailty	0.102		1.78 (0.89-3.55)
			Frailty	0.03		3.04 (1.12-8.29)
Married	8959	90.1	None			
			Pre-frailty	<0.001		1.50 (1.25-1.81)
			Frailty	<0.001		2.35 (1.66-3.33)
residence					0.362	
Rural	6482	65.2	None			
			Pre-frailty	<0.001		1.65 (1.31-2.07)
			Frailty	<0.001		2.48 (1.66-3.69)
Urban	3457	34.8	None			
			Pre-frailty	0.063		1.32 (0.98-1.76)
			Frailty	0.002		2.41 (1.38-4.22)

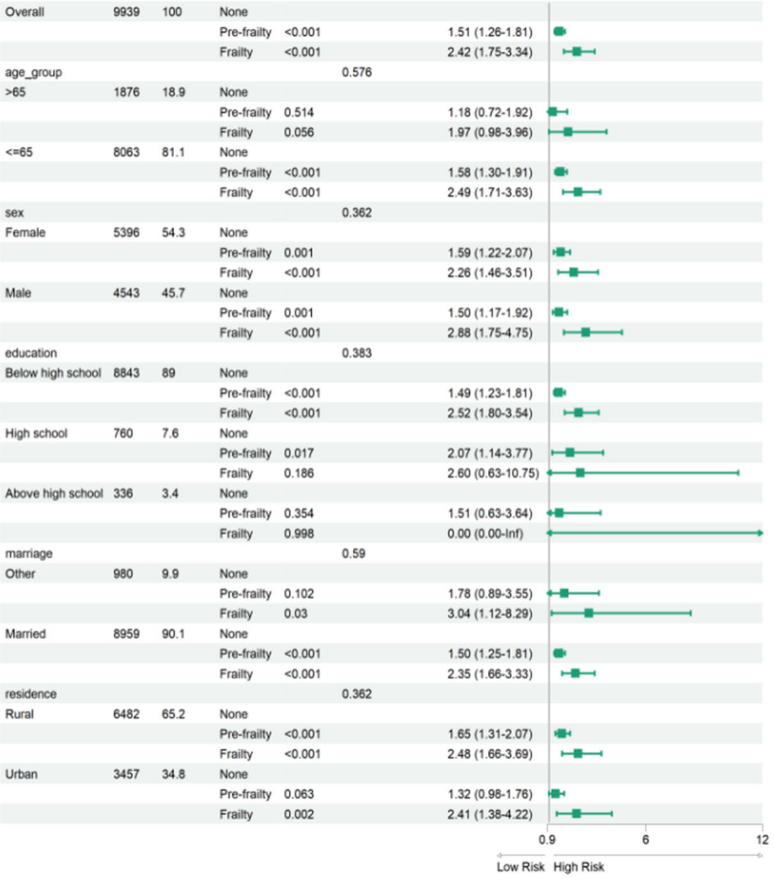

(A)

Variable	Count	Percent	Levels	P value	P for interaction	HR_CI
Overall	9939	100	None			
			Pre-frailty	<0.001		1.51 (1.26-1.81)
			Frailty	<0.001		2.42 (1.75-3.34)
age_group					0.576	
>65	1876	18.9	None			
			Pre-frailty	0.514		1.18 (0.72-1.92)
			Frailty	0.056		1.97 (0.98-3.96)
<=65	8063	81.1	None			
			Pre-frailty	<0.001		1.58 (1.30-1.91)
			Frailty	<0.001		2.49 (1.71-3.63)
sex					0.362	
Female	5396	54.3	None			
			Pre-frailty	0.001		1.59 (1.22-2.07)
			Frailty	<0.001		2.26 (1.46-3.51)
Male	4543	45.7	None			
			Pre-frailty	0.001		1.50 (1.17-1.92)
			Frailty	<0.001		2.88 (1.75-4.75)
education					0.383	
Below high school	8843	89	None			
			Pre-frailty	<0.001		1.49 (1.23-1.81)
			Frailty	<0.001		2.52 (1.80-3.54)
High school	760	7.6	None			
			Pre-frailty	0.017		2.07 (1.14-3.77)
			Frailty	0.186		2.60 (0.63-10.75)
Above high school	336	3.4	None			
			Pre-frailty	0.354		1.51 (0.63-3.64)
			Frailty	0.998		0.00 (0.00-Inf)
marriage					0.59	
Other	980	9.9	None			
			Pre-frailty	0.102		1.78 (0.89-3.55)
			Frailty	0.03		3.04 (1.12-8.29)
Married	8959	90.1	None			
			Pre-frailty	<0.001		1.50 (1.25-1.81)
			Frailty	<0.001		2.35 (1.66-3.33)
residence					0.362	
Rural	6482	65.2	None			
			Pre-frailty	<0.001		1.65 (1.31-2.07)
			Frailty	<0.001		2.48 (1.66-3.69)
Urban	3457	34.8	None			
			Pre-frailty	0.063		1.32 (0.98-1.76)
			Frailty	0.002		2.41 (1.38-4.22)

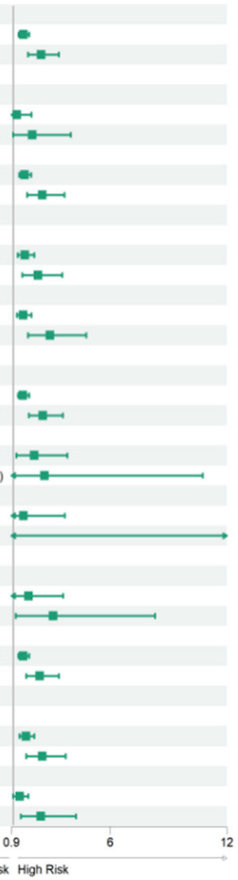

(B)

Note. Subgroup analysis of the associations between frail symptoms and gastrointestinal (A) or liver disease (B).

Supplementary Figure S16. Subgroup analysis of the associations between depressive symptoms and gastrointestinal (A) or liver disease (B).

Variable	Count	Percent	Levels	P value	P for interaction	HR_CI
Overall	7934	100	None			
			Mild	<0.001		1.31 (1.17-1.48)
			Severe	<0.001		1.66 (1.44-1.91)
age_group					0.943	
<=65	6411	80.8	None			
			Mild	<0.001		1.32 (1.15-1.50)
			Severe	<0.001		1.66 (1.42-1.95)
>65	1523	19.2	None			
			Mild	0.017		1.38 (1.06-1.79)
			Severe	0.001		1.66 (1.23-2.25)
sex					0.803	
Female	4190	52.8	None			
			Mild	<0.001		1.33 (1.14-1.54)
			Severe	<0.001		1.62 (1.36-1.93)
Male	3744	47.2	None			
			Mild	0.007		1.31 (1.08-1.59)
			Severe	<0.001		1.78 (1.40-2.27)
education					0.621	
Below high school	7010	88.4	None			
			Mild	<0.001		1.32 (1.16-1.49)
			Severe	<0.001		1.64 (1.42-1.89)
High school	640	8.1	None			
			Mild	0.443		1.23 (0.73-2.06)
			Severe	0.065		2.07 (0.96-4.49)
Above high school	284	3.6	None			
			Mild	0.363		1.43 (0.66-3.08)
			Severe	0.022		3.80 (1.22-11.88)
marriage					0.909	
Other	780	9.8	None			
			Mild	0.499		1.13 (0.79-1.62)
			Severe	0.078		1.40 (0.96-2.03)
Married	7154	90.2	None			
			Mild	<0.001		1.33 (1.18-1.51)
			Severe	<0.001		1.69 (1.45-1.96)
residence					0.564	
Rural	5094	64.2	None			
			Mild	<0.001		1.31 (1.13-1.51)
			Severe	<0.001		1.63 (1.38-1.92)
Urban	2840	35.8	None			
			Mild	0.008		1.33 (1.08-1.63)
			Severe	<0.001		1.77 (1.35-2.31)

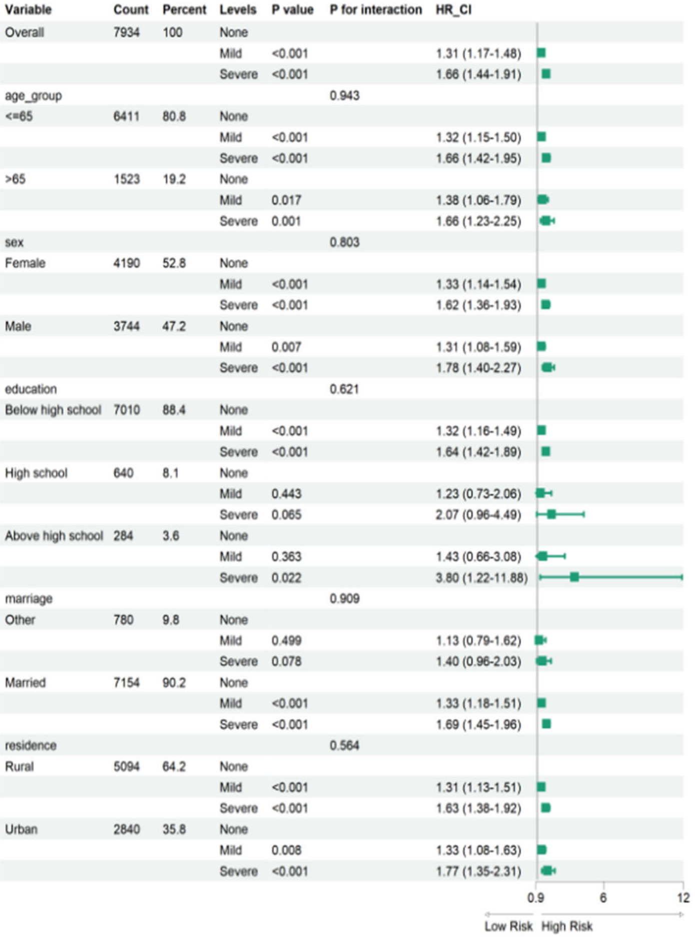

(A)

Variable	Count	Percent	Levels	P value	P for interaction	HR_CI
Overall	9891	100	None			
			Mild	0.036		1.22 (1.01-1.47)
			Severe	0.007		1.36 (1.09-1.70)
age_group					0.954	
>65	1875	19	None			
			Mild	0.818		1.06 (0.67-1.67)
			Severe	0.817		1.07 (0.62-1.82)
<=65	8016	81	None			
			Mild	0.026		1.26 (1.03-1.54)
			Severe	0.006		1.42 (1.11-1.81)
sex					0.952	
Female	5380	54.4	None			
			Mild	0.151		1.21 (0.93-1.56)
			Severe	0.118		1.27 (0.94-1.70)
Male	4511	45.6	None			
			Mild	0.093		1.26 (0.96-1.66)
			Severe	0.01		1.59 (1.12-2.25)
education					0.333	
Below high school	8807	89	None			
			Mild	0.08		1.19 (0.98-1.45)
			Severe	0.031		1.29 (1.02-1.63)
High school	753	7.6	None			
			Mild	0.283		1.43 (0.74-2.77)
			Severe	0.147		1.92 (0.79-4.66)
Above high school	331	3.3	None			
			Mild	0.71		1.22 (0.42-3.53)
			Severe	0.2		2.57 (0.61-10.89)
marriage					0.896	
Other	980	9.9	None			
			Mild	0.994		1.00 (0.53-1.89)
			Severe	0.404		1.32 (0.69-2.53)
Married	8911	90.1	None			
			Mild	0.031		1.24 (1.02-1.50)
			Severe	0.012		1.36 (1.07-1.72)
residence					0.697	
Rural	6456	65.3	None			
			Mild	0.112		1.21 (0.96-1.53)
			Severe	0.006		1.44 (1.11-1.88)
Urban	3435	34.7	None			
			Mild	0.151		1.25 (0.92-1.70)
			Severe	0.501		1.16 (0.75-1.78)

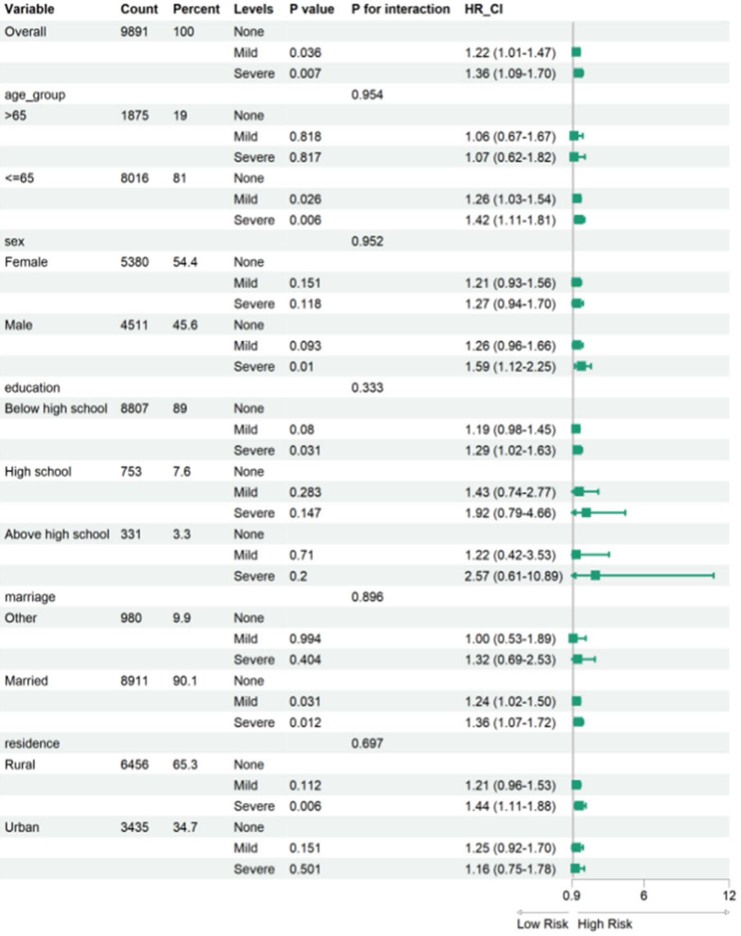

(B)

Note. Subgroup analysis of the associations between depressive symptoms and gastrointestinal (A) and liver (B) disease.

Supplementary Figure S17. Subgroup analysis of the associations between frailty and depression trajectories and gastrointestinal disease (SHARE).

Variable	Count	Percent	Levels	P value	P for interaction	OR_CI
Overall	5834	100	G1			
			G2	0.001		1.87 (1.28-2.71)
			G3	<0.001		4.26 (2.50-7.26)
sex					0.472	
Female	3021	51.8	G1			
			G2	0.01		2.04 (1.19-3.51)
			G3	<0.001		4.54 (2.16-9.55)
Male	2813	48.2	G1			
			G2	0.04		1.75 (1.03-3.00)
			G3	0.001		4.01 (1.80-8.94)
age_group					0.001	
<=65	4311	73.9	G1			
			G2	<0.001		2.47 (1.59-3.85)
			G3	<0.001		8.69 (4.56-16.57)
>65	1523	26.1	G1			
			G2	0.625		0.84 (0.42-1.69)
			G3	0.944		1.04 (0.39-2.77)
education					0.01	
Below high school	2131	36.5	G1			
			G2	0.13		1.55 (0.88-2.73)
			G3	0.041		2.35 (1.03-5.32)
High school	2100	36	G1			
			G2	0.025		2.08 (1.10-3.95)
			G3	<0.001		8.62 (3.65-20.37)
Above high school	1603	27.5	G1			
			G2	0.064		2.12 (0.96-4.70)
			G3	0.015		4.81 (1.35-17.07)
marriage					0.2	
Other	1491	25.6	G1			
			G2	0.024		2.43 (1.12-5.26)
			G3	0.054		2.97 (0.98-8.96)
Married	4343	74.4	G1			
			G2	0.011		1.75 (1.14-2.71)
			G3	<0.001		5.22 (2.82-9.66)
residence					0.005	
Rural	1866	32	G1			
			G2	0.834		0.93 (0.47-1.83)
			G3	0.003		3.95 (1.58-9.89)
Urban	3968	68	G1			
			G2	<0.001		2.66 (1.67-4.24)
			G3	<0.001		4.78 (2.42-9.46)

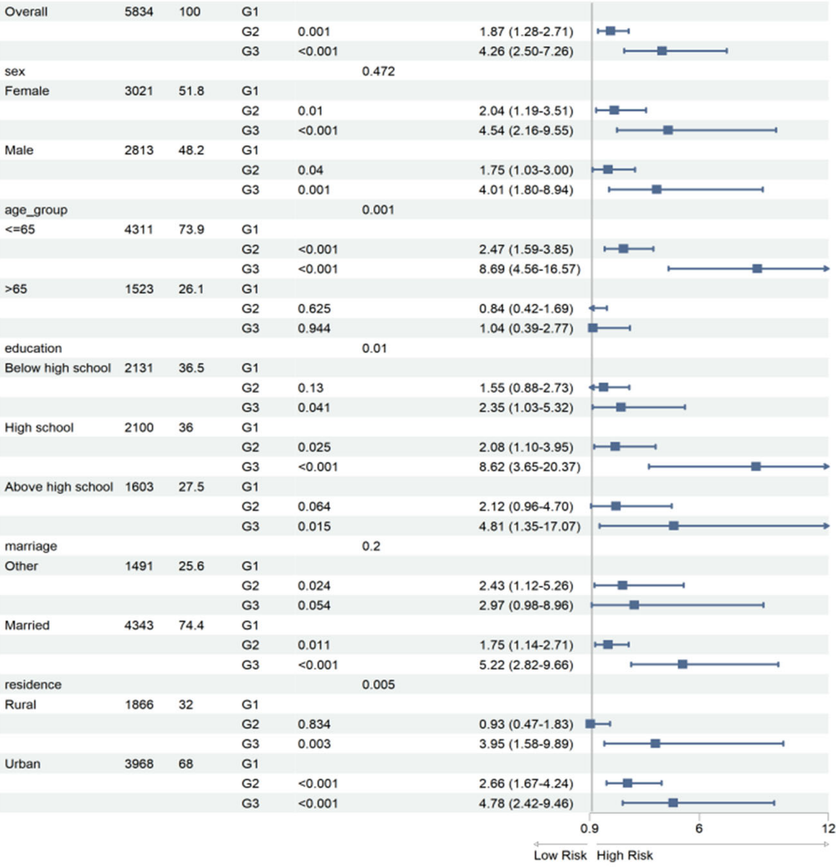

Supplementary Table S7. Sensitivity analysis of frailty and depression trajectories for gastrointestinal and liver disease by discrete survival model by discrete complementary log-log regression.

Characteristics	Model 1		Model 2		Model 3		Model 4	
	HR (95%CI)	P value	HR (95%CI)	P value	HR (95%CI)	P value	HR (95%CI)	P value
Gastrointestinal Disease								
G1	Reference		Reference		Reference		Reference	
G2	1.82(1.65,2.01)	<0.001	1.76(1.58,1.95)	<0.001	1.75(1.58,1.94)	<0.001	1.64(1.46,1.83)	<0.001
G3	2.38(2.05,2.78)	<0.001	2.34(2.00,2.74)	<0.001	2.33(1.99,2.73)	<0.001	2.11(1.75,2.55)	<0.001
Liver Disease								

G1	Reference		Reference		Reference		Reference	
G2	1.76(1.49,2.07)	<0.001	2.02(1.70,2.40)	<0.001	2.04(1.72,2.43)	<0.001	2.04(1.72,2.43)	<0.001
G3	3.01(2.44,3.70)	<0.001	3.68(2.96,4.58)	<0.001	3.77(3.01,4.72)	<0.001	3.77(3.01,4.72)	<0.001

Supplementary Figure S18. Trajectories of frailty and depression from wave 1 to wave 4 excluding participants with gastrointestinal disease and who had used gastrointestinal drugs (CHARLS).

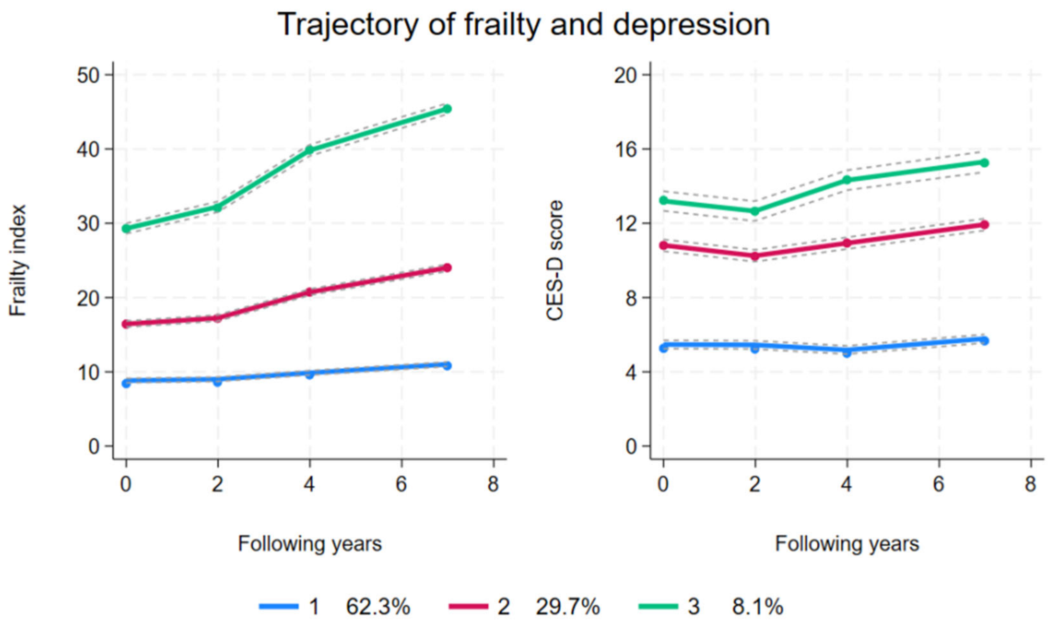

Supplementary Table S8. Group-based trajectory model fit for trajectories of frailty and depression excluding participants with gastrointestinal disease and who had used gastrointestinal drugs (CHARLS).

Note. AvepP: Average Predicted Probability.

Number of classes	Parameters of trajectory shape	Proportions per class%	AvepP	AIC	BIC	ll	Entropy
2	33-33	22.80- 77.20	0.949-0.982	-196579.91	-196665.36	-196560.91	0.914
3	333-333	8.07-62.27	0.911-0.959	-193919.98	-194045.91	-193891.98	0.880
4	3333-3333	2.71-55.49	0.872-0.952	-192914.33	-193080.74	-192877.33	0.859
5	33333-33333	3.71-51.96	0.842-0.936	-192179.25	-192386.13	-192133.25	0.844
Best fit							
3	333-333	8.07-62.27	0.911-0.959	-193919.98	-194045.91	-193891.98	0.880

Supplementary Table S9. The Parameters of trajectories of frailty and depression excluding participants with gastrointestinal disease and who had used gastrointestinal drugs (based on model 333-333) (CHARLS).

**Frailty-Depression**	**Trajectory-Group**	**Parameter**	**Estimate**	**Standard error**	**T value**	**P value**
Frailty	G1	Intercept	8.40093	0.11065	75.926	<0.001
		Linear	-0.20527	0.18707	-1.097	0.2725
		Quadratic	0.18497	0.07501	2.466	0.0137
		Cubic	-0.01519	0.00723	-2.100	0.0357
	G2	Intercept	16.41757	0.18664	87.964	<0.001
		Linear	-0.79414	0.28570	-2.780	0.0054
		Quadratic	0.73482	0.11442	6.422	<0.001
		Cubic	-0.06664	0.01101	-6.050	<0.001

	G3	Intercept	29.29401	0.35374	82.812	<0.001
		Linear	-0.79000	0.55724	-1.418	0.1563
		Quadratic	1.41150	0.22397	6.302	<0.001
		Cubic	-0.13857	0.02163	-6.407	<0.001
Depression	G1	Intercept	4.91830	0.08843	55.621	<0.001
		Linear	0.20458	0.14678	1.394	0.1634
		Quadratic	0.14395	0.05884	-2.446	0.0144
		Cubic	0.01753	0.00567	3.093	0.0020
	G2	Intercept	10.76307	0.13248	81.246	<0.001
		Linear	-0.79472	0.21700	-3.662	0.0003
		Quadratic	0.29892	0.08693	3.439	0.0006
		Cubic	-0.02316	0.00838	-2.764	0.0057
	G3	Intercept	13.18366	0.24006	54.918	<0.001

	Linear	-1.26053	0.40721	-3.096	0.0020
	Quadratic	0.60386	0.16389	3.685	0.0002
	Cubic	-0.05436	0.01584	-3.431	0.0006

Supplementary Table S10. Odds of correct classification for trajectories of frailty and depression excluding participants with gastrointestinal disease and who had used gastrointestinal drugs.

Trajectory group	OCC	OCCw
G1	14.1204	14.3460
G2	24.5926	24.2294
G3	226.0771	224.1839

Note: G1: stable and robust with no depression; G2: moderate persistent frailty and depression; G3: escalating frailty and high depression

OCC: Odds of correct classification;

OCCw: Odds of correct classification using weighted posterior probabilities.

Supplementary Table S11. Binary Logistic Regression models examining the relationship between frailty and depression trajectories and gastrointestinal disease status in 2018 excluding participants with gastrointestinal disease and who had used gastrointestinal drugs.

Variables	Model1		Model2		Model3		Model4	
	OR(95 % CI)	P	OR(95 % CI)	P	OR (95 % CI)	P	OR (95 % CI)	P
Trajectories of frailty and depression (ref: G1)								
G2	2.160(1.873, 2.492)	<0.001	2.143(1.847,2.487)	<0.001	2.141(1.844,2.485)	<0.001	2.119(1.810,2.482)	<0.001

G3	2.871(2.327,3.542)	<0.001	2.960(2.372,3.694)	<0.001	2.957(2.363,3.700)	<0.001	3.244(2.500,4.208)	<0.001
Age			0.988(0.980,0.996)	0.004	0.988(0.979,0.996)	0.005	0.989(0.981,0.998)	0.014
Gender (ref: Male)								
Female			1.249(1.085,1.438)	0.002	1.252(1.086,1.443)	0.002	1.218(0.991,1.496)	0.060
Education (ref: Below high school)								
High school			0.848(0.644,1.117)	0.242	0.849(0.644,1.119)	0.245	0.853(0.647,1.125)	0.261

Above high school	1.242(0.858,1.796)	0.250	1.249(0.862,1.809)	0.240	1.257(0.866,1.825)	0.229
Marital status (ref: Others)						
Married	0.990(0.787,1.246)	0.934	1.037(0.795,1.352)	0.791	1.044(0.799,1.365)	0.752
Residence (ref: Urban)						
Rural	0.987(0.855,1.139)	0.855	0.986(0.852,1.142)	0.853	0.973(0.838,1.129)	0.716
Social work						

(ref: No)				
Yes	0.971(0.848,1.111)	0.666	0.978(0.854,1.121)	0.753
Living alone (ref: No)				
Yes	1.144(0.785,1.666)	0.484	1.168(0.799,1.707)	0.422
Work (ref: No)				
Yes	1.003(0.859,1.172)	0.966	1.000(0.854,0.1.170)	0.995
Self-rated health (ref: Good)				

Fair	1.265(1.066,1.502)	0.007
Poor	1.646(1.168,2.321)	0.004
Smoking status (ref: Never)		
Former	1.056(0.791,1.390)	0.711
Current	0.928(0.755,1.140)	0.475
Drinking status (ref: Never)		

Former	1.076(0.822,1.409)	0.594
Current	1.015(0.852,1.210)	0.864
Hypertension (ref: No)		
Yes	0.792(0.672,0.933)	0.005
Diabetes (ref: No)		
Yes	0.840(0.626,1.127)	0.244
Sleeptime (ref: t ≥ 7.5h/d)		

t < 7.5h/d	1.196(1.030,1.388)	0.019
IADL	0.997(0.907,1.096)	0.951
ADL	0.866(0.780,0.963)	0.008

Supplementary Figure S19. Trajectories of frailty and depression from wave 1 to wave 4 excluding participants with liver disease and who had used liver drugs (CHARLS).

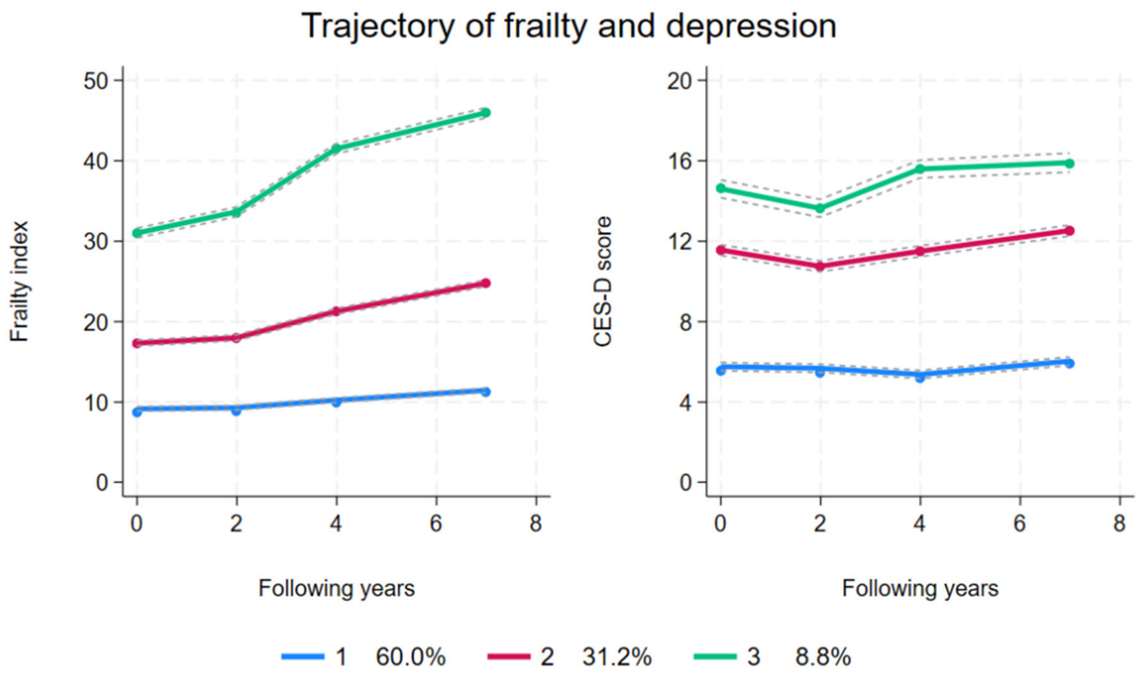

Supplementary Table S12. Group-based trajectory model fit for trajectories of frailty and depression excluding participants with liver disease and who had used liver drugs (CHARLS).

Note. AvepP: Average Predicted Probability.

Number of classes	Parameters of trajectory shape	Proportions per class%	AvepP	AIC	BIC	ll	Entropy
2	33-33	24.68-75.32	0.948-0.981	-259107.77	-259195.72	-259088.77	0.908
3	333-333	8.75-60.03	0.919-0.959	-255343.06	-255472.67	-255315.06	0.881
4	3333-3333	3.33-53.12	0.876-0.953	-253980.81	-254152.08	-253943.81	0.859
5	33333-33333	4.24-49.43	0.841-0.945	-253034.41	-253247.34	-252988.41	0.840
Best fit							
3	333-333	8.75-60.03	0.919-0.959	-255343.06	-255472.67	-255315.06	0.881

Supplementary Table S13. The parameters of trajectories of frailty and depression excluding participants with liver disease and who had used liver drugs (based on model 333-333) (CHARLS).

**Frailty-Depression**	**Trajectory-Group**	**Parameter**	**Estimate**	**Standard error**	**T value**	**P value**
Frailty	G1	Intercept	8.72034	0.10287	84.773	<0.001
		Linear	0.29171	0.17382	-1.678	0.0933
		Quadratic	0.22264	0.06971	3.194	0.0014
		Cubic	-0.01845	0.00672	-2.746	0.0060
	G2	Intercept	17.31633-	0.16152	107.209	<0.001
		Linear	0.81392	0.25195	-3.230	0.0012
		Quadratic	0.69675	0.10096	6.901	<0.001
		Cubic	-0.06117	0.00972	-6.290	<0.001

	G3	Intercept	31.01982-	0.30986	100.108	<0.001
		Linear	1.26267	0.48298	-2.614	0.0089
		Quadratic	1.62366	0.19406	8.367	<0.001
		Cubic	-0.16270	0.01873	-8.688	<0.001
Depression	G1	Intercept	5.25549	0.07973	65.913	<0.001
		Linear	0.16192	0.13251	1.222	0.2217
		Quadratic	-0.14187	0.05313	-2.670	0.0076
		Cubic	0.01794	0.00512	3.504	0.0005
	G2	Intercept	11.52473-	0.11400	101.090	<0.001
		Linear	1.04732	0.18655	-5.614	<0.001
		Quadratic	0.37663	0.07468	5.043	<0.001
		Cubic	-0.02957	0.00720	-4.108	<0.001
	G3	Intercept	14.60390	0.20414	71.540	<0.001

	Linear	-1.84417	0.34505	-5.345	<0.001
	Quadratic	0.83563	0.13830	6.042	<0.001
	Cubic	-0.07798	0.01335	-5.842	<0.001

Supplementary Table S14. Odds of correct classification for trajectories of frailty and depression excluding participants with liver disease and who had used liver drugs.

Trajectory group	OCC	OCCw
G1	15.1246	15.4108
G2	25.5287	24.9779
G3	190.4271	190.8440

Note: G1: stable and robust with no depression; G2: moderate persistent frailty and depression; G3: escalating frailty and high depression

OCC: Odds of correct classification;

OCCw: Odds of correct classification using weighted posterior probabilities.

Supplementary Table S15. Binary Logistic Regression models examining the relationship between frailty and depression trajectories and liver disease status in 2018 excluding participants with liver disease and who had used liver drugs.

Variables	Model1		Model2		Model3		Model4	
	OR(95 % CI)	P	OR(95 % CI)	P	OR (95 % CI)	P	OR (95 % CI)	P
Trajectories of frailty and depression (ref: G1)								
G2	1.625(1.328, 1.987)	<0.001	1.901(1.539,2.347)	<0.001	1.920(1.553,2.373)	<0.001	1.689(1.348,2.115)	<0.001
G3	2.989(2.314,3.862)	<0.001	3.714(2.827,4.880)	<0.001	3.829(2.900,5.054)	<0.001	3.522(2.534,4.895)	<0.001

Age	0.991(0.980,1.002)	0.112	0.993(0.981,1.004)	0.225	0.992(0.980,1.004)	0.181
Gender (ref: Male)						
Female	0.690(1.085,1.438)	0.002	0.701(0.578,0.851)	<0.001	0.775(0.558,1.023)	0.072
Education (ref: Below high school)						
High school	1.191(0.846,1.676)	0.317	1.197(0.849,1.686)	0.305	1.203(0.852,1.697)	0.294
Above high school	1.600(1.028,2.490)	0.037	1.606(1.029,2.505)	0.037	1.531(0.977,2.400)	0.063

Marital status (ref: Others)						
Married	1.303(0.925,1.835)	0.130	1.294(0.873,1.919)	0.199	1.271(0.855,1.888)	0.235
Residence (ref: Urban)						
Rural	0.888(0.730,1.081)	0.236	0.866(0.709,1.058)	0.160	0.868(0.708,1.064)	0.172
Social work (ref: No)						
Yes			1.036(0.8	0.711	1.013(0.8	0.895

	60,1.247)		40,1.221)	
Living alone (ref: No)				
Yes	1.008(0.573,1.773)	0.979	1.027(0.582,1.813)	0.927
Work (ref: No)				
Yes	1.144(0.920,1.423)	0.225	1.163(0.931,1.451)	0.183
Self-rated health (ref: Good)				
Fair			1.663(1.338,2.066)	<0.001

Poor	1.304(0.827,2.057)	0.253
Smoking status (ref: Never)		
Former	1.251(0.884,1.768)	0.206
Current	0.927(0.706,1.218)	0.586
Drinking status (ref: Never)		
Former	1.338(0.964,1.856)	0.081

Current	1.227(0.970,1.552)	0.088
Hypertension (ref: No)		
Yes	1.139(0.923,1.404)	0.226
Diabetes (ref: No)		
Yes	1.217(0.865,1.714)	0.259
Sleeptime (ref: t ≥ 7.5h/d)		
t < 7.5h/d	1.007(0.820,1.236)	0.947

IADL	0.958(0.847,1.083)	0.490
ADL	0.884(0.775,1.009)	0.067

Supplementary Table S16. Baseline characteristics of participants by the status of gastrointestinal diseases in 2017(SHARE).

Variable	Gastrointestinal Diseases in 2017			
	Total (n = 5834)	Not had (n = 5630, 96.5%)	Had (n = 204, 3.5%)	P
Trajectories of frailty and depression, %				<0.001
G1	3591(61.6)	3517(62.5)	74(36.3)	

G2	1773(30.4)	1688(30.0)	85(41.7)	
G3	470(8.1)	425(7.5)	45(22.1)	
Age, years	60.45(8.00)	60.42(8.00)	61.35(7.75)	0.104
BMI	26.48(4.32)	26.45 (4.30)	27.27 (4.89)	0.008
Gender, %				0.846
Male	3021(51.8)	2914(51.8)	107(52.5)	
Female	2813(48.2)	2716(48.2)	97(47.5)	
Education, %				0.037
Below high school	2131(36.5)	2042(36.3)	89(43.6)	
High school	2100(36.0)	2027(36.0)	73(35.8)	
Above high school	1603(27.5)	1561(27.7)	42(20.6)	
Marital status, %				0.982
Married	4343(74.4)	4191(74.4)	152(74.5)	

Other	1491(25.6)	1439(25.6)	52(25.5)	
Residence, %				0.136
Urban	3968(68.0)	3839(68.2)	129(63.2)	
Rural	1866(32.0)	1791(31.8)	75(36.8)	
Living alone, %				0.711
Yes	1204(20.6)	1164(20.7)	40(19.6)	
No	4630(79.4)	4466(79.3)	164(80.4)	
Self-rated health, %				<0.001
Poor	225(3.9)	207(3.7)	18(8.8)	
Fair	1181(20.2)	1108(19.7)	73(35.8)	
Good	2352(40.3)	2283(40.6)	69(33.8)	
Nice	1453(24.9)	1415(25.1)	38(18.6)	
Excellent	623(10.7)	617(11.0)	6(2.9)	

Smoking status, %				0.825
Never	2883(49.4)	2778(49.3)	105(51.5)	
Former	1931(33.1)	1867(33.2)	64(31.4)	
Current	1020(17.5)	985(17.5)	35(17.2)	
Drinking status, %				0.357
Never	8(0.1)	1(0.5)	7(0.1)	
Former	1570(26.9)	57(27.9)	1513(26.9)	
Current	4256(73.0)	146(71.6)	4110(73.0)	
Hypertension, %				0.012
Yes	2532(43.4)	106(52.0)	2426(43.1)	
No	3302(56.6)	98(48.0)	3204(56.9)	
Diabetes, %				0.014

Yes	612(10.5)	32(15.7)	580(10.3)	
No	5222(89.5)	172(84.3)	5050(89.7)	
Work, %				<0.001
Yes	2256(38.7)	51(25.0)	2205(39.2)	
No	3578(61.3)	153(75.0)	3425(60.8)	
IADL	0.10(0.44)	0.13(0.42)	0.10(0.44)	0.240
ADL	0.14(0.48)	0.25(0.61)	0.13(0.48)	0.001

Supplementary Table S17. Group-based trajectory model fit for trajectories of frailty and depression (SHARE).

Number of classes	Parameters of trajectory shape	Proportions per class%	AvepP	AIC	BIC	ll	Entropy
2	33-33	22.35-77.65	0.955-0.987	-126898.29	-126981.42	-126879.29	0.931
3	333-333	8.08-61.27	0.933-0.967	-123924.02	-124047.92	-123897.41	0.904
4	3333-3333	3.08-50.58	0.906-0.968	-122516.21	-122678.10	-122479.21	0.885
5	33333-33333	1.54-41.95	0.873-0.970	-121900.01	-122101.28	-121854.01	0.859
Best fit							
3	332-111	8.08-61.27	0.932-0.967	-123924.02	-124015.90	-123903.02	0.904

Supplementary Table S18. The parameters of trajectories of frailty and depression (based on model 332-111) (SHARE).

**Frailty-Depression**	**Trajectory-Group**	**Parameter**	**Estimate**	**Standard error**	**T value**	**P value**
Frailty	G1	Intercept	7.35843	0.11635	63.246	<0.001
		Linear	-0.37601	0.20928	-1.797	0.0724
		Quadratic	0.43528	0.09234	4.714	<0.001
		Cubic	-0.04547	0.01014	-4.483	<0.001
	G2	Intercept	17.10522	0.19088	89.612	<0.001
		Linear	0.57637	0.30148	1.912	0.0559
		Quadratic	0.29815	0.13277	2.246	0.0247
		Cubic	-0.03062	0.01459	-2.099	0.0358
	G3	Intercept	30.82751	0.32741	94.155	<0.001

		Linear	2.35915	0.24213	9.743	<0.001
		Quadratic	0.07488	0.03875	1.933	0.0533
Depression	G1	Intercept	0.76678	0.03397	22.571	<0.001
		Linear	0.00737	0.00861	0.855	0.3924
	G2	Intercept	2.61078	0.04800	54.395	<0.001
		Linear	0.05320	0.01189	4.474	<0.001
	G3	Intercept	3.97374	0.08635	46.019	<0.001
		Linear	0.06758	0.02256	2.996	0.0027

Supplementary Table S19. Odds of correct classification for trajectories of frailty and depression (SHARE).

Trajectory group	OCC	OCCw
G1	18.3668	18.5877
G2	31.3280	30.9539
G3	294.2368	293.1072

Supplementary Table S20. Association of frailty and depression trajectories with new-onset gastrointestinal diseases of SHARE (discrete survival model).

Characteristics	Model 1		Model 2		Model 3		Model 4	
	HR(95%CI)	P value	HR(95%CI)	P value	HR(95%CI)	P value	HR(95%CI)	P value
Gastrointestinal Disease								
G1	Reference		Reference		Reference		Reference	
G2	0.86(0.55,1.17)	<0.001	2.42(1.72,3.42)	<0.001	2.28(1.62,3.22)	<0.001	1.94(1.31,2.89)	<0.001

G3	1.57(1.20,1.94)	<0.001	5.10(3.32,7.84)	<0.001	4.73(3.08,7.25)	<0.001	3.94(2.32,6.69)	<0.001

Model 1: Crude Model;

Model 2: Adjusted for age, gender, education, marital status, residence, BMI;

Model 3: Adjusted for age, gender, education, marital status, residence, BMI, living alone, work;

Model 4: Adjusted for age, gender, education, marital status, residence, BMI, living alone, work, self-rated health, smoking status, drinking status, hypertension, diabetes, IADL, ADL.

Supplementary Figure S20. Trajectories of frailty and depression from wave 1 to wave 4 excluding participants with gastrointestinal disease and who had used gastrointestinal drugs (SHARE).

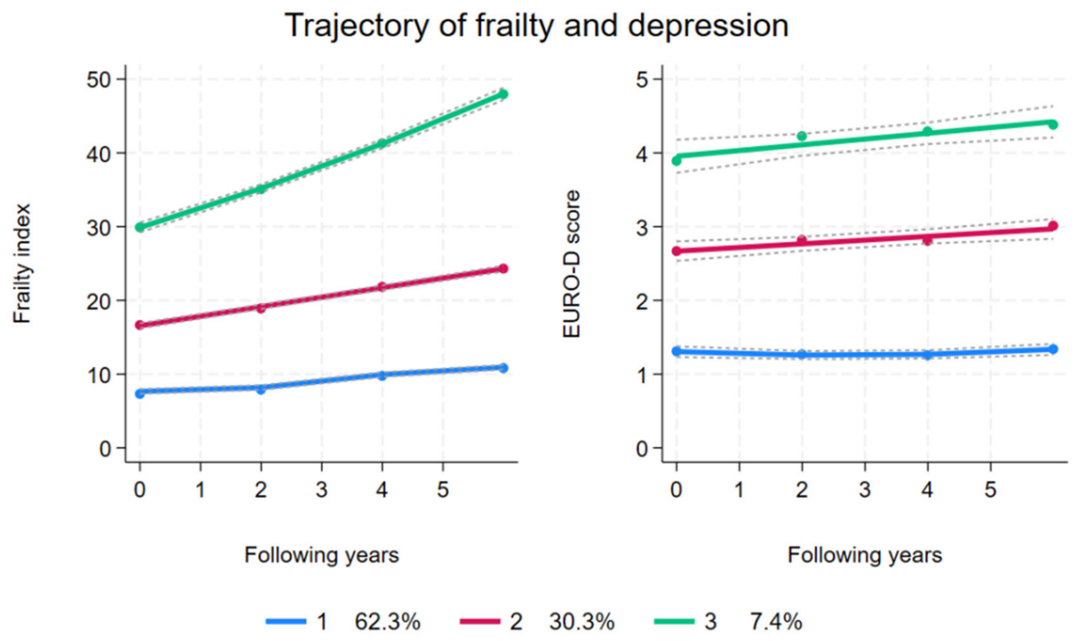

Supplementary Table S21. Group-based trajectory model fit for trajectories of frailty and depression excluding participants with gastrointestinal disease and who had used gastrointestinal drugs (SHARE).

Note. AvepP: Average Predicted Probability.

Number of classes	Parameters of trajectory shape	Proportions per class%	AvepP	AIC	BIC	ll	Entropy
2	33-33	22.35-77.65	0.955-0.987	-126898.29	-126981.42	-126879.29	0.931
3	333-333	8.08-61.27	0.933-0.967	-123924.02	-124047.92	-123897.41	0.904
4	3333-3333	3.08-50.58	0.906-0.968	-122516.21	-122678.10	-122479.21	0.885
5 Best fit	33333-33333	1.54-41.95	0.873-0.970	-121900.01	-122101.28	-121854.01	0.859

3	332-111	8.08-61.27	0.932-0.967	-123924.02	-124015.90	-123903.02	0.904

Supplementary Table S22. The parameters of trajectories of frailty and depression excluding participants with gastrointestinal disease and who had used gastrointestinal drugs (based on model 312-211) (SHARE).

**Frailty-Depression**	**Trajectory-Group**	**Parameter**	**Estimate**	**Standard error**	**T value**	**P value**
Frailty	G1	Intercept	7.25706 -	0.11716	61.944	<0.001
		Linear	0.39935	0.21058	-1.896	0.0579
		Quadratic	0.43955	0.09291	4.731	<0.001
		Cubic	-0.04568	0.01021	-4.476	<0.001
	G2	Intercept	16.55073	0.17338	95.461	<0.001
		Linear	1.29401	0.03772	34.306	<0.001

	G3	Intercept	29.88339	0.34254	87.241	<0.001
		Linear	2.47028	0.25495	9.689	<0.001
		Quadratic	0.09234	0.04084	2.261	0.0238
Depression	G1	Intercept	0.79325	0.03988	19.891	<0.001
		Linear	-0.05875	0.03077	-1.909	0.0562
		Quadratic	0.01111	0.00491	2.262	0.0237
	G2	Intercept	2.54693	0.04995	50.992	<0.001
		Linear	0.05599	0.01236	4.529	<0.001
	G3	Intercept	3.92750	0.09255	42.436	<0.001
		Linear	0.08006	0.02427	3.298	<0.001

Supplementary Table S23. Odds of correct classification for trajectories of frailty and depression excluding participants with gastrointestinal disease and who had used gastrointestinal drugs (SHARE).

Trajectory group	OCC	OCCw
G1	17.1354	17.3761
G2	32.0271	31.5016
G3	320.4718	321.4718

Supplementary Table S24. Binary Logistic Regression models examining the relationship between frailty and depression trajectories and gastrointestinal disease status in 2017 excluding participants with gastrointestinal disease and who had used gastrointestinal drugs (SHARE).

Variables	Model1		Model2		Model3		Model4	
	OR (95 % CI)	P	OR (95 % CI)	P	OR (95 % CI)	P	OR (95 % CI)	P
Trajectories of frailty and depression (ref: G1)								
G2	2.418(1.714, 3.413)	<0.001	2.445(1.701,3.513)	<0.001	2.305(1.599, 3.922)	<0.001	2.026(1.351, 3.040)	<0.001

G3	5.324(3.498, 8.102)	<0.001	5.489(3.437,8 .767)	<0.001	5.139(3.217, 8.210)	<0.001	4.573(2.540, 8.233)	<0.001
Age			0.989(0.969,1 .009)	0.288	0.979(0.958, 1.002)	0.067	0.983(0.959, 1.007)	0.157
BMI			1.000(0.966,1 .036)	0.984	0.999(0.964, 1.034)	0.935	0.997(0.961, 1.034)	0.883
Gender (ref: Female)								
Male			1.126(0.819,1 .549)	0.465	1.152(0.836, 1.589)	0.387	1.129(0.800, 1.593)	0.490
Education (ref: Below high school)								

High school	0.938(0.662,1.329)	0.717	0.975(0.687, 1.384)	0.888	1.012(0.710, 1.442)	0.948
Above high school	0.627(0.401,0.981)	0.041	0.670(0.427, 1.052)	0.082	0.703(0.445, 1.109)	0.129
Marital status (ref: Others)						
Married	1.098(0.765,1.575)	0.611	0.931(0.553, 1.567)	0.787	0.904(0.535, 1.529)	0.707
Residence (ref: Urban)						
Rural	1.096(0.792,1.516)	0.581	1.091(0.788, 1.509)	0.600	1.095(0.790, 1.519)	0.587
Living alone						

(ref: No)				
Yes	0.806(0.449, 1.444)	0.468	0.788(0.437, 1.420)	0.428
Work (ref: No)				
Yes	0.627(0.412, 0.952)	0.028	0.639(0.420, 0.972)	0.036
Self-rated health (ref: Poor)				
Fair			0.889(0.472, 1.675)	0.716
Good			0.602(0.305, 1.188)	0.144

Nice	0.714(0.336, 1.520)	0.382
Excellent	0.269(0.087, 0.832)	0.023
Smoking status (ref: Never)		
Former	0.968(0.674, 1.390)	0.859
Current	0.910(0.583, 1.419)	0.677
Drinking status (ref: Never)		

Former	0.435(0.048, 3.972)	0.461
Current	0.451(0.050, 4.108)	0.480
Hypertension (ref: No)		
Yes	0.906(0.648, 1.266)	0.563
Diabetes (ref: No)		
Yes	0.905(0.566, 1.446)	0.675
IADL	0.682(0.445,	0.074

	1.038)	
ADL	1.018(0.741, 1.397)	0.913
